# Biomarkers of Metabolomics in Inflammatory Bowel Disease and Damp-Heat Syndrome: A Preliminary Study

**DOI:** 10.1155/2022/3319646

**Published:** 2022-07-01

**Authors:** Xingxing Wu, Kexin Liu, Qi Wu, Mao Wang, Xuelian Chen, Yuge Li, Lin Qian, Changyin Li, Guoliang Dai, Qide Zhang, Genglin Mu, Jing Wu, Zhaowei Shan

**Affiliations:** ^1^Gastroenterology Department, Affiliated Hospital of Nanjing University of Chinese Medicine, Nanjing 210029, China; ^2^Ethics Committee, Affiliated Hospital of Nanjing University of Chinese Medicine, Nanjing 210029, China; ^3^Clinical Pharmacology Laboratory, Affiliated Hospital of Nanjing University of Chinese Medicine, Nanjing 210029, China; ^4^Digestive Endoscopy Center, Affiliated Hospital of Nanjing University of Chinese Medicine, Nanjing 210029, China; ^5^Institute of Chinese Medicine, Nanjing University, Nanjing Drum Tower Hospital, Drum Tower Clinical Medicine College of Nanjing University of Chinese Medicine, Nanjing 210008, China

## Abstract

**Aims:**

This study aims to investigate the potential biomarkers of inflammatory bowel disease (IBD) and IBD with damp-heat syndrome (IBD-DH) by metabolomics.

**Methods:**

Plasma and urine samples were collected from 15 healthy controls and 30 IBD patients, including 15 IBD-DH and 15 IBD with spleen deficiency syndrome (IBD-SD), which was coded as SF8G and SF70 according to the International Classification of Diseases Eleventh Revision (ICD-11) issued by World Health Organization. Pseudotargeted metabolomics method was used based on ultra-high-performance liquid chromatography-high-resolution mass spectrometry and triple-quadrupole mass spectrometry.

**Results:**

Under the condition of false discovery rate (FDR) < 0.05, variable importance projection (VIP) > 1.0, and fold change (FC) > 1.5 or < 2/3, we found 57 plasma differential metabolites and 20 urinary differential metabolites in IBD. Then, with area under the curve (AUC) ≥ 0.85 and FC ≥ 2 or ≤ 0.3, 11 potential biomarkers were identified, such as acylcarnitine (ACar 20:4, ACar 18:1, and ACar 20:3), 3-indoleacetic acid, hippuric acid, and dehydroepiandrosterone sulfate, which is related to intestinal microbiota and immune response. However, less obvious differences were observed in IBD-DH when compared with IBD-SD. Under the condition of FDR < 0.2, VIP >1.0, and FC > 1.5 or < 2/3, we identified 16 plasma differential metabolites. In urine samples, IBD-DH and IBD-SD had the same metabolite pattern. With AUC ≥ 0.80, 7 differential plasma metabolites, mainly glycerophospholipids, were identified in IBD-DH. Kyoto Encyclopedia of Genes and Genomes analysis indicated that metabolic pathways, such as citrate cycle and amino acids metabolism, were mainly responsible for the distinction between IBD and healthy controls, whereas glycerophospholipid metabolism perturbation was not only a manifestation of IBD but also an important pathway to distinguish two subtypes defined by traditional medicine, IBD-DH and IBD-SD.

**Conclusion:**

In this study, we found that several metabolites of aromatic acids and lipid derivatives could act as potential biomarkers to discriminate IBD from healthy controls. Glycerophospholipids metabolites might be used to differentiate IBD-DH from IBD-SD.

## 1. Introduction

Inflammatory bowel disease (IBD) is a chronic, idiopathic, and relapsing noninfectious inflammatory condition of the gastrointestinal tract with two main phenotypes: ulcerative colitis and Crohn's disease. Epidemiological studies have reported that IBD has affected around 0.3% of the world's population or more than 20 million people in recent years [1]. The pathophysiology of IBD involves complex genetic, environmental, epithelial, microbial, and immune factors [2]. The IBD armamentarium includes untargeted therapies and targeted biologic therapies. Although biologic therapies, including antitumor necrosis factor therapy, are effective in many patients, up to 30% of patients do not have a response to initial treatment, and in up to 50% of patients, the response is lost over time [2]. At the same time, evidence has shown that herbal medicine and herbal medicine formulas exhibit efficacy in treating IBD [3]. From the perspective of Chinese medicine, the Consensus from the China Association of Chinese Medicine defines IBD as a damp-heat syndrome (IBD-DH) and IBD with spleen deficiency syndrome (IBD-SD). They are the two main subtypes of the active IBD syndromes [4, 5]. The main clinical manifestations of IBD-DH are abdominal pain, diarrhea, bloody stool with mucus and pus, and tenesmus. The main clinical manifestations of IBD-SD are bloody purulent stool, more white (pus and mucus) and less red (blood) or just white (pus and mucus), abdomen dull pain, diarrhea and loose stools with indigestible food, epigastric fullness, and abdominal distension. Meanwhile, the International Classification of Diseases Eleventh Revision (ICD-11) issued by the World Health Organization included the coding rules of traditional medicine [6]. It was expected to provide sufficient evidence for this diagnosis standards. However, few studies have been performed on the metabolic characteristics of IBD-DH patients. In order to characterize IBD metabolic profiles and explore the biomarkers of IBD-DH, the pseudotargeted metabolomics approach was employed to analyze blood and urine samples collected from IBD patients and healthy volunteers. We expected to provide further evidence for herbal therapy and possible reasons for nonresponders of conventional therapy.

Metabolomics is a component of systematic biology that aims to comprehensively assess metabolites in organisms [7]. Metabolomics has great promise for diagnosis, prediction, and personalized treatment in clinical practice [8, 9]. More than this, a metabolomic method was also used to reflect the disease state [10], distinguish spleen and stomach deficiency cold syndrome and spleen and stomach damp-heat syndrome in chronic atrophic gastritis [11], explain the increased risk of cardiovascular disease in IBD patients [12], and monitor mucosal inflammation [13]. Untargeted and targeted metabolomics were mainly used in these researches. Pseudotargeted metabolomics has both advantages of them with the development of metabolomics technology. It has wide coverage and high performance of applications in metabolomics studies [14]. Many researchers have finished works based on pseudotargeted metabolomics to study the biochemistry of disease [15, 16], screen biomarker [17, 18], and demonstrate the metabolites of Chinese herbal medicine [19, 20]. Therefore, pseudotargeted metabolomics was an effective tool to demonstrate the characteristics of Chinese IBD patients, especially the damp-heat syndrome subtype defined by traditional medicine.

## 2. Materials and Methods

### 2.1. Participants

All subjects (18∼75 years old) were recruited from the Affiliated Hospital of Nanjing University of Chinese Medicine from May 2020 to March 2021. Clinical disease activity of IBD patients (*n* = 30) was confirmed according to previously established clinical, laboratory examination, radiological, and endoscopic criteria, as well as histological findings [21]. Spleen deficiency syndrome (SF70) (*n* = 15) and damp-heat syndrome (SF8G) (*n* = 15) in IBD patients were diagnosed by clinical manifestations, also referring to the tongue coating and pulse condition [22] (their diagnosis standards in detail were listed in Supplementary Material 1). IBD subjects were excluded if they had used corticosteroids, antibiotics, biological therapy, or immunological therapy or had a history of colectomy, hypertension, diabetes mellitus, or immune diseases. A group of healthy adult volunteers (Con) (*n* = 15) with no evidence of hypertension, diabetes mellitus, and autoimmune conditions were enrolled. There were no significant abnormalities in blood, urine, fecal routine tests, biochemical analysis of liver and kidney, and electrocardiogram results in healthy individuals. Medical history was normal.

Informed consent was obtained from all participants, and the study was approved by the Ethics Committee at Affiliated Hospital of Nanjing University of Chinese Medicine (2020NL-058-01) and China Clinical Trial Registry (ChiCTR2100045758. Registered 24 April 2021-Retrospectively registered, http://www.chictr.org.cn/index.aspx).

### 2.2. Sample Collection

To minimize the dietary influence, blood and second morning urine samples were obtained from all subjects after overnight fasting [23]. Plasma (200 *µ*L) was separated by centrifugation at 2500 *g* for 5 min and frozen at −80°C until use. Urine (1 mL) was dispensed and frozen at −80°C until used for metabolomic analysis, and the remaining urine was used for urine specific gravity testing at the Laboratory Department of the Affiliated Hospital of Nanjing University of Chinese Medicine.

### 2.3. Reagents and Materials

Liquid chromatography-mass spectrometry grade methanol, formic acid, and acetonitrile were purchased from Fisher Scientific (USA). Ultrapure water was generated employing a Milli-Q Integral Water Purification System from Merck Millipore (Merck Millipore, USA).

### 2.4. Sample Separation

Samples were allowed to thaw on ice. 200 *µ*L of methanol (precooled at −20°C, containing 13 internal standards with the concentrations as follows: carnitine C2:0-d3: 0.08 *µ*g/mL; carnitine C8:0-d3: 0.05 *µ*g/mL; carnitine C10:0-d3: 0.05 *µ*g/mL; carnitine C16:0-d3: 0.075 *µ*g/mL; LPC 19:0: 0.375 *µ*g/mL; FFA C16:0-d3: 1.25 *µ*g/mL; FFA C18:0-d3: 1.25 *µ*g/mL; CDCA-d4: 0.375 *µ*g/mL; CA-d4: 0.925 *µ*g/mL; L-Trp-D5: 2.125 *µ*g/mL; L-Phe-D5: 1.8 *µ*g/mL; SM (12:0): 0.375 *µ*g/mL; choline-d4: 1.0 *µ*g/mL [14]) was added to 50 *µ*L of plasma for protein precipitation. The mixture was homogenized (Vortex, 1 min) at room temperature, stood at 4°C for 10 min, and centrifuged at 14000 *g* and 4°C for 15 min. 200 *µ*L of the supernatant was transferred into a new EP tube, concentrated by 4°C centrifugation, and stored at −20°C. Prior to analysis, the concentrated metabolite extract sample was reconstituted in 100 *µ*L of 20% methanol/water solution, vortex-mixed, and then centrifuged at 14000 *g* and 4°C for 15 min, and 70 *µ*L of supernatant was transferred to a liquid chromatography sample vial for liquid chromatography-mass spectrometry analysis [14, 24].

### 2.5. Quality Control (QC) Samples and Blank Sequences

A blank extract was prepared following the same procedure, but plasma was replaced with 20% methanol/water. For quality control, an equal amount of each sample extract was pooled in a glass vial to create a quality control sample. The pretreatment of QC samples was the same as that of analyzed samples. QC sample and blank extract were inserted every ten samples to monitor the stability of the system while running the sequences. The whole batch analysis contained six blank extracts and six QC samples.

### 2.6. Data Acquisition

#### 2.6.1. Selection of Ion-Pairs Information by Ultra-High-Performance Liquid Chromatography-High-Resolution Mass Spectrometry (UHPLC-HRMS)

To obtain comprehensive metabolite information, two QC samples were subjected to untargeted metabolomics analysis based on UHPLC-HRMS (Supplementary Material 1) [14]. Based on the multilevel metabolite characterization system (Supplementary Material 1), we used the One-MAP platform (http://www.5omics.com/, version 1.0) to integrate and analyze the results of qualitative annotation analysis at different levels to obtain preliminary qualitative results. Accordingly, ion pairs from metabolites were screened from the above untargeted qualitative data. Test different collision energies with the AB5500 mass spectrometer to obtain the most suitable collision energy. The ion-pair list from previously targeted assay compounds (e.g., citrate cycle and glycolysis), declustering potential (DP), and collision energy were obtained to supplement the ion-pair list for this study. Based on the ion-pairs list (Supplementary Material 2), all samples were analyzed by the following ultra-high-performance liquid chromatography-triple-quadrupole mass spectrometry (UHPLC-TQMS) method [14].

#### 2.6.2. Multireaction Monitoring (MRM) Analysis by UHPLC-TQMS

UHPLC-TQMS MRM analysis was performed on QTRAP 5500 mass spectrometry (AB SCIEX, USA) coupled with an Acquity liquid chromatography system (AB SCIEX, USA).

*(1) Chromatographic Conditions*. Under positive-ion mode, chromatographic column was Waters BEH C8 column (1.7 *µ*m, 2.1 × 100 mm); column temperature was 50°C; injection volume: 5 *µ*L; flow rate was 0.35 mL/min; mobile phase A was 0.1% formic acid/water; mobile phase B was 0.1% formic acid/acetonitrile; gradient elution procedure was 5% of phase B as the starting concentration, 0–1 min; phase B changes from 5% to 100%, 1.1–11 min; phase B remains at 100%, 11.1–13 min; phase B remains at 5%, 13.1–15 min.

Under negative-ion mode, chromatographic column was Waters HSS T3 column (1.8 *µ*m, 2.1 × 100 mm); column temperature was 50°C; injection volume was 5 *µ*L; flow rate was 0.35 mL/min; mobile phase A was 0.1% formic acid/water; mobile phase B was 0.1% formic acid/acetonitrile; gradient elution procedure was 5% of phase B as the starting concentration, 0–1 min; phase B changes from 5% to 100%, 1.1–11 min; phase B remains at 100%, 11.1–13 min; phase B remains at 5%, 13.1–15 min.

*(2) Mass Spectrometry Conditions.* Under positive-ion mode, mass spectrometry primary full scan + data-dependent acquisition (DDA) secondary ion scan and Turbo V™ ion source positive mode with primary full scan + secondary ion scan mode were used. Experiment type was MRM; vacuum gauge was 2.4 × 10e^−5^ Torr; MRM detection window was 55 s; target scan time was 0.8 s; collision gas was medium; curtain gas was 35.0 psi; ion source gas1 was 60.0 psi; ion source gas2 was 60.0 psi; ion spray voltage was 5500 V; temperature was 550°C; collision cell exit potential was 13.0 V; DP was 80.0 V; entrance potential was 10.0 V; scan speed was 10 Da/s; mass range (*m/z*) ion source gas 300–2000; full MS resolution was 70000; MS/MS resolution was 17500.

Under negative-ion mode, mass spectrometry primary full scan + DDA secondary ion scan and Turbo V™ ion source negative mode with primary full scan + secondary ion scan mode were used. Experiment type was MRM; vacuum gauge was 2.5 × 10e^−5^ Torr; MRM detection window was 90 s; target scan time was 0.8 s; collision gas was medium; curtain gas was 35.0 psi; ion source gas1 was 60.0 psi; ion source gas2 was 60.0 psi; ion spray voltage was 4500 V; TEM was 500°C; collision cell exit potential was −15.0 V; DP was −80.0 V; entrance potential was −10.0 V; scan speed was 10 Da/s; mass range (*m/z*) was 300–2000; full MS resolution was 70000; MS/MS resolution was 17500.

After the instrument is stabilized, one blank sample information is collected first, then two QC sample information is collected, then the actual sample information is started to be collected, and thereafter one QC sample is collected for every ten actual samples and interspersed. After all samples are collected, one QC sample is collected at the end. All measurement data were collected by Analyst® TF data collection software (version 1.6.3, AB SCIEX, USA).

Pseudotargeted metabolomics of urine samples was described in Supplementary Material 3, and Supplementary Material 4 shows the ion-pairs information of urine samples based on the UHPLC-HRMS method.

### 2.7. Data Processing and Statistical Analysis

#### 2.7.1. Data Preprocessing Process

In the first step, data were obtained by extracting UHPLC-HRMS using XCMS software (version 3.8.2), then peak matching and retention time drift correction were performed, and finally, peak alignment was performed after filling in the missing values to obtain the peak table in One-MAP for characterization. In the second step, the OS software (version 1.5.0.23389) was used to analyze the data obtained by UHPLC-TQMS, firstly by matching, filling in the missing values, and then selecting the target characteristic peaks and quantitatively analyzing the peak areas. In the process of extracting the data, the software simultaneously completes the noise filtering, retention time, peak type identification, calculation of peak area, and other normalization processes according to the preset parameters. After manual compounding and excluding abnormal compounds, statistical analysis was performed.

#### 2.7.2. Statistical Analysis

The differences in age and body mass index (BMI) between the IBD groups and the control group were analyzed by Student's *t*-test. Gender distribution was assessed by Pearson's chi-squared test. SPSS version 22.0 software (IBM, Armonk, NY, USA) was used for statistical analysis, and *p* < 0.05 was considered statistically significant.

**Figure 1:**
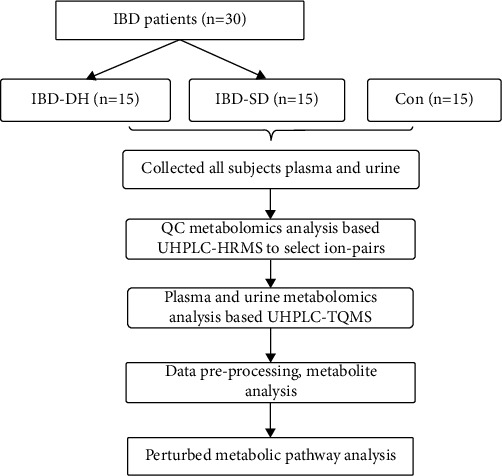
Flowchart of pseudotargeted metabolomics study on inflammatory bowel disease. IBD: inflammatory bowel disease; IBD-DH: IBD with damp-heat syndrome; IBD-SD: IBD with spleen deficiency syndrome; Con: healthy controls.

For metabolomics analysis, partial least squares discriminant analysis (PLS‐DA) was used to discriminate the differences in metabolic profile between the two groups. Two-sample unpaired *t*-test was used to show which metabolites have the power to differentiate the different groups in the data set; a *p*-value < 0.05 was used as a threshold. We controlled false-positive results by Benjamini–Hochberg false discovery rate (FDR). FDR < 0.05 or <0.2 as the threshold was used as needed. The FC analysis serves as a measure for the relative change of metabolite in the different conditions. Fold change (FC) > 1.5 or < 2/3 was set as a threshold. The variable importance projection (VIP) > 1.0 is the threshold cut-off to determine whether metabolic features contribute to distinguishing the two groups. When compared with Con, metabolites with VIP > 1.0, FC > 1.5 or <2/3, and FDR < 0.05 were considered to be differential metabolites in IBD. Metabolites with VIP > 1.0, FC > 1.5 or < 2/3, and FDR < 0.2 were considered to be differential metabolites in IBD-DH when compared with IBD-SD. Differential metabolites with the receiver operating characteristic (ROC) area under the curve (AUC) ≥ 0.85 and FC ≥ 2 or ≤0.3 were considered to be metabolites with diagnostic potential. In this study, metabolic pathways were annotated based on the Kyoto Encyclopedia of Genes and Genomes (KEGG) database, and the differential metabolites were entered into the online tool MetaboAnalyst (version 5.0) for biological pathway analysis, followed by manual correction. Finally, Pathview software (version 3.8) was used to create metabolic pathway visualization figures. The research flowchart is shown in Figure 1.

## 3. Results

### 3.1. Clinical Characteristics of the Subjects

Clinical and demographic data are summarized in Table 1. No statistically significant differences (*p* > 0.05) in age, gender distribution, body mass index, and urine specific gravity were found between the IBD and Con, IBD-DH, and IBD-SD. Among 30 IBD patients, 86.7% of patients accepted 5-aminosalicylic acid treatment. Because of other reasons, such as allergies and medical conditions, 13.3% of patients were mainly treated with Chinese medicine before enrolling into the study.

### 3.2. Metabolomics Profile of IBD versus Con

**Figure 2:**
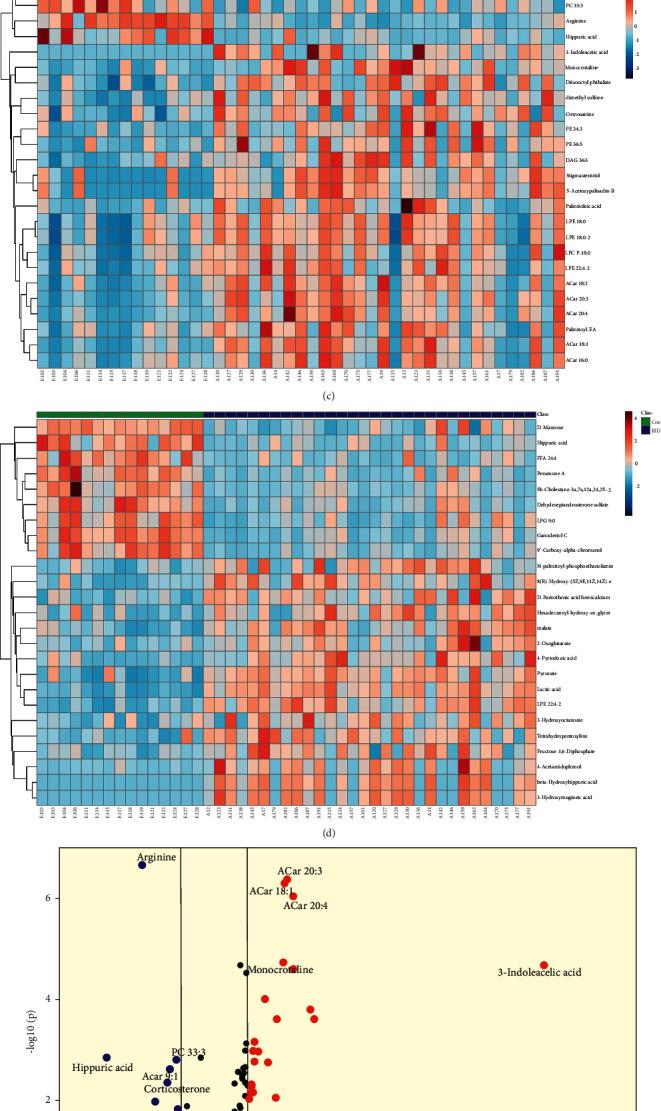
(a), (b) Plasma PLS-DA scores scatter plot. (c), (d) Plasma heatmap analysis (green: healthy controls; blue: inflammatory bowel disease). (e), (f) Volcanic map analysis (blue dot represents metabolite of downregulation, and red dot represents metabolite of upregulation). (a), (c), and (e) Positive-ion mode. (b), (d), and (f) Negative-ion mode.

From the plasma PLS-DA score plots (Figures 2(a) and 2(b)), we found that the metabolic characteristics of the IBD group and the Con group were significantly different. Positive-ion mode is as follows: R^2^Y = 0.83, Q^2^Y = 0.77, and Q^2^*X*_0_ = −0.38 (R^2^Y represents the explanatory rate of the model for Y categorical variables, Q^2^Y represents the predictive power of the model for Y categorical variables; Q^2^X represents the intercept of Q^2^ on the *y*-axis; if Q^2^X < 0, the model is not overfitted). Negative-ion mode is as follows: R^2^Y = 0.85, Q^2^Y = 0.83, and Q^2^*X*_0_ = −0.37. Through heatmap analysis (Figures 2(c) and 2(d), we can observe a distinct metabolic profile between IBD and Con in plasma samples. As demonstrated in the visible volcano plot, some metabolites were upregulated, such as acetylcarnitine (ACar 18:1, ACar 20:4, and ACar 20:3), 3-indoleacetic acid, malate, and L-glutamic acid; some were downregulated (e.g., arginine, hippuric acid, and lysyl-phosphatidylglycerol (LPG 9:0)). Under the conditions of FC > 1.5 or < 2/3, VIP > 1.0, and FDR < 0.05, we finally screened out 57 differential metabolites in plasma samples (Supplementary Material 5 and Table S1) in IBD compared to Con. Among the 57 differential plasma metabolites, there were mainly 18 fatty acyls, 6 glycerophospholipids (including phosphatidylethanolamine (PE 34:3; PE 36:5), phosphatidylcholine (PC 33:3), LPG 9:0, lyso-phosphatidylethanolamine (LPE 22:4–2), and LPC P-18:0), 7 steroids and steroid derivatives, and 5 amino acids and host-symbiotic intestinal flora metabolites (e.g., 3-indoleacetic acid, beta-hydroxyhippuric acid, and hippuric acid).

**Figure 3:**
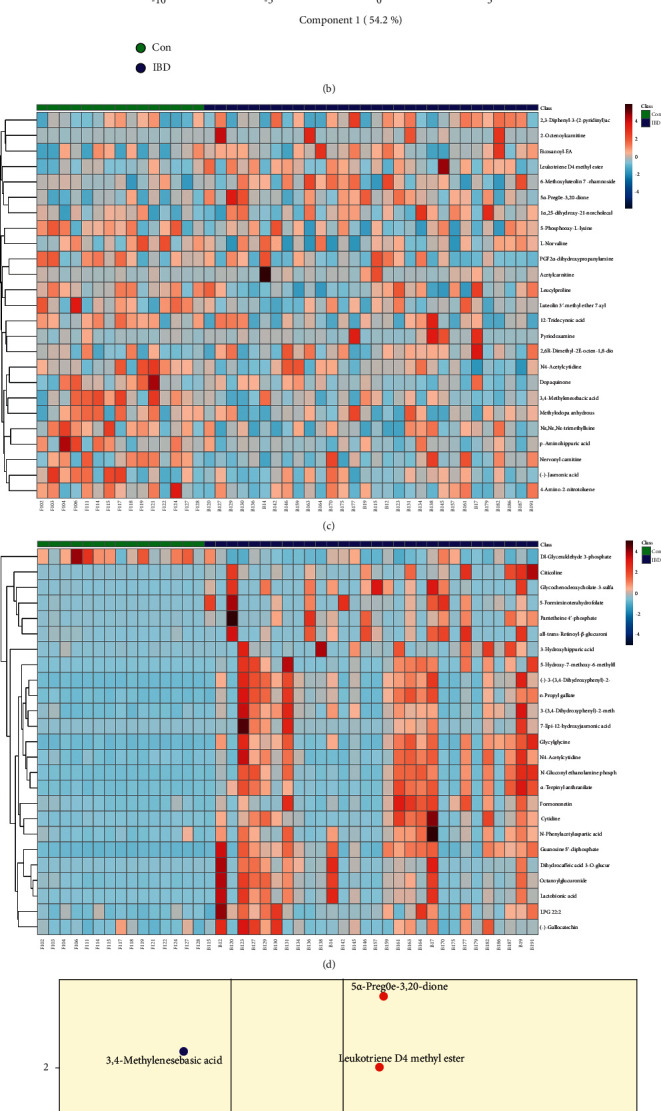
(a), (b) Urine PLS-DA scores scatter plot. (c), (d) Urine heatmap analysis (green: healthy controls; blue: inflammatory bowel disease). (e), (f) Volcanic map analysis (blue dot represents metabolite of downregulation, and red dot represents metabolite of upregulation). (a), (c), and (e) Positive-ion mode. (b), (d), and (f) Negative-ion mode.

In urine samples, from the PLS-DA plots (Figures 3(a) and 3(b)), we found that IBD and Con were clearly overlapped Positive-ion mode is as follows: R^2^Y = 0.49, Q^2^Y = 0.42, and Q^2^*X*_0_ = −0.22. Negative-ion mode is as follows: R^2^Y = 0.36, Q^2^Y = 0.30, and Q^2^*X*_0_ = −0.18. Also, in the heatmap analysis (Figures 3(c) and 3(d)), the overall metabolic patterns of IBD and Con were clearly different in the negative-ion mode, but not in the positive-ion mode. Compared with Con, glycylglycine, (-)-3-(3,4-dihydroxyphenyl)-2-methylalanine, and guanosine 5′-diphosphate were increased (Figures 3(e) and 3(f)). Finally, we identified 20 differential metabolites (Supplementary Material 5 and Table S2).

To assess the diagnostic performance of plasma differential metabolites, we performed ROC curve analysis. In plasma samples, 29 of 57 differential metabolites have AUC ≥ 0.8 (Supplementary Material 5 and Table S3). In urine samples, 11 differential metabolites have AUC ≥ 0.8 (Supplementary Material 5 and Table S3). Finally, we found 11 potential diagnostic biomarkers with AUC ≥ 0.85 and FC ≥ 2 or ≤ 0.3 in plasma or urine samples (Table 2).

### 3.3. Metabolomic Profile of IBD-DH

**Figure 4:**
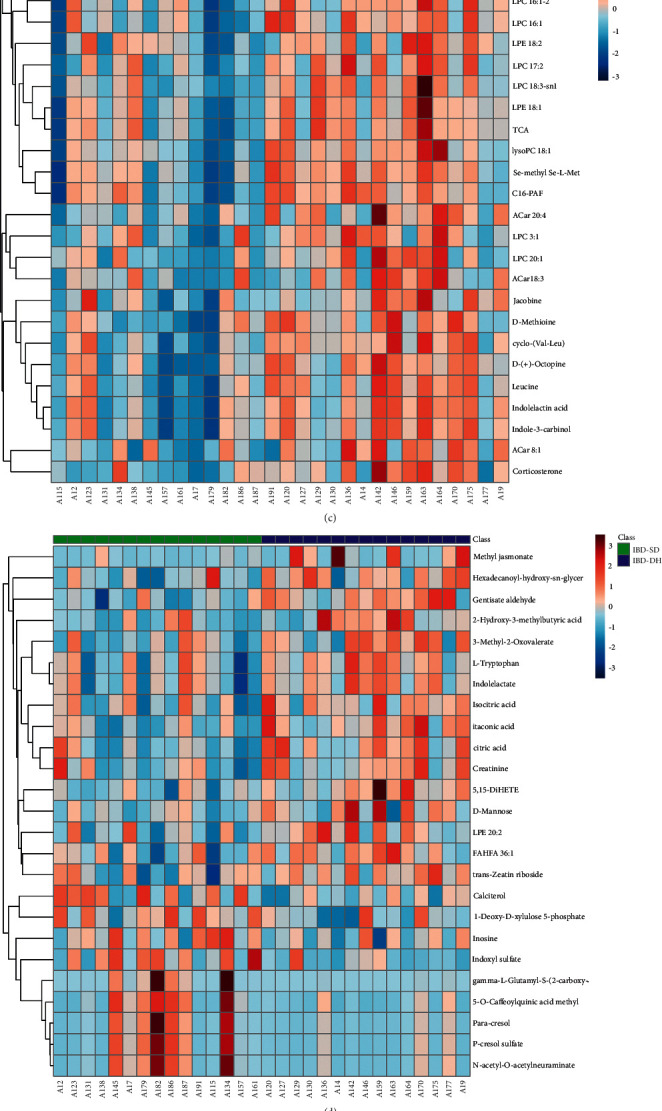
(a), (b) Plasma PLS-DA scores scatter plot (green: inflammatory bowel disease with damp-heat syndrome; blue: inflammatory bowel disease with spleen deficiency syndrome). (c), (d) Plasma heatmap analysis (green: inflammatory bowel disease with spleen deficiency syndrome; blue: inflammatory bowel disease with damp-heat syndrome). (e), (f) Volcanic map analysis (blue dot represents metabolite of downregulation, and red dot represents metabolite of upregulation). (a), (c), and (e) Positive-ion mode. (b), (d), and (f) Negative-ion mode.

In plasma samples, PLS-DA models showed that there is a smaller difference in plasma metabolites of IBD-DH as a subtype of IBD when compared with IBD-SD. Positive-ion mode is as follows: R^2^Y = 0.46, Q^2^Y = 0.35, and Q^2^*X*_0_ = −0.38. Negative-ion mode is as follows: R^2^Y = 0.73, Q^2^Y = 0.62, and Q^2^*X*_0_ = −0.43. In the heatmap analysis, the overall differences in metabolic patterns of IBD-DH and IBD-SD were not very obvious. Compared with IBD-SD, upregulated metabolites are mainly in the form of glycerophospholipids (LPC 17:2, LPC 18:3-sn1, and LPE 19:2) (Figure 4). According to the literature [25] and combined with our actual study, the threshold value of FDR was set to <0.2 in this section. With the conditions of FC > 1.5 or <2/3, VIP > 1.0, and FDR < 0.2, 16 differential metabolites were identified (Supplementary Material 5, and Table S4). There are 8 glycerophospholipids (LPC 20:1, LPC 17:2, LPC 18:3-sn1, LPC 16:0–2, LPC 18:1, LPE 19:2, PE 34:3e, and PC 38:7-sn1) and 4 fatty acyls and host-symbiotic intestinal flora amino acid (L-tryptophan). Unfortunately, we did not find significant differential metabolites in urine samples.

With ROC curve analysis, 7 differential metabolites have AUC ≥ 0.8 (Table 3). Sensitivity and specificity of most of them were more than 70%. Although we did not succeed in finding potential diagnostic biomarkers in IBD-DH versus IBD-SD, these metabolites might distinguish IBD-DH and IBD-SD to some extent.

### 3.4. Perturbed Metabolic Pathway

**Figure 5:**
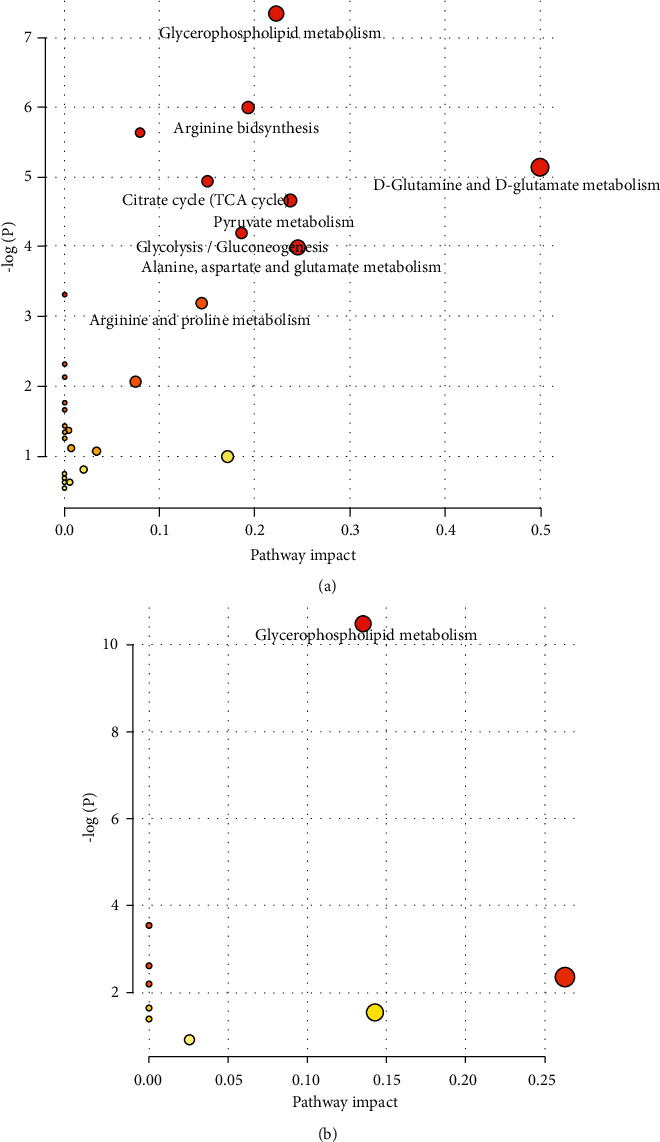
The *y*-axis is the negative logarithm of the statistic *p*-value; the *x*-axis is the pathway impact value. The larger the circle is, the greater the number of metabolite enrichments is and the more important the metabolic pathway is. The redder the color is, the more important the metabolic pathway is; the more yellowish, the less important. (a) The metabolic pathway of inflammatory bowel disease compared to healthy controls. (b) The metabolic pathway of damp-heat syndrome compared to spleen deficiency syndrome in inflammatory bowel disease.

We used 57 plasma differential metabolites in IBD and 16 plasma differential metabolites in IBD-DH for metabolic pathway enrichment analysis, respectively. The top 8 dysregulation metabolic pathways were relevant to citrate cycle, pyruvate metabolism, glycolysis/gluconeogenesis, glycerophospholipid metabolism, arginine and proline metabolism, arginine biosynthesis, D-glutamine and D-glutamate metabolism, alanine, aspartate, and glutamate metabolism in IBD versus Con. Compared to IBD-SD patients, IBD-DH patients possessed remarkable dysregulation of glycerophospholipid metabolism (Figure 5).

## 4. Discussion

From our data, the metabolic profiles of IBD and IBD-DH were better represented by plasma than urine. Although the plasma metabolic pathways of glycerophospholipid disturbed are consistent in both IBD and IBD-DH, their types of glycerophospholipid metabolites differ from each other. In our IBD patients, PC and PE acted mainly as parts of differential glycerophospholipid metabolites when compared with Con. But LPCs, another kind of glycerophospholipid, were mainly reflected in IBD-DH when compared with IBD-SD. In addition, acylcarnitine can distinguish IBD and IBD-DH from healthy controls and IBD-SD very well.

Previous studies showed that lipid metabolites play pivotal roles in the inflammatory process [26, 27]. In our study, abnormalities in lipids metabolism mainly involve glycerophospholipid and fatty acyls. Acylcarnitine has a significant impact on human pathophysiology and other aspects, which can affect cardiac ischemic outcomes, insulin sensitivity, and inflammation [28]. Acylcarnitine can activate nuclear factor kappa-B pathway in RAW264.7 cell mode [29]. Acylcarnitine induces the mRNA expression and secretion of proinflammatory cytokines (cyclooxygenase-2, interleukin-6, interleukin-1*β*, and tumor necrosis factor-*α*) and phosphorylation of c-Jun N-terminal kinase and extracellular-signal-regulated kinases, common mediators of the inflammatory response in the same cell model [30]. The study further confirmed that acylcarnitine relies on myeloid differentiation factor 88-activated nuclear factor kappa-B and inflammatory signaling pathways [30]. These acylcarnitines-related inflammatory signaling pathways are classical inflammatory pathways in the inflammatory response, especially in IBD [31, 32]. Acylcarnitine may be involved in the IBD inflammatory response. In our data, 3 ACars (ACar 20:4, ACar 18:1, and ACar 20:3) with high AUC value (0.88, 0.87, and 0.88) were significantly increased in IBD plasma samples. Besides these, the liver can produce acylcarnitines, which are secreted into the blood and bile. Acylcarnitine not only regulates inflammatory signaling pathways but also provides energy to cells. Acylcarnitine from bile can be transported to the intestinal lumen and enter the mitochondria of the intestinal epithelium for oxidation to release energy, thus providing energy to the intestinal epithelium. Another study found that the fecal acylcarnitine content in patients with IBD and mice with experimental colitis tended to increase compared to the control group, and the reason may be related to intestinal inflammation that led to mitochondrial dysfunction in the apical domain of the surface epithelium that may reduce the consumption of fatty acids [33]. Elevated acylcarnitine may be a useful marker for patients with intestinal inflammation. Our study had framed these possible acylcarnitines. Interestingly, ACar 8:1 was significantly increased in IBD-DH when compared with IBD-SD. Its specific mechanism of action needs to be further investigated.

Glycerophospholipids are the most abundant phospholipids in the body and have multiple uses in the body's life activities, not only providing energy for the body but also playing an important role in processes such as cell signal recognition and protein recognition [34, 35]. The glycerophospholipid metabolism was disturbed in IBD patients and animals [36, 37]. Glycerophospholipids can be further divided into PC, PE, phosphatidylserine, and so on. Normal intestinal mucosa contains two major phospholipids, more than 45% of the total phospholipid content is PC, and 22∼27% is PE [38]. Our supplement documents showed that PE increased and PC decreased in IBD patients' plasma. Our data showed that, compared with IBD-SD, LPC 17:2, LPC 18:3-sn1, and LPC 16:0–2 were increased in IBD-DH. Also, the expression of LPC, including LPC 18:1, LPC 16:0, LPC 18:0, and LPC 20:4, was increased in the 2,4,6-trinitrobenzenesulfonic acid- (TNBS-) induced colitis rats [39]. However, different reports appeared. Daniluk U et al. found that LPC 16:0, LPC 18:0, and so on were downregulated in newly diagnosed pediatric patients with IBD [40]. LPC, a component of oxidized low-density lipoprotein, can damage the mucosal barrier due to its detergent properties [41]. LPC can induce the recruitment of monocytes and proinflammatory cytokine production and directly increase endothelial permeability, which can further exacerbate inflammatory tissue injury [41, 42]. These results indicate that LPC can destroy the intestinal barrier and play a role in the mucosal inflammation in IBD. There is a higher amount of LPCs to be candidates as differential metabolites in IBD-DH. We infer that damp-heat syndrome may exacerbate the inflammatory response of IBD patients through LPCs. A previous report indicated that damp-heat syndrome was closely related to proinflammatory cytokines and typical medication to clear damp-heat would significantly reduce the LPC [43, 44]. We suppose that LPCs might have the potential to become diagnostic biomarkers when the sample size increases in the future. Other lipid metabolites which related to inflammation, 8(R)-hydroxy-(5Z,9 E,11Z,14Z)-eicosatetraenoic acid, dehydroepiandrosterone sulfate and 5b-Cholestane-3a,7a,12a,24,25-pentol in IBD, and docosahexaenoic acid in IBD-DH, also have significant changes in our data [39, 45, 46]. From the perspective of traditional medicine, damp heat is considered to be related to lipids. This is fully consistent with our results.

Consistent with other studies [47], amino acid metabolic pathways were also perturbed in our IBD patients. Amino acids are the basic metabolites of almost all types of cells and play an important role in maintaining intestinal health. In IBD patients, amino acids have a prominent place in gut barrier and anti-inflammatory cytokines production. They are also related to tight junction proteins, such as zonula occludens 1, oxidative stress, apoptosis of enterocytes, and proinflammatory cytokines produced in the inflammation of the digestive tract [48]. In our study, there were significant differences between IBD patients and healthy controls in amino acid metabolism. They were mainly arginine biosynthesis, the D-glutamine and D-glutamate metabolism, and the alanine, aspartate, and glutamate metabolism. In addition, more significant abnormalities in aromatic amino acid metabolites appeared in IBD. We found two potential biomarkers of them. They were 3-indoleacetic acid (tryptophan metabolites) and hippuric acid (phenylalanine metabolites).

Tryptophan and phenylalanine are human-microbial cometabolites as key actors in IBD. Tryptophan is the unique protein amino acid bearing an indole ring. 95% of dietary tryptophan was metabolized through the kynurenine pathways, and 4%–6% was subjected to bacterial degradation in the gut lumen, primarily generating indoles [49]. 3-Indoleacetic acid is one of the indole metabolites of tryptophan. Indole propionic acid (IPA) is a metabolite produced exclusively by the microbiota from dietary tryptophan that accumulates in host serum [50]. IPA can be catalyzed to indoleacetic acid. Some researchers found that bacterial tryptophan metabolites such as IPA and 3-indoleacetic acid can activate aromatic hydrocarbon receptors or pregnane X receptor (PXR, an IBD therapeutic target) transcription factors to play an important protective and anti-inflammatory role in the gut by affecting interleukin-22 secretion [51]. This might be the reason for indoleacetic acid are high in the plasma of IBD patients. However, one Chinese research team demonstrated that four metabolites were elevated in the urine of the animals in the TNBS experimental colitis rats, two tryptophan metabolites (4-(2-aminophenyl)-2,4-dioxobutanoic acid and 4,6-cihydroxyquinoline) and another two gut microbial metabolites (phenylacetylglycine and p-cresol glucuronide) [52]. Increased tryptophan metabolism and tryptophan metabolites have been identified in a cohort of 535 patients with IBD [53]. Our study confirmed this finding in the metabolites of plasma. Indoleacetic acid was significantly increased in IBD patients. Tryptophan was even higher in IBD-DH as demonstrated in our data (Supplementary Material 5). This might indicate that damp-heat syndrome is strengthening the gut microbiota effects.

A decrease in the metabolites of another aromatic amino acid phenylalanine has been found in patients with IBD. A study has found increased fecal phenylalanine levels in patients with Crohn's disease due to reduced amino acid uptake caused by inflammation [54]. Elevated level of hippuric acid is a marker of metabolic health [55]. The discrepancy between hippuric acid and indoleacetic acid levels in plasma cannot be explained right now.

## 5. Conclusion

We used the pseudotargeted metabolomics method based on UHPLC-HRMS and TQMS to characterize the plasma and urine metabolism of IBD and its subtype IBD-DH. Urinary metabolites did not reflect the metabolic characteristics of IBD and IBD-DH very well. In plasma samples, eight metabolites that belong to aromatic acids, lipid derivatives were useful markers for diagnosing IBD. Glycerophospholipid metabolism perturbation is not only a manifestation of IBD but also an important way to distinguish two IBD subtypes defined by traditional medicine. Our work is a preliminary study, further large cohort studies are needed to confirm these findings, and multifactors predicting model is also worthy of being considered.

## Figures and Tables

**Table 1:** Clinical and demographic characteristics of participants.

	IBD (IBD-DH + IBD-SD)	Con	IBD-DH	IBD-SD
Number	30	15	15	15
Age, year (mean ± SD)	37.2 ± 10.6	38.8 ± 11.8	38.0 ± 11.5	36.5 ± 10.0
Sex (male/female)	14/16	7/8	7/8	7/8
BMI (mean ± SD)	21.0 ± 3.6	22.1 ± 2.9	21.8 ± 4.5	20.2 ± 2.1
Urine specific gravity (mean ± SD)	1.01 ± 0.01	1.01 ± 0.01	1.01 ± 0.02	1.02 ± 0.01
5-Aminosalicylic acid	26	-	13	13
Chinese medicine	4	-	2	2

IBD: inflammatory bowel disease; IBD-DH: IBD with damp-heat syndrome; IBD-SD: IBD with spleen deficiency syndrome; Con: healthy controls.

**Table 2:** Potential diagnostic biomarkers in IBD versus Con.

No.	Compounds	AUC	Sensitivity (%)	Specificity (%)	FC	log2 (FC)	*P*	FDR	VIP	Ion mode
P1	3-Indoleacetic acid	0.86	86.7	80.0	56.995	5.833	<0.001	0.001	1.898	Pos
P2	ACar 20:4	0.88	86.67	83.33	2.620	1.390	<0.001	<0.001	2.244	Pos
P3	ACar 18:1	0.87	80.0	80.0	2.375	1.248	<0.001	<0.001	2.340	Pos
P4	ACar 20:3	0.88	86.7	76.6	2.410	1.269	<0.001	<0.001	2.360	Pos
P5	Hippuric acid	0.88	80.0	93.3	0.281	−1.830	<0.001	0.002	2.166	Neg
P6	8(R)-Hydroxy-(5Z,9 E,11Z,14Z)-eicosatetraenoic acid	0.89	93.3	86.6	2.741	1.455	<0.001	<0.001	1.796	Neg
P7	Dehydroepiandrosterone sulfate	0.90	93.3	83.3	0.288	−1.796	<0.001	0.002	2.089	Neg
P8	5b-Cholestane-3a,7a,12a,24,25-pentol	0.93	80.0	93.3	0.248	−2.014	<0.001	0.006	2.116	Neg
U1	(-)-3-(3,4-Dihydroxyphenyl)-2-methylalanine	0.91	86.67	80	133.58	7.0615	<0.001	<0.001	1.740	Neg
U2	Glycylglycine	0.86	86.67	80	30.423	4.9271	<0.001	<0.001	1.784	Neg
U3	Guanosine 5′-diphosphate	0.88	80	90	46.046	5.525	<0.001	<0.001	1.909	Neg

**Table 3:** Plasma differential metabolites (AUC ≥ 0.8) in IBD-DH versus IBD-SD.

Compounds	Class	AUC	Sensitivity (%)	Specificity (%)	Trend
Cyclo-(Val-Leu)	Carboxylic acids and derivatives	0.81	80.00	73.33	↑
ACar 8:1	Fatty acyls	0.81	73.33	73.33	↑
LPC 17:2	Glycerophospholipids	0.80	66.67	86.67	↑
LPC 18:3-sn1	Glycerophospholipids	0.81	93.33	60.00	↑
LPC 16:0–2	Glycerophospholipids	0.80	73.33	80.00	↑
LPE 19:2	Glycerophospholipids	0.84	80.00	93.33	↑
Docosahexaenoic acid	Fatty acyls	0.80	66.67	93.33	↑

## Data Availability

The data supporting the findings of this study are available within the article and its supplementary materials.

## Abbreviations

ACar: Acylcarnitine
CA: Cholic acid
CDCA: Chenodeoxycholic acid
FFA: Free fatty acid
ICD: International Classification of Diseases
LPC: Lyso-phosphatidylcholine
LPE: Lyso-phosphatidylethanolamine
LPG: Lysyl-phosphatidylglycerol
PC: Phosphatidylcholine
PE: Phosphatidylethanolamine
Phe: Phenylalanine
SM: Sphingomyelin
TNBS: 2,4,6-Trinitrobenzenesulfonic acid
Trp: Tryptophan
IPA: Indole propionic acid.

## Supplementary Materials

Supplementary Materials Supplementary Material 1: damp-heat syndrome and spleen deficiency syndrome diagnosis standards and untargeted metabolomics based UHPLC-HRMS and multilevel metabolite characterization system. Supplementary Material 2: plasma ion-pairs information in positive mode and negative mode. Supplementary Material 3: pseudotargeted metabolomics method of urine samples. Supplementary Material 4: urine ion-pairs information in positive mode and negative mode. Supplementary Material 5: plasma and urine differential metabolites in IBD versus Con; plasma differential metabolites in IBD-DH versus IBD-SD.
