# Supplementary material for: Biomarkers of Metabolomics in Inflammatory Bowel Disease and Damp-Heat Syndrome: A Preliminary Study
Source: Evid Based Complement Alternat Med. 2022 Jul 1;2022:3319646. doi: 10.1155/2022/3319646 (PMC9270137; doi:10.1155/2022/3319646)
Supplement: Supplementary Materials — Supplementary Material 1: damp-heat syndrome and spleen deficiency syndrome diagnosis standards and untargeted metabolomics based UHPLC-HRMS and multilevel metabolite characterization system. Supplementary Material 2: plasma ion-pairs information in positive mode and negative mode. Supplementary Material 3: pseudotargeted metabolomics method of urine samples. Supplementary Material 4: urine ion-pairs information in positive mode and negative mode. Supplementary Material 5: plasma and urine differential metabolites in IBD versus Con; plasma differential metabolites in IBD-DH versus IBD-SD. [file 3319646.f1.zip › 3319646.f1/Supplementary material 1 (1).doc]

1. Diagnostic criteria for damp heat and spleen deficiency syndrome differentiation of IBD patients

Damp-heat syndrome:

Primary symptoms: (1) diarrhea, bloody stool with mucus and pus. (2) abdominal pain. (3) tenesmus.

Secondary symptoms: (1) burning sensation of anus. (2) abdominal distension. (3) dark urine with less quantity than normal. (4) dry Mouth. (5) bitter taste of mouth.

Tongue: red tongue, yellow and greasy tongue coating.

Pulse: slippery pulse.

Spleen deficiency syndrome:

Primary symptoms: (1) bloody purulent stool, more white（pus and mucus） and less red(blood), or just white (pus and mucus). (2) abdomen dull pain. (3) diarrhea and loose stools with indigestible food. (4) epigastric fullness and abdominal distension.

Secondary symptoms: (1) weakness of four limbs. (2) eat less and poor appetite. (3) lethargy.

Tongue：pale tongue with tooth prints on the edge, thin and white tongue coating or greasy tongue coating.

Pulse: thready and weak pulse, or thready and slippery pulse.

Syndrome diagnosis: two primary symptoms and two secondary symptoms. Diagnosis can be made by referring to tongue and pulse at same time.

2. Untargeted metabolomics based ultra-high-performance liquid chromatography-high-resolution mass spectrometry (UHPLC-HRMS)

UHPLC-HRMS analysis was performed on Q-Exactive mass spectrometry(Thermo, USA) coupled with 30000 liquid chromatography system(Thermo, USA), Xcalibur data acquisition software( version 4.0, Thermo, USA).

Chromatographic conditions

Positive ion mode: Chromatographic column: BEH C8 column (1.7 µm, 2.1×100 mm) Waters Corporation, USA; column temperature: 50°C; injection volume: 5 µL; flow rate: 0.35 mL/min; mobile phase A: 0.1% formic acid/water; mobile phase B: 0.1% formic acid/acetonitrile; gradient elution procedure: 5% of phase B as the starting concentration, 0-1 min; phase B changes from 5% to 100%, 1.1-11 min; phase B remains at 100%, 11.1-13 min; phase B remains at 5%,13.1-15 min.

Negative ion mode: Chromatographic column: HSS T3 column (1.8 µm, 2.1×100 mm) Waters Corporation, USA; column temperature: 50°C; injection volume: 5 µL; flow rate: 0.35 mL/min; mobile phase A: 0.1% formic acid/water; mobile phase B: 0.1% formic acid/acetonitrile; gradient elution procedure: 5% of phase B as the starting concentration, 0-1 min; phase B changes from 5% to 100%, 1.1-11 min; phase B remains at 100%, 11.1-13 min; phase B remains at 5%,13.1-15 min.

Mass spectrometry conditions

Positive ion mode: Mass spectrometry primary full scan +data dependent acquisition (DDA) secondary ion scan

The ESI-Positive mode of the heated electrospray ion source is used, and the primary full scan + DDA secondary ion scan mode is used. Spray Voltage : +3.8 kV; Capillary temperature: 320℃; Aux gas heater temperature: 350℃; Sheath gas flow rate: 35Arb; Aux gas flow rate: 8 Arb; S-lens RF level: 50; Mass range (*m/z*): 90-1050, Full MS resolution: 70000; MS/MS resolution: 17500; Top N: 5; Normalized collision energy(NCE)/stepped NCE: 20,40.

Negative ion mode: Mass spectrometry primary full scan + DDA secondary ion scan

Spray Voltage: -3.0kV; Capillary temperature: 320℃; Aux gas heater temperature: 350℃; Sheath gas flow rate: 35Arb; Aux gas flow rate: 8 Arb; S-lens RF level: 50; Mass range (*m/z*): 70-1050, Full MS resolution: 70000; MS/MS resolution: 17500; Top N: 5; NCE/stepped NCE: 20,40.

3. Multi-level metabolite characterization system

(1) The first level of database-qualitative annotation based on standard compounds

The data in the database of standard compounds are collected by the Metabolomics Research Center of Dalian Institute of Chemical Physics, Chinese Academy of Sciences after the purchase of standards, and the retention time, primary mass spectra and secondary mass spectra information are collected in positive and negative ion mode at high, medium and low energies, respectively. This information is richer and more complete, so that accurate qualitative analysis results can be obtained in the qualitative analysis. All data acquisition is based on SOP methods, and we have added internal standards in the data acquisition of standards. In the experimental analysis of this project, the same internal standard was added to correct the retention time, thus obtaining qualitative results with high accuracy and reliability. The standards database contains secondary mass spectrometry data for 7,200 standards.

(2) Second level of database-qualitative annotation based on Kyoto Encyclopedia of Genes and Genomes(KEGG) targeting database

Based on the KEGG targeting database, we constructed personalized databases with relatively strong qualitative capabilities based on different sample types and metabolite types. We systematically constructed primary and secondary mass spectrometry databases for each metabolite in KEGG, partially containing retention time information. Using the data from the KEGG database for characterization has a higher accuracy. For example, if only primary mass spectra are used for characterization, the number of candidate compounds is very large and the accuracy is very low when using general public data such as Human Metabolome Database for primary mass spectra, while the reliability is significantly higher when using the KEGG database for targeted characterization.

(3) Third level of database- qualitative annotation based on a multi-source hybrid database

Multi-source hybrid databases (e.g, Pubchem database) integrate retention time, primary mass spectrometry and secondary mass spectrometry data of metabolites from massive literature resources. The metabolites in these data, with varying degrees of information richness, contain different instrumentation and experimental conditions, as well as other factors that enhance the information richness of metabolite data. The advantage of hybrid databases, despite the mixed information, is the systematic global characterization of characterized metabolites obtained from different dimensions. Intuitively, these data under rich conditions obtain the full picture of characterized metabolite secondary mass spectrometry features as much as possible, and it is entirely possible to perform effective qualitative analysis of unknown metabolite features obtained under different conditions using the algorithm of forward search during qualitative search, and the powerful qualitative analysis capability of this database is well verified by a large number of actual data test analyses. At present, this database contains more than 2 million secondary mass spectrometry information, which is very powerful for the qualitative analysis of unknown metabolites.
