# Supplementary material for: Biomarkers of Metabolomics in Inflammatory Bowel Disease and Damp-Heat Syndrome: A Preliminary Study
Source: Evid Based Complement Alternat Med. 2022 Jul 1;2022:3319646. doi: 10.1155/2022/3319646 (PMC9270137; doi:10.1155/2022/3319646)
Supplement: Supplementary Materials — Supplementary Material 1: damp-heat syndrome and spleen deficiency syndrome diagnosis standards and untargeted metabolomics based UHPLC-HRMS and multilevel metabolite characterization system. Supplementary Material 2: plasma ion-pairs information in positive mode and negative mode. Supplementary Material 3: pseudotargeted metabolomics method of urine samples. Supplementary Material 4: urine ion-pairs information in positive mode and negative mode. Supplementary Material 5: plasma and urine differential metabolites in IBD versus Con; plasma differential metabolites in IBD-DH versus IBD-SD. [file 3319646.f1.zip › 3319646.f1/Supplementary material 2 (2).doc]

1 Plasma positive ions

| Name | Q1(Da) | Q3(Da) | Expected RT (min) | CE (eV) | DP (eV) |
| --- | --- | --- | --- | --- | --- |
| 2-Aminooctanoic acid | 160.1 | 101.1 | 0.8 | 15 | 80 |
| Atenolol | 267.2 | 145.1 | 0.6 | 30 | 80 |
| betazole | 109.1 | 70.1 | 0.6 | 15 | 80 |
| L-Carnitine | 310.3 | 97.1 | 0.6 | 15 | 80 |
| Pyrrolidine | 72.1 | 55.1 | 0.9 | 15 | 80 |
| Piperidine | 86.1 | 69.1 | 1.2 | 15 | 80 |
| Arginine | 175 | 158 | 0.7 | 20 | 100 |
| Glycohyocholic acid | 466.3 | 430.3 | 0.8 | 15 | 80 |
| Choline | 104.1 | 60.1 | 0.8 | 30 | 80 |
| DMG | 104 | 58 | 0.8 | 20 | 90 |
| Pro-Leu | 229.2 | 142.1 | 0.8 | 30 | 80 |
| Histamine | 213.2 | 149.9 | 0.8 | 40 | 80 |
| Creatinine | 114 | 44 | 0.8 | 20 | 100 |
| Diisooctyl phthalate | 413.3 | 301.1 | 0.9 | 30 | 80 |
| Uric acid | 169 | 141 | 0.9 | 15 | 80 |
| 3-methylolphenol | 107 | 93.1 | 1 | 15 | 80 |
| 1-Methyladenosine | 282.1 | 150.1 | 1.3 | 30 | 80 |
| D-Methionine | 150.1 | 61 | 1 | 30 | 80 |
| lenticin | 247.1 | 188.1 | 1 | 15 | 80 |
| dimethyl sulfone | 95 | 63 | 0.6 | 15 | 90 |
| β-Nicotyrine | 159.1 | 132.1 | 0.6 | 15 | 80 |
| D-Pipecolinic acid | 130.1 | 84.1 | 1 | 30 | 80 |
| Hypotaurine | 110 | 95.1 | 1 | 40 | 80 |
| Orthocumarsaure | 165.1 | 147 | 1 | 15 | 80 |
| 1,3-Dihydroxyacetone | 91.1 | 65 | 1.1 | 30 | 80 |
| L-Tryptophan | 205.1 | 188.1 | 1.1 | 15 | 80 |
| Protopine | 354.1 | 253.1 | 1.1 | 30 | 80 |
| Trigonelline | 138.1 | 110.1 | 0.6 | 15 | 80 |
| Dimedazol | 147.1 | 129.1 | 1.1 | 15 | 80 |
| Ormosanine | 318.3 | 256.3 | 1.1 | 30 | 80 |
| Pirimicarb | 239.1 | 133.1 | 1.1 | 15 | 80 |
| Ribitol | 153.1 | 136 | 1.1 | 15 | 80 |
| Serotonin | 177.1 | 80.1 | 1.1 | 30 | 80 |
| Acetaminophen D-glc | 328.1 | 152.1 | 1.1 | 15 | 80 |
| LPC 3:1 | 312.1 | 132.1 | 1.3 | 15 | 80 |
| Phthalic acid | 167 | 149 | 1.3 | 15 | 80 |
| Seroquel | 384.2 | 253.1 | 1.4 | 30 | 80 |
| Neocuproine | 209.1 | 153.1 | 1.4 | 15 | 80 |
| ACar 3:0 | 218.1 | 85 | 1.6 | 30 | 80 |
| Hirsuteine | 367.2 | 326.4 | 1.8 | 15 | 80 |
| C18796 | 100.1 | 82.1 | 1.8 | 30 | 80 |
| Valerolactam | 100.1 | 56.1 | 1.8 | 15 | 80 |
| L-Phenylalanine-d5 | 171.1 | 154.1 | 1.9 | 15 | 80 |
| Dimethylguanosine | 312.1 | 180.1 | 1.9 | 15 | 80 |
| Indoline | 120.1 | 103.1 | 1.9 | 15 | 80 |
| L-Phenylalanine | 166.1 | 120.1 | 1.9 | 15 | 80 |
| S-Acetylcysteamin | 120 | 104.1 | 1.9 | 15 | 80 |
| Tyramine | 121.1 | 77 | 1.9 | 30 | 80 |
| AOZ | 103.1 | 77 | 1.9 | 30 | 80 |
| Cinnamic acid | 131 | 103.1 | 1.9 | 15 | 80 |
| LPE 27:0 | 608.5 | 184.1 | 2 | 30 | 80 |
| 4-Hydroxyquinoline | 146.1 | 129.1 | 2 | 15 | 80 |
| Indolelactic acid | 206.1 | 130.1 | 2 | 45 | 80 |
| 4-Acetamidophenol | 152.1 | 110.1 | 2.1 | 15 | 80 |
| 1,7-Dimethylxanthine | 181.1 | 124.1 | 2.6 | 30 | 80 |
| Acisoga | 185.1 | 143.1 | 2.6 | 30 | 80 |
| MLS000061860 | 280.2 | 85 | 2.6 | 30 | 80 |
| DEPA | 192.1 | 149 | 2.9 | 30 | 80 |
| Kynurenine | 209.1 | 192.1 | 2.9 | 15 | 80 |
| 3-Methyladenine | 150.1 | 122.1 | 2.9 | 15 | 80 |
| Semiamitraz | 163.1 | 135.1 | 2.9 | 30 | 80 |
| Guanidinoacetate | 162.1 | 118 | 2.9 | 30 | 80 |
| linolenic acid | 279.2 | 149 | 2.9 | 15 | 80 |
| N-Phenethylacetamide | 163.1 | 161.1 | 2.9 | 30 | 80 |
| (R)-Mevalonate | 149.1 | 130 | 2.9 | 15 | 80 |
| 2-(Octylthio)ethanol | 191.1 | 173.1 | 2.9 | 30 | 80 |
| 2,6-Dimethoxyquinone | 149.1 | 132 | 2.9 | 30 | 80 |
| Actinidine | 148.1 | 146.1 | 2.9 | 30 | 80 |
| Carvyl propionate | 212 | 194 | 2.9 | 15 | 80 |
| GPC | 258.2 | 104 | 2.9 | 25 | 90 |
| Jacobine | 352.2 | 188.1 | 2.9 | 30 | 80 |
| L-Tyrosine | 182.1 | 165.1 | 2.9 | 15 | 80 |
| N-Acetyl-L-tyrosine | 223.1 | 181 | 2.9 | 40 | 100 |
| N-Acetylmethionine | 192.1 | 149.9 | 2.9 | 15 | 80 |
| Tromethamine | 122.1 | 120.1 | 2.9 | 15 | 80 |
| Tryptophan-d5 | 210.1 | 192.1 | 2.9 | 15 | 80 |
| 3-Methylquinoline | 144.1 | 127.1 | 2.9 | 15 | 80 |
| cyclo-(Val-Leu) | 118.1 | 79 | 2.9 | 40 | 80 |
| L-serine | 106 | 60 | 2.9 | 15 | 50 |
| D-(+)-Octopine | 247.1 | 189.1 | 3 | 30 | 80 |
| Leucine | 132.1 | 116 | 2.6 | 40 | 80 |
| Indole-3-carbinol | 130.4 | 103 | 3 | 27 | 140 |
| O-Butanoylcarnitine | 232.2 | 173.1 | 2.2 | 15 | 80 |
| ACar 4:0 | 232.2 | 85 | 2.2 | 30 | 80 |
| Piperine | 286.1 | 202.1 | 3.1 | 40 | 80 |
| 3-Indoleacetic acid | 176.1 | 132.1 | 3.2 | 15 | 80 |
| γ-Glu-Leu | 261.1 | 132.1 | 3.3 | 15 | 80 |
| Caffeine | 195.1 | 138.1 | 3.4 | 30 | 80 |
| Hexadecanamide | 256.3 | 88.1 | 3.5 | 30 | 80 |
| p-Formylphenol | 123 | 51 | 3.6 | 45 | 80 |
| PHE-AC-GLN-OH | 265.1 | 130.1 | 3.6 | 15 | 80 |
| PHE-AC-GLN-OH2 | 265.1 | 147.1 | 3.6 | 30 | 80 |
| Proline | 116.1 | 70.1 | 3.6 | 30 | 80 |
| ACar 5:1 | 244.2 | 85 | 3.1 | 30 | 80 |
| Hydrocortisone | 363.2 | 121.1 | 3.8 | 30 | 80 |
| 1,2-Diaminobenzene | 109.1 | 83.1 | 3.8 | 15 | 80 |
| 4-Methylhistamine | 126.1 | 124.1 | 3.8 | 15 | 80 |
| DHC | 388 | 89 | 3.8 | 20 | 90 |
| Ecgonine | 186.1 | 126.1 | 3.5 | 30 | 80 |
| Taurine | 126 | 44 | 3.8 | 20 | 90 |
| 2-Hydroxyquinoline | 146.1 | 128 | 4.3 | 30 | 80 |
| Aspidocarpine | 371.2 | 89.1 | 3.8 | 30 | 80 |
| ACar 5:0 | 246.2 | 85 | 3.4 | 30 | 80 |
| Methylbutyroyl-CAR | 246.2 | 187.1 | 3.4 | 15 | 80 |
| Piperidione | 170.1 | 135.1 | 4.1 | 15 | 80 |
| Phe-Phe | 313.2 | 120.1 | 4.2 | 30 | 80 |
| O-Acetylcarnitine | 204.1 | 149 | 3.6 | 40 | 80 |
| Midazolam | 326.1 | 207.1 | 4.3 | 30 | 80 |
| Exemestane | 319.2 | 130.1 | 4.5 | 30 | 80 |
| 5-Hydroxyindole | 134.1 | 77 | 3.8 | 30 | 80 |
| O-Methylsinapic acid | 239.1 | 221.1 | 3.9 | 15 | 80 |
| D-Pantothenic acid | 220.1 | 178.2 | 4.8 | 15 | 80 |
| Indole | 118.1 | 91.1 | 4.5 | 30 | 80 |
| Anteisomargaric acid | 271.3 | 83.1 | 4.9 | 30 | 80 |
| Phe-Pro | 263.1 | 70.1 | 5 | 45 | 80 |
| ACar 6:0 | 260.2 | 85 | 4.2 | 30 | 80 |
| (±)-Hexanoylcarnitine | 260.2 | 201.1 | 4.2 | 30 | 80 |
| 3-Indolepropionic acid | 190.1 | 130.1 | 5.4 | 30 | 80 |
| Homogentisate | 169 | 148 | 5.5 | 40 | 80 |
| 3,4-dimethoxyflavonol | 299.1 | 211.1 | 5.3 | 45 | 80 |
| Tabersonine | 337.2 | 293.2 | 5.8 | 45 | 80 |
| ACar 14:2 | 368.3 | 85 | 6.7 | 30 | 80 |
| ACar 8:1 | 286.2 | 85 | 5.1 | 30 | 80 |
| MG 16:0 | 331.3 | 313.3 | 5.1 | 15 | 80 |
| 12OH-JA | 227.1 | 180.2 | 6.1 | 15 | 80 |
| 9-Oxodecyl-acetamide | 214.2 | 161.1 | 6.1 | 30 | 80 |
| 4,7-Phenanthroline | 181.1 | 163.1 | 6.3 | 15 | 80 |
| Diethyl-phthalate | 223.1 | 181.1 | 6.3 | 15 | 80 |
| Monolinolein | 355.3 | 263.2 | 7.1 | 15 | 80 |
| GCA | 488.3 | 470 | 6.4 | 40 | 100 |
| Glutamine | 147.1 | 130.1 | 6.4 | 15 | 80 |
| Glycodeoxycholic acid | 450.3 | 414.3 | 7.2 | 15 | 80 |
| L-kynurenine | 192.2 | 146 | 6.4 | 20 | 130 |
| ACN-OH | 310.2 | 85 | 6.5 | 40 | 100 |
| DG 16:0_18:1 | 617.5 | 599.5 | 6.5 | 15 | 80 |
| Compand 1 | 211.2 | 109.1 | 6.6 | 15 | 80 |
| CCG-48252 | 290.2 | 85 | 6.7 | 30 | 80 |
| ACar 8:0 | 288.2 | 85 | 6.7 | 30 | 80 |
| cannitine C8:0-d3 | 291.2 | 86 | 6.7 | 15 | 80 |
| Androsterone | 291.2 | 229.1 | 6.7 | 30 | 80 |
| LPC 20:1 | 550.4 | 184.1 | 6.7 | 30 | 80 |
| Acar 9:1 | 300.2 | 85 | 6.3 | 30 | 80 |
| ACar 10:2 | 312.2 | 85 | 7.5 | 30 | 80 |
| ACar 16:4 | 392.3 | 85 | 7 | 30 | 80 |
| Cholic Acid | 426.3 | 355.3 | 7 | 30 | 80 |
| Docosanamide | 340.4 | 175.1 | 7.1 | 40 | 80 |
| GCDCA | 472.3 | 454 | 8 | 40 | 100 |
| Glycocholic acid | 448.3 | 309.3 | 6.5 | 30 | 80 |
| LPC 12:0 | 440.3 | 184 | 7.1 | 30 | 100 |
| LPE 16:1 | 452.3 | 311 | 8 | 40 | 100 |
| Diisopropylphthalate | 251.1 | 209.1 | 7.3 | 15 | 80 |
| LPC 22:1 | 578.4 | 184.1 | 6.6 | 45 | 80 |
| Biliverdin | 583.3 | 297.1 | 6.8 | 45 | 80 |
| 11-Eicosenoic acid | 311.3 | 293.3 | 7.4 | 15 | 80 |
| ACar 12:1 | 342.3 | 85 | 7.4 | 30 | 80 |
| Cer-NS d40:1 | 622.6 | 604.6 | 8 | 15 | 80 |
| Cer-NS d42:3 | 646.6 | 628.6 | 7.1 | 15 | 80 |
| ACar 9:0 | 302.2 | 85 | 6.9 | 30 | 80 |
| ACar 13:1 | 356.3 | 85 | 7.7 | 30 | 80 |
| ACar 16:3 | 394.3 | 85 | 7 | 30 | 80 |
| ACar 14:1 | 370.3 | 85 | 8.2 | 30 | 80 |
| Acar 10:1 | 314.2 | 85 | 7.7 | 30 | 80 |
| Isobutyl phosphate | 267.2 | 99 | 7.1 | 30 | 80 |
| Isolithocholate | 377.3 | 360.3 | 7.7 | 30 | 80 |
| Acetylepisamarcandin | 460.3 | 135.1 | 7.7 | 30 | 80 |
| LPE 18:2 | 478.3 | 337 | 8.4 | 40 | 100 |
| Dihydrosphingosine | 302.3 | 284.3 | 6.9 | 30 | 80 |
| LPC 14:0 | 468.3 | 184 | 7.9 | 40 | 100 |
| Salicylic acid | 121 | 65 | 7.8 | 30 | 80 |
| LPC 12:0-2 | 440.3 | 184.1 | 7.2 | 30 | 80 |
| ACar 20:4 | 448.3 | 85 | 7.6 | 30 | 80 |
| Aldosterone | 361.2 | 163 | 7.9 | 40 | 100 |
| Norfluoxetine | 296.1 | 210 | 7.9 | 30 | 80 |
| Erucic acid | 361.3 | 361.3 | 7.9 | 15 | 80 |
| 18-Hydroxyoleate | 437.2 | 437.2 | 7.8 | 15 | 80 |
| δ4-Dafachronic acid | 415.3 | 397.3 | 7.8 | 15 | 80 |
| LPC 18:3 | 518.3 | 459 | 8.6 | 40 | 100 |
| LPC 17:2 | 506.3 | 184.1 | 8.1 | 30 | 80 |
| LPE 16:0-2 | 454.3 | 313.3 | 8.6 | 30 | 80 |
| LPC 16:0 | 496.3 | 184 | 8.5 | 40 | 100 |
| LPC 16:1 | 494.3 | 184 | 8.1 | 40 | 100 |
| SM 34:2 | 701.6 | 184.1 | 8.2 | 30 | 80 |
| LPC 15:0 | 482.3 | 184 | 8.2 | 40 | 100 |
| LPC 14:0-2 | 468.3 | 184.1 | 7.9 | 30 | 80 |
| PC 16:0_22:1 | 816.7 | 184.1 | 8.5 | 45 | 80 |
| DTXSID40893993 | 398.2 | 253.1 | 8.2 | 30 | 80 |
| LPC 18:2-2 | 520.3 | 503.3 | 8.2 | 40 | 80 |
| LPE 22:4-2 | 530.3 | 471 | 9 | 40 | 100 |
| LPC 20:4-sn2 | 544.3 | 522.4 | 8.9 | 15 | 80 |
| Dihydrocoumarin | 542.3 | 483.2 | 8.4 | 30 | 80 |
| LPE 18:1-3 | 502.3 | 361.3 | 8.3 | 15 | 80 |
| LPC 18:1 | 522.4 | 184 | 8.4 | 40 | 100 |
| LPC 22:5-2 | 570.4 | 184.1 | 8.6 | 30 | 80 |
| Oleamide-2 | 282.3 | 265.3 | 8.4 | 15 | 80 |
| SM d33:2 | 687.5 | 184.1 | 9 | 45 | 80 |
| LPC 20:3-4 | 546.4 | 104.1 | 8.7 | 40 | 80 |
| LPC O-18:0-2 | 510.4 | 184.1 | 9 | 30 | 80 |
| ACar 10:0 | 316.2 | 85 | 8.6 | 30 | 80 |
| ACar 10:0-d3 | 319.3 | 85 | 8.5 | 30 | 80 |
| ACar 16:2 | 396.3 | 85 | 8.5 | 30 | 80 |
| LPE 16:0 | 454.3 | 313 | 8.5 | 40 | 100 |
| LPE 12:0 | 398.2 | 149 | 8.5 | 30 | 80 |
| LPC P-18:0 | 508.4 | 104 | 9 | 40 | 100 |
| Sugeonol | 235.2 | 57.1 | 8.5 | 15 | 80 |
| LPC 18:2-sn2 | 520.3 | 184.1 | 8.5 | 30 | 80 |
| LPC 18:3-sn1 | 518.3 | 184.1 | 8.1 | 30 | 80 |
| LPC 22:5 | 570.4 | 184 | 8.6 | 40 | 100 |
| LPC 22:4 | 572.4 | 522.4 | 8.6 | 15 | 80 |
| LPC 16:0-2 | 496.3 | 185.1 | 8.6 | 40 | 80 |
| Irgarol | 254.1 | 219.1 | 8.6 | 15 | 80 |
| LPC 15:0-2 | 482.3 | 184.1 | 8.2 | 30 | 80 |
| LPC 20:5 | 542.3 | 483 | 8.4 | 40 | 100 |
| lysoPC 16:1 | 494.3 | 185.1 | 7.9 | 40 | 80 |
| LPE 20:5 | 500.3 | 457 | 8.4 | 40 | 100 |
| LPE 18:1 | 480.3 | 339 | 8.8 | 40 | 100 |
| TCA | 480.3 | 462 | 8.8 | 20 | 100 |
| LPE 18:0-2 | 482.3 | 341.3 | 9.2 | 30 | 80 |
| LMST03020032 | 391.3 | 374.3 | 6.5 | 40 | 80 |
| LMST03020417 | 429.4 | 165.1 | 11.1 | 15 | 80 |
| C16-PAF | 524.4 | 185.1 | 8.8 | 40 | 80 |
| LPC 20:4-sn1 | 544.3 | 184.1 | 8.5 | 30 | 80 |
| lysoPC 18:1 | 522.4 | 184.1 | 8.8 | 15 | 80 |
| 5-Acetoxypalisadin-B | 383.3 | 271.2 | 8.9 | 15 | 80 |
| Sclareol | 331.3 | 247.2 | 10.2 | 30 | 80 |
| Terfenadine | 472.3 | 428.4 | 10.8 | 30 | 80 |
| Se-methyl Se-L-Met | 522.4 | 504.3 | 8.8 | 15 | 80 |
| Stigmastentriol | 447.4 | 165.1 | 8.9 | 15 | 80 |
| LPE 16:1-2 | 452.3 | 434.3 | 8.1 | 15 | 80 |
| LPC O-16:0 | 482.4 | 104 | 8.8 | 40 | 100 |
| LPC 16:1-2 | 494.3 | 184.1 | 8.1 | 30 | 80 |
| LPC O-18:0 | 510.4 | 104 | 8.9 | 40 | 100 |
| LPC O-18:1 | 508.3 | 184.1 | 8.5 | 30 | 80 |
| LPE 22:4 | 528.3 | 387.3 | 8.8 | 30 | 80 |
| Phe-Trp | 352.2 | 120 | 4.3 | 40 | 100 |
| Corticosterone | 347.2 | 329.2 | 9.1 | 15 | 80 |
| Autumnolide | 281.1 | 150 | 9.1 | 30 | 80 |
| DOMA | 149 | 65 | 9.1 | 30 | 80 |
| LPC P-18:1-sn1 | 506.4 | 104.1 | 8.6 | 30 | 80 |
| Benzoylecgonine | 290.1 | 105 | 8.4 | 45 | 80 |
| LPC 17:0 | 510.4 | 184.1 | 8.9 | 30 | 80 |
| β-Linoleic acid | 281.2 | 149 | 9.1 | 15 | 80 |
| Dolichoic acid-[18-20] | 375.3 | 375.3 | 9.1 | 15 | 80 |
| LPC 19:0 | 538.4 | 184.1 | 9.6 | 30 | 80 |
| SM d32:2 | 673.5 | 184.1 | 9.1 | 30 | 80 |
| ACar 14:0 | 372.3 | 85 | 7.2 | 30 | 80 |
| ACar 15:0 | 386.3 | 85 | 6.4 | 30 | 80 |
| LPE 17:0 | 468.3 | 327.3 | 8.9 | 30 | 80 |
| Abietic acid | 303.2 | 285.2 | 9.3 | 15 | 80 |
| ACar 16:0 | 400.3 | 85 | 7.7 | 15 | 80 |
| LPE 18:0 | 482.3 | 341 | 9.2 | 40 | 100 |
| LPE 19:2 | 492.3 | 184.1 | 7.8 | 30 | 80 |
| LPC 18:0 | 524.4 | 184 | 9.3 | 40 | 100 |
| LPC 18:0-2 | 524.4 | 506.4 | 9.3 | 30 | 80 |
| PE 34:3e | 700.5 | 337.3 | 10.1 | 30 | 80 |
| Satratoxin G | 567.2 | 465.2 | 7.9 | 30 | 80 |
| SM 36:3 | 727.6 | 184 | 11.4 | 30 | 100 |
| SM 36:3 | 727.6 | 184.1 | 11.4 | 30 | 80 |
| ACar 18:2 | 424.3 | 85 | 7.5 | 45 | 80 |
| LPC 20:0 | 552.4 | 184.1 | 9.2 | 30 | 80 |
| SM 36:2 | 466.4 | 294 | 9.5 | 40 | 100 |
| Palmitoleic acid | 255.2 | 237.2 | 9.9 | 15 | 80 |
| Cer-NS d42:1-sn2 | 650.6 | 632.6 | 8.5 | 15 | 80 |
| LPE 22:6 | 526.3 | 385.3 | 8.4 | 30 | 80 |
| ACar 16:1 | 398.3 | 85 | 7.4 | 30 | 80 |
| ACar 26:1 | 538.5 | 85 | 9.3 | 45 | 80 |
| Cer d18:1/16:0 | 538.5 | 520.5 | 9.6 | 30 | 80 |
| ACar 18:1 | 426.4 | 85 | 7.9 | 30 | 80 |
| PC 19:0_20:4 | 824.6 | 184.1 | 8.6 | 45 | 80 |
| dibutyladipate | 259.2 | 185.1 | 9.7 | 15 | 80 |
| Hippuric acid | 180.1 | 105 | 3.6 | 15 | 80 |
| PC 24:0 | 622.4 | 184.1 | 9.6 | 30 | 80 |
| SM 35:2 | 715.6 | 184.1 | 11.5 | 30 | 80 |
| 11β-Misoprostol | 274.3 | 256.3 | 6.3 | 30 | 80 |
| ACar 12:0 | 344.3 | 85 | 6.7 | 30 | 80 |
| Monocrotaline | 342.2 | 110.1 | 1.4 | 30 | 80 |
| PC 18:0_20:3 | 829.7 | 184.1 | 6.6 | 30 | 80 |
| ACar 20:3 | 450.4 | 85 | 7.7 | 30 | 80 |
| ACar18:3 | 422.3 | 85 | 7.3 | 45 | 80 |
| PC 38:4 | 810.6 | 184 | 6.2 | 30 | 100 |
| Palmitic acid | 257.2 | 177.1 | 10.4 | 15 | 80 |
| M-DAP | 191.1 | 117.1 | 2.5 | 30 | 80 |
| LDAO | 230.2 | 62.1 | 6.4 | 15 | 80 |
| PE 36:4e | 726.5 | 337.3 | 10.1 | 30 | 80 |
| LPC 19:1 | 536.4 | 184.1 | 8.4 | 30 | 80 |
| Resibufogenin | 385.2 | 155.1 | 10.5 | 30 | 80 |
| Retinol | 269.2 | 121.1 | 10 | 15 | 80 |
| PC 18:0_22:6 | 834.6 | 184.1 | 12.2 | 45 | 80 |
| Oleamide | 282.3 | 97.1 | 10.1 | 15 | 80 |
| Docosahexaenoic acid | 329.2 | 311.2 | 9.2 | 15 | 80 |
| PC 16:0/22:6 | 806.6 | 185.1 | 11.8 | 40 | 80 |
| PC 18:0_20:4 | 832.6 | 184.1 | 11.9 | 45 | 80 |
| PE 16:0 _16:0 | 692.5 | 184.1 | 11 | 30 | 80 |
| PC 36:5 | 780.6 | 184 | 11.7 | 30 | 100 |
| Arachidonic acid | 305.2 | 287.2 | 10 | 15 | 80 |
| LPC 22:0 | 580.4 | 184.1 | 10.5 | 45 | 80 |
| PC 26:0 | 650.5 | 184.1 | 9.6 | 30 | 80 |
| PC O-38:4-sn1 | 796.6 | 184.1 | 8.4 | 45 | 80 |
| Farnesylacetone | 263.2 | 245.2 | 10.1 | 15 | 80 |
| Papaverine | 340.1 | 253.1 | 11.1 | 30 | 80 |
| PC 16:1_20:4 | 780.6 | 184.1 | 11.7 | 45 | 80 |
| PC 30:0e | 692.6 | 184.1 | 10.4 | 30 | 80 |
| ACar 20:1 | 454.4 | 85 | 8.6 | 45 | 80 |
| Tramadol | 264.2 | 58.1 | 10.1 | 30 | 80 |
| ACar 22:0 | 484.4 | 85 | 8.7 | 45 | 80 |
| DAG 17:1 | 374.3 | 357.3 | 10.1 | 15 | 80 |
| Deoxycholic acid | 357.3 | 339.3 | 9.9 | 15 | 80 |
| PC 38:5 | 808.6 | 184 | 11.7 | 30 | 100 |
| DGLA | 307.3 | 67.1 | 10.3 | 45 | 80 |
| Lyso-PAF C-16 | 209.2 | 191.1 | 5.6 | 30 | 80 |
| Osbond Acid | 331.3 | 313.3 | 10 | 15 | 80 |
| SM 30:1 | 647.5 | 185.1 | 10.2 | 30 | 80 |
| ACar 14:1-2 | 370.3 | 86 | 10.5 | 15 | 80 |
| ACar 26:0 | 540.5 | 85 | 10.1 | 45 | 80 |
| Remerine | 280.1 | 262.1 | 8.1 | 15 | 80 |
| 4-Cholesten-3-One | 385.3 | 109.1 | 10.5 | 30 | 80 |
| PC 34:4 | 754.5 | 184 | 11.6 | 30 | 100 |
| trans-Vaccenic acid | 283.3 | 265.3 | 10.6 | 15 | 80 |
| ACar 20:0 | 456.4 | 85 | 8.5 | 30 | 80 |
| PC 15:0_16:0 | 720.6 | 184.1 | 10.9 | 45 | 80 |
| PC 32:0-2 | 756.6 | 185.1 | 8.6 | 40 | 80 |
| PC 34:3 | 756.6 | 184.1 | 11.7 | 45 | 80 |
| PC 38:7 | 804.6 | 184 | 11.6 | 30 | 100 |
| PC 38:7-sn1 | 804.6 | 184.1 | 11.6 | 30 | 80 |
| FD C20:1 | 310.3 | 69 | 10.6 | 40 | 100 |
| PC 16:0_19:1 | 774.6 | 184.1 | 11.6 | 30 | 80 |
| PC 16:1/22:6 | 804.5 | 184.1 | 10.4 | 15 | 80 |
| PC O-36:6-sn2 | 764.6 | 184.1 | 11.9 | 45 | 80 |
| fatty amide C20:1 | 310.3 | 293.3 | 9.2 | 15 | 80 |
| Palmitoylpyrrolidine | 310.3 | 135.1 | 10.6 | 30 | 80 |
| Riboflavin | 377.1 | 243.1 | 7.1 | 30 | 80 |
| Palmitoyl-EA | 300.3 | 62.1 | 9.5 | 30 | 80 |
| PC 31:0 | 720.6 | 184 | 10.9 | 30 | 100 |
| LPC 20:5-3 | 542.3 | 520.3 | 8.4 | 15 | 80 |
| PE 36:5 | 738.5 | 597.5 | 11.7 | 30 | 80 |
| SM d31:1 | 661.5 | 184.1 | 10.9 | 30 | 80 |
| ACar 24:0 | 512.5 | 85 | 9.4 | 45 | 80 |
| PC O-36:2-sn1 | 772.6 | 184.1 | 8.6 | 30 | 80 |
| PC O-36:5-sn1 | 766.5 | 184.1 | 12.1 | 30 | 80 |
| PC O-38:2-sn1 | 817.7 | 184.1 | 10.4 | 30 | 80 |
| PC O-34:4-sn1 | 762.5 | 184.1 | 10.4 | 30 | 80 |
| 7-beta-OHC | 403.4 | 86 | 11.2 | 40 | 80 |
| FD C22:2 | 336.3 | 95 | 10.7 | 60 | 100 |
| fatty amide C22:2 | 336.3 | 319.3 | 10.7 | 15 | 80 |
| Laccer d18:1/16:0 | 862.6 | 844.6 | 6.6 | 15 | 80 |
| PC 33:2 | 744.6 | 184.1 | 11.8 | 45 | 80 |
| PC 32:1 | 732.6 | 184 | 11.9 | 30 | 100 |
| SM 30:0 | 649.5 | 184.1 | 10.6 | 30 | 80 |
| 7-Ketocholesterol | 401.3 | 383.3 | 10.8 | 30 | 80 |
| PC 39:2 | 828.6 | 184 | 6.5 | 30 | 100 |
| PC 18:0_20:5 | 808.6 | 184.1 | 11.9 | 30 | 80 |
| PC 33:3 | 742.5 | 184.1 | 12.2 | 30 | 80 |
| PE 34:4 | 712.5 | 571.5 | 9.3 | 30 | 80 |
| PC 18:1_18:2 | 784.6 | 184.1 | 12.1 | 30 | 80 |
| PC O-32:1-sn1 | 740.6 | 184.1 | 11.7 | 45 | 80 |
| PC O-36:5-sn2 | 766.5 | 184.1 | 12.1 | 30 | 80 |
| SM d31:2 | 659.5 | 184.1 | 10.5 | 30 | 80 |
| PC 36:4-3 | 782.6 | 185.1 | 11.7 | 30 | 80 |
| PC 36:4-2 | 782.6 | 184.1 | 11.8 | 15 | 80 |
| FD C20:0 | 312.3 | 74 | 11 | 60 | 100 |
| PC 33:1 | 746.6 | 184.1 | 9.7 | 45 | 80 |
| Cer-NS d42:1-sn1 | 650.6 | 264.3 | 10.4 | 45 | 80 |
| Eicosapentaenoic acid | 303.2 | 247.2 | 9.8 | 30 | 80 |
| Erucamide+ | 338.3 | 321.3 | 11.1 | 15 | 80 |
| Albuterol | 240.2 | 205.1 | 6.5 | 15 | 80 |
| 1921AH | 338.3 | 97.1 | 11.1 | 30 | 80 |
| FFA C22:1 | 338.3 | 321.3 | 11.1 | 15 | 80 |
| Erucamide | 338.3 | 83.1 | 11.1 | 30 | 80 |
| SM d34:1 | 568.3 | 184.1 | 8.4 | 40 | 80 |
| Stearamide | 284.3 | 88.1 | 10.4 | 30 | 80 |
| PC 36:3 | 784.6 | 184.1 | 12.1 | 45 | 80 |
| PC 38:7-sn2 | 804.6 | 184.1 | 11.6 | 45 | 80 |
| PC 36:4 | 782.6 | 184 | 11.7 | 30 | 100 |
| 10-ketostearic acid | 299.3 | 95.1 | 9.3 | 30 | 80 |
| Ricinoleic acid | 299.3 | 81.1 | 9.4 | 15 | 80 |
| Dioctyl Phthalate | 391.3 | 149 | 11.3 | 30 | 80 |
| SM d33:1 | 689.6 | 184.1 | 11.3 | 45 | 80 |
| PC 40:8 | 830.6 | 184.1 | 11.6 | 45 | 80 |
| fatty amide C22:0 | 340.4 | 88.1 | 11.5 | 30 | 80 |
| PE 34:3 | 714.5 | 573.5 | 11.7 | 30 | 80 |
| PE 38:5e | 752.6 | 361.3 | 6.2 | 30 | 80 |
| 5β-Cholestan-3-one | 369.4 | 287.3 | 9.7 | 15 | 80 |
| PE 36:4 | 740.5 | 599.5 | 10.1 | 15 | 80 |
| DAG 36:4 | 634.5 | 337.3 | 12.6 | 30 | 80 |
| DAG 36:3 | 636.6 | 619.5 | 12.8 | 15 | 80 |

CE: collision energy; DP: declustering potential.

2 Plasma negative ions

| Name | Q1(Da) | Q3(Da) | Expected RT (min) | CE(eV) | DP (eV) |
| --- | --- | --- | --- | --- | --- |
| Succinic acid | 117 | 99.9 | 0.8 | -30 | -80 |
| Phenylalanine | 164.1 | 148 | 0.8 | -30 | -80 |
| Ethyl 2-methyl-3,4-pentadienoate | 139.1 | 137 | 0.8 | -15 | -80 |
| 2-Propylglutaric acid | 173.1 | 155.1 | 0.8 | -15 | -80 |
| Curcumenone | 233.2 | 187.9 | 0.8 | -15 | -80 |
| 2,3-DIHYDROXYBENZOATE | 153 | 109 | 0.8 | -15 | -80 |
| 3-Hydroxyoctanoate | 159.1 | 131 | 0.8 | -15 | -80 |
| PS 32:2 | 730.5 | 255.2 | 0.8 | -45 | -80 |
| 2-Methylglutaric acid | 145 | 123.9 | 0.8 | -15 | -80 |
| D-Mannose | 215 | 179.1 | 0.8 | -15 | -80 |
| Glutaric acid | 131 | 113 | 0.8 | -15 | -80 |
| L-Glutamic acid | 146 | 128 | 0.8 | -15 | -80 |
| N-Acetylneuraminic acid | 308.1 | 188.1 | 0.8 | -15 | -80 |
| Paracetamol sulfate | 230 | 187.9 | 0.8 | -15 | -80 |
| 2-DEOXY-D-GLUCOSE | 163.1 | 101 | 0.8 | -15 | -80 |
| Haematommic Acid | 195.1 | 160.8 | 0.8 | -30 | -80 |
| Glycerone phosphate | 170 | 127.1 | 0.8 | -30 | -80 |
| Phosphoric acid | 97 | 80 | 0.8 | -30 | -80 |
| 2-OXO 1 | 145 | 101 | 1 | -11 | -40 |
| 2-OXO 2 | 145 | 57 | 1 | -17 | -40 |
| 3-PG 1 | 185 | 97 | 1 | -20 | -40 |
| CIT 1 | 191 | 111 | 1 | -17 | -40 |
| CIT 2 | 191 | 87 | 1 | -22 | -40 |
| F6P 5 | 259 | 199 | 1 | -16 | -70 |
| FBP 1 | 399 | 97 | 1 | -25 | -30 |
| FBP 2 | 339 | 255 | 1 | -33 | -80 |
| FUM 1 | 115 | 98 | 1 | -27 | -80 |
| G6P-1/F6P1 | 259 | 97 | 1 | -20 | -60 |
| G6P-2/F6P2 | 259 | 79 | 1 | -70 | -60 |
| Inosine | 267.1 | 248.9 | 1 | -15 | -80 |
| itaconic acid 1 | 129 | 85 | 1 | -13 | -45 |
| Lactic acid 1 | 89 | 42.9 | 1 | -22 | -110 |
| Lactic acid 2 | 89 | 44.9 | 1 | -14 | -110 |
| MAL 1 | 133 | 115 | 1 | -15 | -40 |
| MAL 2 | 133 | 89 | 1 | -17 | -40 |
| OXA | 131 | 87 | 1 | -38 | -60 |
| PEP 1 | 167 | 79 | 1 | -13 | -40 |
| PYR 1 | 87 | 87 | 1 | -7 | -40 |
| PYR 2 | 87 | 43 | 1 | -7 | -40 |
| 3-Hydroxyhippuric acid | 194 | 160.8 | 1 | -15 | -80 |
| Citric acid | 191 | 111 | 1 | -15 | -80 |
| Creatinine | 112.1 | 68 | 1 | -15 | -80 |
| Lactic acid | 89 | 71 | 1 | -15 | -80 |
| URATE | 167 | 124 | 1 | -15 | -80 |
| Acetylenedicarboxylate | 114 | 70 | 1.1 | -15 | -80 |
| 2-Hydroxy-3-methylbutyric acid | 117.1 | 73 | 1.2 | -15 | -80 |
| (S)-3-Sulfolactate | 169 | 127.1 | 1.2 | -15 | -80 |
| Dl-Glyceraldehyde3-phosphate | 169 | 126.9 | 1.2 | -15 | -80 |
| N-Acetyl-9-O-acetylneuraminate | 350.1 | 170 | 1.2 | -15 | -80 |
| L-Leucine | 130.1 | 84.1 | 1.3 | -15 | -80 |
| 2-Naphthylamine | 142.1 | 99 | 1.3 | -15 | -80 |
| 3-Hydroxybutyrate | 103 | 73 | 1.3 | -15 | -80 |
| Methylthiobenzoic acid | 167 | 142 | 1.3 | -15 | -80 |
| 2-Aminoethylphosphonic acid | 124 | 97 | 1.3 | -15 | -80 |
| Kojic acid | 141 | 139 | 1.4 | -30 | -80 |
| p-Acetamidophenyl-β-D-glucuronide | 326.1 | 150.1 | 1.6 | -30 | -80 |
| D-Pantothenic acid hemicalcium salt | 218.1 | 188.9 | 1.6 | -30 | -80 |
| Tetrahydropentoxyline | 365.1 | 245.1 | 1.7 | -30 | -80 |
| 2-Hydroxy-3-oxopropanoate | 103 | 85 | 1.8 | -15 | -80 |
| 4-Hydroxybutanoic acid | 103 | 101 | 1.8 | -15 | -80 |
| 1-Methylxanthine | 165 | 108 | 1.8 | -30 | -80 |
| L-Tyrosine | 180.1 | 164 | 1.8 | -15 | -80 |
| Acetaminophen glucuronide | 326.1 | 175 | 1.9 | -15 | -80 |
| L-(-)-3-Phenyllactic acid | 165.1 | 148 | 2 | -15 | -80 |
| 2-Methyladipic acid | 159.1 | 141.9 | 2 | -15 | -80 |
| L-Phenylalanine | 164.1 | 147 | 2 | -15 | -80 |
| trans-Cinnamic acid | 147 | 103.1 | 2 | -15 | -80 |
| Salicylaldehyde | 122 | 95 | 2.2 | -15 | -80 |
| Succinic acid 1 | 117 | 73 | 2.4 | -14 | -40 |
| Succinic acid 2 | 117 | 99 | 2.4 | -14 | -40 |
| Hydroiodic acid | 126.9 | 99 | 2.6 | -30 | -80 |
| N-pentadecanoyl-L-Homoserine lactone | 324.3 | 297.2 | 2.6 | -15 | -80 |
| 4-(Methylnitrosamino)-1-(3-pyridyl)-1-butanol | 208.1 | 164.1 | 3.1 | -15 | -80 |
| Indolelactate | 204.1 | 187.1 | 3.1 | -15 | -80 |
| L-Tryptophan | 203.1 | 186.1 | 3.1 | -15 | -80 |
| xi-2-Ethylheptanoic acid | 157.1 | 155.1 | 3.2 | -15 | -80 |
| 2-Hydroxy-2-methylbutyric acid | 117.1 | 71.1 | 3.2 | -15 | -80 |
| Dl-Tyrosine | 180.1 | 165.1 | 3.2 | -30 | -80 |
| 1,7-Dimethylxanthine | 179.1 | 164 | 3.2 | -30 | -80 |
| Dl-P-Hydroxyphenyl lactic acid | 181.1 | 163 | 3.2 | -15 | -80 |
| γ-Glu-Leu | 259.1 | 241.1 | 3.3 | -15 | -80 |
| 4-Hydroxybenzenesulfonic acid | 173 | 93 | 3.4 | -30 | -80 |
| γ-Glutamylphenylalanine | 293.1 | 275.1 | 3.5 | -15 | -80 |
| 4-Pyridoxic acid | 182 | 138.1 | 3.6 | -15 | -80 |
| Methyl jasmonate | 223.1 | 208.1 | 3.6 | -15 | -80 |
| 3-Methylcatechol | 123 | 108 | 3.7 | -15 | -80 |
| 3-Hydroxymugineic acid | 335.1 | 290.1 | 3.7 | -30 | -80 |
| 4-Acetamidophenol | 150.1 | 93 | 3.7 | -30 | -80 |
| alpha-Hydroxyhippuric acid | 194 | 150.1 | 3.7 | -15 | -80 |
| Indoxyl sulfate | 212 | 80 | 3.7 | -30 | -80 |
| DIHYDROROBINETIN | 303 | 269.1 | 3.8 | -45 | -80 |
| Hippuric acid | 178.1 | 134.1 | 3.9 | -15 | -80 |
| Uridine | 243.1 | 153 | 4 | -30 | -100 |
| 3-Methyl-2-Oxovalerate | 129.1 | 101.1 | 4 | -15 | -80 |
| FA 18:4 | 275.2 | 231.2 | 4 | -15 | -80 |
| Traumatic acid | 227.1 | 184.1 | 4.1 | -30 | -80 |
| Dihydro-4-mercapto-5-methyl-3(2H)-thiophenone | 147 | 123.9 | 4.4 | -15 | -80 |
| 5-O-Caffeoylquinic acid methyl ester | 367.1 | 187 | 4.4 | -30 | -80 |
| N-acetyl-O-acetylneuraminate | 350.1 | 186.9 | 4.4 | -15 | -80 |
| γ-L-Glutamyl-S-(2-carboxy-1-propyl)cysteinylglycine | 392.1 | 186.9 | 4.4 | -30 | -80 |
| Methyl 2,4,6-trihydroxybenzoate | 183 | 139.1 | 4.4 | -15 | -80 |
| Para-cresol | 107.1 | 77 | 4.4 | -30 | -80 |
| P-cresol sulfate | 187 | 107.1 | 4.4 | -15 | -80 |
| 2-Naphthalenesulfonic acid | 207 | 80 | 4.5 | -45 | -80 |
| 1-(2-Furanyl)-1-pentanone | 151.1 | 136 | 4.5 | -15 | -80 |
| 3-Hydroxyphenylacetate | 151 | 136 | 4.5 | -15 | -80 |
| trans-Zeatin riboside | 350.1 | 306.2 | 4.5 | -30 | -80 |
| 2-Hydroxytetracosanoic acid | 383.4 | 338.4 | 4.6 | -15 | -80 |
| 1-Deoxy-D-xylulose 5-phosphate | 213 | 169 | 4.6 | -15 | -80 |
| Hydrocortisone | 397.2 | 383 | 4.7 | -30 | -80 |
| Salicyluric acid | 195 | 161.8 | 4.7 | -30 | -80 |
| 5-Methoxyindole-3-acetic acid | 204.1 | 186.1 | 4.8 | -15 | -80 |
| Azelaic acid | 187.1 | 169.1 | 5.1 | -15 | -80 |
| Hexestrol | 269.1 | 251.1 | 5.1 | -15 | -80 |
| p-Tolyl Sulfate | 187 | 159.9 | 5.3 | -15 | -80 |
| Tetrahydroalstonine | 351.2 | 97 | 5.3 | -45 | -80 |
| Cortolone-3-glucuronide | 541.3 | 113 | 5.3 | -45 | -80 |
| Benzophenone-2 | 245 | 165.1 | 5.3 | -30 | -80 |
| Platyphylloside; 1,7-Bis(4-hydroxyphenyl)-5-oxo-3-heptanyl ?-D-glucopyranoside | 475.2 | 170 | 5.4 | -45 | -80 |
| D-Ribose 5-phophate | 229 | 80.9 | 5.4 | -15 | -80 |
| Tetrahydroaldosterone-3-glucuronide | 539.2 | 521.2 | 5.4 | -45 | -80 |
| Calcitetrol | 431.3 | 153 | 5.5 | -30 | -80 |
| 1,4-Cyclohexanedicarboxylic acid | 171.1 | 169 | 5.5 | -30 | -80 |
| 3β-Hydroxypregn-5-en-20-one sulfate | 395.2 | 332.1 | 5.6 | -15 | -80 |
| 5,15-DiHETE | 335.2 | 147.1 | 5.6 | -15 | -80 |
| Pentadecanoic acid | 241.2 | 223.2 | 5.6 | -30 | -80 |
| N-lactoyl-Isoeucine | 201.1 | 183.1 | 5.6 | -15 | -80 |
| Sebacate | 201.1 | 183.1 | 5.6 | -15 | -80 |
| Gentisate aldehyde | 137 | 135 | 5.7 | -15 | -80 |
| Mukoenine B | 346.2 | 144.2 | 5.8 | -15 | -80 |
| N-Decanoylglycine | 228.2 | 211.1 | 5.9 | -15 | -80 |
| 7β,12β-Dihydroxy-5α-cholan-24-oic acid | 391.3 | 170 | 5.9 | -30 | -80 |
| 6-O-Acetylaustroinulin | 363.3 | 347.3 | 5.9 | -30 | -80 |
| PI 36:4 | 857.5 | 303.2 | 5.9 | -45 | -80 |
| 7z,10z,13z-Hexadecatrienoic Acid | 249.2 | 205.2 | 5.9 | -15 | -80 |
| 6-Thiourate | 183 | 153 | 6 | -30 | -80 |
| PI (18:1_22:5) | 909.5 | 283.3 | 6 | -45 | -80 |
| 5,6-Dihydrouracil | 113 | 111 | 6.1 | -15 | -80 |
| FFA 24:4 | 359.3 | 359 | 6.1 | -12 | -100 |
| Uric acid | 167 | 148 | 6.1 | -30 | -80 |
| 5-Sulfosalicylate | 217 | 188.9 | 6.1 | -30 | -80 |
| FA 16:1 | 253.2 | 235.2 | 6.1 | -30 | -80 |
| PI (17:0_20:4) | 871.5 | 303.2 | 6.1 | -45 | -80 |
| γ-Glutamylaspartic acid | 261.1 | 223.8 | 6.2 | -15 | -80 |
| Undecanedioic acid | 215.1 | 197.1 | 6.2 | -15 | -80 |
| PI (18:0_20:4) | 885.5 | 303.2 | 6.2 | -45 | -80 |
| LPI 18:1 | 597.3 | 281.2 | 6.2 | -30 | -80 |
| Acacetin | 283.1 | 163.1 | 6.3 | -30 | -80 |
| Thyroxine | 775.7 | 126.9 | 6.3 | -45 | -80 |
| 3,4-Methylenesebacic acid | 225.1 | 181.1 | 6.4 | -15 | -80 |
| Myristic acid | 227.2 | 209.2 | 6.4 | -30 | -80 |
| (-)-erythro-Anethole glycol 1-glucoside | 343.1 | 298.1 | 6.4 | -30 | -80 |
| (S)-Oleuropeic acid | 183.1 | 165.1 | 6.4 | -15 | -80 |
| Trimethobenzamide | 387.2 | 341.1 | 6.4 | -15 | -80 |
| LPC 22:6 | 612.3 | 554.3 | 6.4 | -45 | -80 |
| 5-Heptyltetrahydro-2-oxo-3-furancarboxylic acid | 227.1 | 183.1 | 6.4 | -15 | -80 |
| LPA 17:1 | 421.2 | 153 | 6.4 | -30 | -80 |
| Glycochenodeoxycholate-3-sulfate | 528.3 | 449.3 | 6.5 | -30 | -80 |
| 2-Methoxyacetaminophen sulfate | 260 | 223.8 | 6.5 | -15 | -80 |
| FA 17:1 | 267.2 | 249.2 | 6.5 | -30 | -80 |
| Deoxycholic acid 3-glucuronide | 567.3 | 113 | 6.6 | -45 | -80 |
| PI (18:0_22:5) | 911.6 | 283.3 | 6.6 | -45 | -80 |
| 5-Hydroxy-4-[3-(2-hydroxy-2-propanyl)-2-oxiranyl]-1-methyl-7-oxabicyclo[4.1.0]hept-3-en-2-one | 239.1 | 221.1 | 6.6 | -15 | -80 |
| CMPF | 239.1 | 195.1 | 6.6 | -15 | -80 |
| Erucic acid | 337.3 | 283.3 | 6.7 | -30 | -80 |
| LPC 18:3 | 562.3 | 504.3 | 6.7 | -30 | -80 |
| Glycocholic acid | 464.3 | 74 | 6.7 | -45 | -80 |
| Hexadecanoyl-hydroxy-sn-glycerophosphocholine (PUT) | 480.3 | 256.2 | 6.7 | -30 | -80 |
| Dodecanedioic acid | 229.1 | 211.1 | 6.7 | -15 | -80 |
| LysoPE(22:6(4Z,7Z,10Z,13Z,16Z,19Z)/0:0) | 524.3 | 466.3 | 6.7 | -45 | -80 |
| 3-carboxy-4-methyl-5-pentyl-2-furanpropanoic acid | 267.1 | 225.2 | 6.9 | -30 | -80 |
| LPC 16:0 | 540.3 | 482.3 | 6.9 | -15 | -80 |
| LPC 20:3 | 590.3 | 532.3 | 6.9 | -30 | -80 |
| Taurodeoxycholic acid | 498.3 | 418.2 | 6.9 | -45 | -80 |
| PI (18:0_18:1) | 863.6 | 283.3 | 7 | -45 | -80 |
| 2,4,5-Trimethoxy-1-allylbenzene | 207.1 | 179.1 | 7 | -15 | -80 |
| FA 18:3 | 277.2 | 259.2 | 7.1 | -30 | -80 |
| ANDS/DHTS | 369.2 | 97 | 7.1 | -50 | -100 |
| Perfluorooctanesulfonic acid | 498.9 | 80 | 7.1 | -45 | -80 |
| FFA 20:5 | 301.2 | 301 | 7.2 | -12 | -100 |
| 16-Hydroxypalmitate | 271.2 | 226.2 | 7.2 | -15 | -80 |
| 1,11-Undecanedicarboxylic acid | 243.2 | 225.1 | 7.2 | -15 | -80 |
| N-Undecanoylglycine | 242.2 | 226.2 | 7.3 | -15 | -80 |
| Tridecanoylglycine | 270.2 | 253.2 | 7.3 | -30 | -80 |
| D-Xylulose 5-phosphate | 229 | 186.9 | 7.4 | -15 | -80 |
| Cholic acid | 407.3 | 343.3 | 7.5 | -45 | -80 |
| Glycochenodeoxycholic acid | 448.3 | 412.3 | 7.5 | -15 | -80 |
| FAHFA 38:3 | 587.5 | 255.2 | 7.5 | -30 | -80 |
| Isosakuranin | 448.1 | 411.3 | 7.5 | -15 | -80 |
| O-2545 (hydrochloride) | 443.3 | 407.3 | 7.5 | -30 | -80 |
| 3α,7α,12α,26-Tetrahydroxy-5β-cholestane | 435.3 | 433.3 | 7.5 | -45 | -80 |
| Albiflorin | 479.2 | 449.1 | 7.5 | -30 | -80 |
| PS (18:0_20:1) | 816.6 | 293.2 | 7.5 | -45 | -80 |
| LPC 18:2 | 564.3 | 506.3 | 7.6 | -30 | -80 |
| Glycoursodeoxycholic acid | 448.3 | 74 | 7.6 | -45 | -80 |
| Taurolithocholic acid 3-sulfate | 562.2 | 482.3 | 7.6 | -45 | -80 |
| Ajmaline | 325.2 | 183 | 7.6 | -45 | -80 |
| Methoxyeugenol | 193.1 | 163.9 | 7.7 | -30 | -80 |
| 3-α,20-α-dihydroxy-5-β-pregnane 3-glucuronide | 495.3 | 113 | 7.7 | -30 | -80 |
| FA 19:2 | 293.2 | 236.1 | 7.7 | -15 | -80 |
| Hydrocortisone 17-valerate | 481.2 | 113 | 7.8 | -30 | -80 |
| 15,16-DiHODE | 311.2 | 223.2 | 7.8 | -30 | -80 |
| Cis-8,11,14-Eicosatrienoic acid | 305.2 | 287.2 | 7.8 | -30 | -80 |
| All-cis-4,7,10,13,16-docosapentaenoic acid | 329.2 | 255.2 | 7.9 | -45 | -80 |
| FA 20:1 | 309.3 | 183 | 7.9 | -45 | -80 |
| FAHFA 30:4 | 473.4 | 455.4 | 7.9 | -30 | -80 |
| PA 32:0 | 647.5 | 255.2 | 8 | -45 | -80 |
| Biliverdin | 581.2 | 537.2 | 8 | -15 | -80 |
| LPA 20:1 | 463.3 | 283.3 | 8.1 | -30 | -80 |
| 3-(3,4-Dihydroxyphenyl)-2-methyl-L-alanine | 421.2 | 374.2 | 8.1 | -45 | -80 |
| Glutamylvaline | 245.1 | 227.1 | 8.1 | -15 | -80 |
| 11'-Carboxy-α-chromanol | 417.3 | 373.3 | 8.1 | -45 | -80 |
| PA 35:2 | 685.5 | 269.2 | 8.2 | -45 | -80 |
| 1-Arachidonoylglycerophosphoinositol | 619.3 | 465.3 | 8.2 | -45 | -80 |
| N-palmitoyl-phosphoethanolamine | 378.2 | 79 | 8.2 | -45 | -80 |
| SHexCer d38:2 | 832.6 | 697.5 | 8.2 | -45 | -80 |
| Stearic acid | 283.3 | 265.3 | 8.2 | -30 | -80 |
| FA 20:2 | 307.3 | 289.3 | 8.2 | -30 | -80 |
| PG 34:2 | 745.5 | 279.2 | 8.3 | -30 | -80 |
| PS 34:2 | 758.5 | 283.3 | 8.3 | -45 | -80 |
| FFA 24:2 | 363.3 | 331 | 8.4 | -30 | -100 |
| 11-deoxy-PGF1α | 339.3 | 322.2 | 8.4 | -30 | -80 |
| Glycocholate | 465.3 | 402.3 | 8.4 | -30 | -80 |
| SHexCer d39:2 | 846.6 | 725.5 | 8.4 | -45 | -80 |
| LPE 16:1 | 450.3 | 253.2 | 8.5 | -30 | -80 |
| LPC 20:5 | 586.3 | 528.3 | 8.6 | -45 | -80 |
| Camelliagenin C | 489.4 | 487.3 | 8.6 | -30 | -80 |
| Deoxycholic acid | 391.3 | 373.3 | 8.6 | -45 | -80 |
| LPC(14:0/0:0) | 512.3 | 465.3 | 8.6 | -45 | -80 |
| PI 32:1 | 807.5 | 255.2 | 8.6 | -45 | -80 |
| 1-(9Z,12Z-Octadecadienoyl)-2-hydroxy-sn-glycero-3-phosphoethanolamine (NMR) | 476.3 | 431.3 | 8.7 | -30 | -80 |
| Cholesterol glucuronide | 561.4 | 178.9 | 8.7 | -30 | -80 |
| Hexadecanedioic acid | 285.2 | 267.2 | 8.7 | -30 | -80 |
| FA 22:3 | 333.3 | 315.3 | 8.7 | -30 | -80 |
| 2'-Hydroxy-4'-methoxychalcone | 253.1 | 221.1 | 8.8 | -30 | -80 |
| 7β,12α-Dihydroxy-5α-cholan-24-oic acid | 391.3 | 389.3 | 8.8 | -45 | -80 |
| PS36:0 | 790.5 | 303.2 | 8.8 | -45 | -80 |
| 6-Deoxocastasterone | 449.4 | 431.3 | 8.8 | -30 | -80 |
| LPC 20:4 | 588.3 | 530.3 | 8.8 | -15 | -80 |
| 1-(4-Hydroxy-3,5-dimethoxyphenyl)-7-(4-hydroxy-3-methoxyphenyl)-3,5-heptanediol | 405.2 | 363.2 | 8.9 | -30 | -80 |
| LPE 18:2 | 476.3 | 279.2 | 8.9 | -30 | -80 |
| LPE 22:6 | 524.3 | 327.2 | 8.9 | -30 | -80 |
| Glycochenodeoxycholic acid 3-glucuronide | 624.3 | 449.3 | 8.9 | -30 | -80 |
| LPE 20:4 | 500.3 | 303.2 | 8.9 | -30 | -80 |
| LPC 22:6 | 612.3 | 553.3 | 8.9 | -30 | -80 |
| LPE 20:2 | 504.3 | 279.2 | 8.9 | -30 | -80 |
| LPC(O-18:0) | 554.4 | 495.4 | 8.9 | -15 | -80 |
| LPA 22:5 | 483.3 | 303.2 | 8.9 | -30 | -80 |
| PA 34:2 | 671.5 | 255.2 | 9 | -45 | -80 |
| LPE 18:1 | 478.3 | 433.3 | 9 | -15 | -80 |
| PA 36:2 | 699.5 | 283.3 | 9 | -45 | -80 |
| LPA 20:4 | 457.2 | 153 | 9.1 | -30 | -80 |
| PEtOH 34:4 | 695.5 | 255.2 | 9.1 | -45 | -80 |
| LPE 22:5 | 526.3 | 329.2 | 9.1 | -30 | -80 |
| PS (18:0_22:4) | 838.6 | 283.3 | 9.1 | -45 | -80 |
| PI 34:2 | 833.5 | 279.2 | 9.2 | -45 | -80 |
| PE 38:5e | 750.5 | 303.2 | 9.2 | -45 | -80 |
| alpha-D-Glucopyranoside, 4-O-[(2E)-1-oxo-3-phenyl-2-propen-1-yl]-beta-D-fructofuranosyl | 517.2 | 472.2 | 9.2 | -45 | -80 |
| FAHFA 34:2 | 533.5 | 255.2 | 9.2 | -45 | -80 |
| LPE 20:3 | 502.3 | 305.2 | 9.2 | -45 | -80 |
| LPC 16:1 | 538.3 | 480.3 | 9.2 | -15 | -80 |
| Oleoyl-L-α-lysophosphatidic acid | 435.3 | 255.2 | 9.2 | -30 | -80 |
| 13-HODE | 295.2 | 277.2 | 9.3 | -30 | -80 |
| FA 19:1 | 295.3 | 183 | 9.3 | -45 | -80 |
| 4-(beta-D-Glucopyranosyloxy)benzyl 2,3-dihydroxy-3-methylbutanoate | 447.2 | 371.1 | 9.3 | -30 | -80 |
| 4-Phospho-L-aspartate | 212 | 157.9 | 9.4 | -30 | -80 |
| 9,10-DHOME | 313.2 | 295.2 | 9.4 | -30 | -80 |
| PEtOH 36:3 | 725.5 | 279.2 | 9.5 | -45 | -80 |
| FAHFA 36:1 | 563.5 | 281.2 | 9.5 | -45 | -80 |
| FAHFA 38:4 | 585.5 | 281.2 | 9.5 | -45 | -80 |
| Cer(d18:0/18:0) | 612.6 | 567.6 | 9.5 | -15 | -80 |
| FAHFA 40:4 | 613.5 | 281.2 | 9.5 | -30 | -80 |
| LPA 20:2 | 461.3 | 281.2 | 9.5 | -30 | -80 |
| LysoPE(22:5(4Z,7Z,10Z,13Z,16Z)/0:0) | 526.3 | 465.3 | 9.5 | -30 | -80 |
| 8(R)-Hydroxy-(5Z,9E,11Z,14Z)-eicosatetraenoic acid | 319.2 | 301.2 | 9.6 | -15 | -80 |
| LPC 22:4 | 616.4 | 557.3 | 9.6 | -30 | -80 |
| LPC 17:0 | 554.3 | 495.3 | 9.7 | -15 | -80 |
| LPG 9:0 | 385.2 | 341.1 | 9.7 | -30 | -80 |
| LPC 20:2 | 592.4 | 533.3 | 9.8 | -30 | -80 |
| PE 36:2 | 742.5 | 279.2 | 9.8 | -45 | -80 |
| HexCer-AP t35:1 | 744.6 | 281.2 | 9.8 | -45 | -80 |
| LPA 20:0 | 465.3 | 99 | 9.8 | -30 | -80 |
| 9,11-methane-epoxy PGF1α | 351.3 | 333.2 | 9.9 | -30 | -80 |
| Heptadecanoic acid | 269.2 | 251.2 | 9.9 | -30 | -80 |
| LPI 16:1 | 569.3 | 253.2 | 10 | -45 | -80 |
| LPC 18:1 | 566.3 | 508.3 | 10 | -15 | -80 |
| Asparagoside A | 577.4 | 465.3 | 10.1 | -30 | -80 |
| Glycodeoxycholic acid | 448.3 | 385.2 | 10.1 | -15 | -80 |
| QH(2) | 251.1 | 235.1 | 10.1 | -30 | -80 |
| FAHFA 40:3 | 615.5 | 283.3 | 10.2 | -30 | -80 |
| Persenone A | 377.3 | 359.3 | 10.2 | -30 | -80 |
| FAHFA 27:1 | 437.4 | 153 | 10.2 | -30 | -80 |
| Dehydroepiandrosterone sulfate | 367.2 | 304.1 | 10.3 | -45 | -80 |
| FAHFA 28:0 | 453.4 | 83.1 | 10.3 | -30 | -80 |
| LPI 18:2 | 595.3 | 534.3 | 10.4 | -15 | -80 |
| LPC 20:1 | 594.4 | 535.4 | 10.4 | -30 | -80 |
| 9'-Carboxy-α-chromanol | 389.3 | 345.3 | 10.4 | -15 | -80 |
| FA 24:0 | 367.4 | 349.3 | 10.6 | -45 | -80 |
| FA 25:0 | 381.4 | 337.3 | 10.6 | -15 | -80 |
| Ganoderiol C | 517.4 | 499.4 | 10.7 | -45 | -80 |
| 5b-Cholestane-3a,7a,12a,24,25-pentol | 451.3 | 433.3 | 10.8 | -30 | -80 |
| 27-Nor-5b-cholestane-3a,7a,12a,24,25-pentol | 437.3 | 267.2 | 11 | -30 | -80 |
| 12,13-DHOME | 313.2 | 296.2 | 11 | -30 | -80 |
| FA 19:0 | 297.3 | 183 | 11.1 | -45 | -80 |
| FAHFA 32:1 | 507.4 | 253.2 | 11.1 | -45 | -80 |
| (R)-2-hydroxystearic acid | 299.3 | 253.3 | 11.1 | -30 | -80 |
| Arachidonic acid | 303.2 | 259.2 | 11.2 | -15 | -80 |
| Pyroglutamic acid | 128 | 100 | 11.3 | -15 | -80 |
| 8(R)-HPODE | 311.2 | 294.2 | 11.3 | -15 | -80 |
| β-Linoleic acid | 279.2 | 261.2 | 11.3 | -30 | -80 |
| (S)-(+)-Mandelic acid | 151 | 123 | 11.3 | -15 | -80 |
| 2-Propylpentanoic acid | 143.1 | 101.1 | 11.9 | -30 | -80 |
| FA 20:0 | 311.3 | 183 | 12.3 | -45 | -80 |
| 6-Gingerol | 293.2 | 97 | 12.7 | -30 | -80 |
| Pyridoxine | 150.1 | 123 | 12.8 | -15 | -80 |
| Heneicosanoic acid | 325.3 | 183 | 12.8 | -45 | -80 |

CE: collision energy; DP: declustering potential.
