# Supplementary material for: Biomarkers of Metabolomics in Inflammatory Bowel Disease and Damp-Heat Syndrome: A Preliminary Study
Source: Evid Based Complement Alternat Med. 2022 Jul 1;2022:3319646. doi: 10.1155/2022/3319646 (PMC9270137; doi:10.1155/2022/3319646)
Supplement: Supplementary Materials — Supplementary Material 1: damp-heat syndrome and spleen deficiency syndrome diagnosis standards and untargeted metabolomics based UHPLC-HRMS and multilevel metabolite characterization system. Supplementary Material 2: plasma ion-pairs information in positive mode and negative mode. Supplementary Material 3: pseudotargeted metabolomics method of urine samples. Supplementary Material 4: urine ion-pairs information in positive mode and negative mode. Supplementary Material 5: plasma and urine differential metabolites in IBD versus Con; plasma differential metabolites in IBD-DH versus IBD-SD. [file 3319646.f1.zip › 3319646.f1/Supplementary material 3 (2).doc]

Pseudotargeted metabolomics method of urine samples

1 Sample Separation

Samples were allowed to thaw on ice. 200 µL of methanol (precooled at -20℃, containing 13 internal standards with the concentrations are as follows: carnitine C2:0-d3: 0.08µg/mL; carnitine C8:0-d3: 0.05µg/mL; carnitine C10:0-d3: 0.05µg/mL; carnitine C16:0-d3: 0.075µg/mL; LPC 19:0: 0.375µg/mL; FFA C16:0-d3: 1.25µg/mL; FFA C18:0-d3: 1.25µg/mL; CDCA-d4: 0.375µg/mL; CA-d4:0.925µg/mL; Trp-d5: 2.125µg/mL; Phe-d5: 1.8µg/mL; SM 12:0: 0.375µg/mL; choline-d4: 1.0µg/mL) were added to 50 µL of urine for protein precipitation. The mixture was homogenized (Vortex, 1min) at room temperature, stood for 10 min at 4℃, and centrifuged at 14000*g* and 4℃ for 15 min. 200 µL of the supernatant was transferred into a new EP tube, concentrated by 4℃ centrifugation, and stored at -20℃. Prior to analysis, the concentrated metabolite extract sample was reconstituted in 100 µL of 20% methanol/water solution, vortex-mixed, then centrifuged at 14000 *g* and 4℃ for 15 min and 70 µL of supernatant was transferred to a liquid chromatography sample vial for liquid chromatography-mass spectrometry analysis.

2 Quality control (QC) samples and Blank sequences

The Blank sample was a 20% methanol/water solution. For quality control samples, an equal amount of each sample extract was pooled in a glass vial to create a quality control sample. The pretreatment of QC samples was the same as that of analyzed samples, and after every ten runs one QC sample was inserted. This analysis contains four blank sample and six QC samples.

3 Data Acquisition

(1)	Selection ion-pairs information by ultra-high-performance liquid chromatography-high-resolution mass spectrometry (UHPLC-HRMS).

To obtain comprehensive metabolite information two QC samples were subjected to untargeted metabolomics analysis based UHPLC-HRMS (Supplementary Material 1). Based on the multi-level metabolite characterization system (Supplementary Material 1), we used the One-MAP platform to integrate and analyze the results of qualitative annotation analysis at different levels to obtain preliminary qualitative results. Screening of ion-pairs from untargeted qualitative data. Test different collision energies with the AB5500 MASS SPECTROMETER to obtain the most suitable collision energy. The ion pair list from previously targeted assay compound, declustering potential (DP) and collision energy were obtained to supplement the ion-pair list. Based on the ion-pairs list (Supplementary Material 4), all samples were analyzed by ultra-high-performance liquid chromatography–triple-quadrupole mass spectrometry (UHPLC-TQMS) method.

(2)	Multi reaction monitoring (MRM) analysis by UHPLC-TQMS

Chromatographic conditions

Positive ion mode: Chromatographic column: BEH C8 column (1.7 µm, 2.1×100 mm) Waters Corporation, USA; column temperature: 50°C; injection volume: 5 µL; flow rate: 0.35 mL/min; mobile phase A: 0.1% formic acid/water; mobile phase B: 0.1% formic acid/acetonitrile; gradient elution procedure: 5% of phase B as the starting concentration, 0-1 min; phase B changes from 5% to 100%, 1.1-11 min; phase B remains at 100%, 11.1-13 min; phase B remains at 5%,13.1-15 min.

Negative ion mode: Chromatographic column: HSS T3 column (1.8 µm, 2.1×100 mm) Waters Corporation, USA; column temperature: 50°C; injection volume: 5 µL; flow rate: 0.35 mL/min; mobile phase A: 0.1% formic acid/water; mobile phase B: 0.1% formic acid/acetonitrile; gradient elution procedure: 5% of phase B as the starting concentration, 0-1 min; phase B changes from 5% to 100%, 1.1-11 min; phase B remains at 100%, 11.1-13 min; phase B remains at 5%,13.1-15 min.

Mass spectrometry conditions

Positive ion mode: Mass spectrometry primary full scan + data dependent acquisition (DDA) secondary ion scan

Turbo V™ ion source Positive mode with primary full scan + secondary ion scan mode. Ion Spray Voltage: 4500 V; Temperature: 550 °C; Curtain Gas: 35.0 psi; Collision Gas: Medium; Ion Source Gas1: 50.0 psi; Ion Source Gas2: 55.0 psi; Entrance Potential: 10.0 V; Collision Cell Exit Potential: 13.0 V.

Negative ion mode: Mass spectrometry primary full scan + DDA secondary ion scan

Turbo V™ ion source Negative mode with primary full scan + secondary ion scan mode. on Spray Voltage: 4500 V; Temperature: 550 °C; Curtain Gas: 35.0 psi; Collision Gas: Medium; Ion Source Gas1: 50.0 psi; Ion Source Gas2: 55.0 psi; Entrance Potential: 10.0 V; Collision Cell Exit Potential: 16.0 V.

After the instrument is stabilized, one blank sample information is collected first, then two QC sample information is collected, then the actual sample information is started to be collected, and thereafter one QC sample is collected every ten actual samples, interspersed. After all samples are collected, one QC sample is collected at the end. All measurement data were collected by Analyst® TF data collection software (AB SCIEX INSTRUMENTS, USA).

4 Data Processing and Statistical Analysis

(1) Data pre-processing process

The urine specific gravity analysis revealed that almost all subjects had a urine specific gravity between 1.001-1.030. The differences between IBD and Con, IBD-DH and IBD-SD were not statistically significant. Moreover, all subjects were free from associated nephropathy. This indicates that the urine concentration in this study was moderate, so urine normalization was not performed in the actual analysis.

In the first step, data were obtained by extracting UHPLC-HRMS using XCMS software, then peak matching and retention time drift correction were performed, and finally peak alignment was performed after filling in the missing values to obtain the peak table in One-Map for characterization. In the second step, the OS software was used to analyze the data obtained by UHPLC- TQMS, firstly by matching, filling in the missing values, and then by selecting the target characteristic peaks and quantitatively analyzing the peak areas. In the process of extracting the data the software simultaneously completes the noise filtering, retention time, peak type identification, calculation of peak area and other processes according to the set parameters. After manual compounding and excluding abnormal compounds, statistical analysis was performed.

(2) Statistical analysis was handled in the same way as plasma samples
