# Supplementary material for: Biomarkers of Metabolomics in Inflammatory Bowel Disease and Damp-Heat Syndrome: A Preliminary Study
Source: Evid Based Complement Alternat Med. 2022 Jul 1;2022:3319646. doi: 10.1155/2022/3319646 (PMC9270137; doi:10.1155/2022/3319646)
Supplement: Supplementary Materials — Supplementary Material 1: damp-heat syndrome and spleen deficiency syndrome diagnosis standards and untargeted metabolomics based UHPLC-HRMS and multilevel metabolite characterization system. Supplementary Material 2: plasma ion-pairs information in positive mode and negative mode. Supplementary Material 3: pseudotargeted metabolomics method of urine samples. Supplementary Material 4: urine ion-pairs information in positive mode and negative mode. Supplementary Material 5: plasma and urine differential metabolites in IBD versus Con; plasma differential metabolites in IBD-DH versus IBD-SD. [file 3319646.f1.zip › 3319646.f1/Supplementary material 4 (2).doc]

1 Urine positive ions

| Name | Q1(Da) | Q3(Da) | Expected RT (min) | DP (eV) | CE (eV) |
| --- | --- | --- | --- | --- | --- |
| 5-Oxoproline | 130.0 | 114.1 | 1.04 | 80 | 30 |
| ADENINE | 136.1 | 134.1 | 1.04 | 80 | 45 |
| 1-Methylguanine | 166.1 | 149.0 | 1.05 | 80 | 30 |
| Dl-Tyrosine | 182.1 | 167.0 | 1.05 | 80 | 45 |
| L-Norvaline | 118.1 | 116.1 | 1.05 | 80 | 30 |
| L-Tyrosine | 182.1 | 166.1 | 1.05 | 80 | 30 |
| Mesalamine | 154.0 | 137.0 | 1.05 | 80 | 15 |
| N-Benzylformamide | 136.1 | 134.1 | 1.05 | 80 | 30 |
| O-Acetylcarnitine | 204.1 | 145.0 | 1.05 | 80 | 45 |
| α-Amylcinnamaldehyde | 203.1 | 186.1 | 1.05 | 80 | 15 |
| 4-Amino-2-nitrotoluene | 153.1 | 136.0 | 1.06 | 80 | 15 |
| 6-Acetamido-2-oxohexanoate | 188.1 | 170.1 | 1.06 | 80 | 45 |
| 7-Methylguanine | 166.1 | 153.0 | 1.06 | 80 | 45 |
| L-Acetylcarnitine | 204.1 | 145.0 | 1.06 | 80 | 45 |
| 2iP(N6-Isopentenyladenine ) | 204.1 | 146.1 | 1.07 | 80 | 15 |
| 2'-O-Methylcytidine | 258.1 | 256.1 | 1.07 | 80 | 15 |
| 3,4-Dihydroxyphenylethyleneglycol | 153.1 | 137.1 | 1.07 | 80 | 30 |
| Acetylcarnitine | 205.1 | 146.1 | 1.07 | 80 | 15 |
| 1-Methylguanosine | 298.1 | 190.0 | 1.08 | 80 | 45 |
| 2,4-Dimethylquinoline | 158.1 | 156.1 | 1.08 | 80 | 30 |
| 4,6-dihydroxy-3-methyl-2,3-dihydroisoindol-1-one | 180.1 | 105.0 | 1.08 | 80 | 45 |
| 4-Guanidinobutanoate | 146.1 | 130.0 | 1.08 | 80 | 30 |
| Ala-trp | 276.1 | 274.1 | 1.09 | 80 | 15 |
| Ethionamide | 167.1 | 153.0 | 1.09 | 80 | 15 |
| 2-Methyl-5-propyloxazole | 126.1 | 124.1 | 1.09 | 80 | 45 |
| 4-Pyridoxic acid | 184.1 | 167.1 | 1.09 | 80 | 30 |
| Methyldopa anhydrous | 212.1 | 197.1 | 1.09 | 80 | 45 |
| N7-Methylguanosine | 298.1 | 280.1 | 1.09 | 80 | 30 |
| 3-Methylxanthine | 167.1 | 152.1 | 1.10 | 80 | 30 |
| 5-Butyloxazole | 126.1 | 124.1 | 1.11 | 80 | 30 |
| Gly-Tyr | 239.1 | 221.1 | 1.11 | 80 | 15 |
| 3,4-Dihydro-5-propanoyl-2H-pyrrole | 126.1 | 124.1 | 1.12 | 80 | 15 |
| Dihydroactindiolide | 163.1 | 161.1 | 1.12 | 80 | 30 |
| 4-methoxy-2-(5-methoxy-3-methylpyrazol-1-yl)-6-methylpyrimidine | 235.1 | 211.9 | 1.13 | 80 | 15 |
| Isoleucylproline | 229.2 | 169.1 | 1.13 | 80 | 15 |
| N-Formylnornicotine | 191.1 | 174.1 | 1.13 | 80 | 45 |
| N-Hydroxyl-tryptamine | 177.1 | 162.1 | 1.13 | 80 | 45 |
| Prolyl-Threonine | 217.1 | 175.1 | 1.13 | 80 | 45 |
| 5-Methoxytryptamine | 191.1 | 174.1 | 1.14 | 80 | 30 |
| Dethiobiotin | 215.1 | 175.1 | 1.14 | 80 | 30 |
| L-Canavanine | 177.1 | 160.0 | 1.14 | 80 | 15 |
| Pyridoxal | 168.1 | 166.1 | 1.14 | 80 | 15 |
| 3-Hydroxyaminophenol | 126.1 | 124.0 | 1.15 | 80 | 30 |
| 5,7-Dimethoxyflavone | 283.1 | 152.1 | 1.15 | 80 | 30 |
| D-Desthiobiotin | 215.1 | 199.1 | 1.15 | 80 | 15 |
| p-Acetamidophenylglucuronide | 345.1 | 329.1 | 1.15 | 80 | 15 |
| N-(5-Amino-2-hydroxybenzoyl)glycine | 211.1 | 195.1 | 1.17 | 80 | 45 |
| 4-Acetamidobutyric acid | 146.1 | 144.1 | 1.18 | 80 | 30 |
| L-prolyl-L-proline | 213.1 | 166.1 | 1.18 | 80 | 45 |
| 3-Methylguanine | 166.1 | 149.0 | 1.20 | 80 | 15 |
| 5-aminosalicyluric acid | 211.1 | 195.1 | 1.20 | 80 | 15 |
| Pro Pro | 213.1 | 195.1 | 1.20 | 80 | 30 |
| MeIQ | 213.1 | 211.1 | 1.21 | 80 | 15 |
| AMCC | 221.1 | 184.0 | 1.21 | 80 | 15 |
| 2-Hydroxy-6-methylpyridin-3-carboxylic acid | 154.0 | 137.1 | 1.25 | 80 | 15 |
| N-methyl-2,4-Dihydroxy-3-phenylquinoline | 252.1 | 236.1 | 1.25 | 80 | 15 |
| (1S,2S)-Nicotine1-oxide | 179.1 | 177.1 | 1.26 | 80 | 45 |
| 7-Deoxypancratistatin | 310.1 | 294.1 | 1.26 | 80 | 30 |
| N-[4'-hydroxy-(E)-cinnamoyl]-L-aspartic acid | 280.1 | 264.1 | 1.26 | 80 | 30 |
| Pro-Leu | 229.2 | 170.1 | 1.26 | 80 | 45 |
| Serylphenylalanine | 253.1 | 211.9 | 1.26 | 80 | 45 |
| Asp Val | 233.1 | 211.9 | 1.27 | 80 | 45 |
| Xanthurenate-8-O-β-D-glucoside | 368.1 | 310.0 | 1.27 | 80 | 15 |
| 1,2-Diamino-4-nitrobenzene | 154.1 | 137.0 | 1.29 | 80 | 15 |
| N-(1-Deoxy-1-fructosyl)isoleucine | 294.2 | 277.1 | 1.29 | 80 | 30 |
| N4-Acetylcytidine | 286.1 | 269.1 | 1.29 | 80 | 15 |
| N6-Acetyl-LL-2,6-diaminoheptanedioate | 233.1 | 216.1 | 1.29 | 80 | 30 |
| N-Benzyl-2-nitro-1H-imidazole-1-acetamide22994-85-0 | 261.1 | 180.1 | 1.29 | 80 | 45 |
| Phenylacetaldehyde | 121.1 | 114.0 | 1.29 | 80 | 30 |
| 3-(3,4-Dihydroxyphenyl)-2-methyl-L-alanine | 212.1 | 195.1 | 1.30 | 80 | 45 |
| 6-Methylaminopurine arabinoside | 282.1 | 280.1 | 1.30 | 80 | 45 |
| L-Leucine | 132.1 | 114.0 | 1.30 | 80 | 30 |
| Thymine | 127.0 | 114.5 | 1.30 | 80 | 45 |
| 1-Methyladenosine | 282.1 | 280.1 | 1.32 | 80 | 15 |
| 5-Hydroxy-L-tryptophan | 221.1 | 205.1 | 1.32 | 80 | 30 |
| Zalcitabine | 212.1 | 197.1 | 1.32 | 80 | 15 |
| Asp Tyr | 297.1 | 280.1 | 1.33 | 80 | 30 |
| Traumatic acid | 229.2 | 170.1 | 1.33 | 80 | 30 |
| Alanyl-Tryptophan | 276.1 | 252.9 | 1.34 | 80 | 15 |
| KINETIN RIBOSIDE | 348.1 | 218.1 | 1.35 | 80 | 30 |
| 13-METHYL-4,4-BISNOR-8,11,13-PODOCARPATRIEN-3-ONE | 229.2 | 170.1 | 1.36 | 80 | 15 |
| Tranexamic acid | 158.1 | 156.1 | 1.36 | 80 | 45 |
| N6-Succinyl adenosine | 384.1 | 253.1 | 1.39 | 80 | 15 |
| Pyrogallol-2-O-glucuronide | 303.1 | 287.1 | 1.39 | 80 | 30 |
| 1,2,3-Trihydroxybenzene | 127.0 | 114.5 | 1.40 | 80 | 45 |
| 2-[(5-Methylsulfinyl)-4-penten-2-ynylidene]-1,6-dioxaspiro[4.4]non-3-ene | 251.1 | 192.1 | 1.40 | 80 | 45 |
| Valyl-Aspartate | 233.1 | 211.9 | 1.42 | 80 | 45 |
| Cybutryne | 254.1 | 252.1 | 1.43 | 80 | 30 |
| Geranylbenzoquinone | 245.2 | 243.1 | 1.44 | 80 | 30 |
| Biotin | 245.1 | 182.1 | 1.45 | 80 | 30 |
| 2-Amino-5-hydroxybenzoic acid | 154.0 | 137.0 | 1.50 | 80 | 45 |
| [Dihydrolipoyllysine-residue acetyltransferase] S-acetyldihydrolipoyllysine | 250.1 | 234.1 | 1.51 | 80 | 15 |
| 6-Maleimidocaproic acid | 212.1 | 195.1 | 1.51 | 80 | 45 |
| 1-Isobutanol | 454.2 | 437.2 | 1.52 | 80 | 15 |
| γ-Glutamyltyrosine | 311.1 | 295.1 | 1.52 | 80 | 30 |
| METHIONYL-LEUCYLPHENYLALANINE | 410.2 | 329.0 | 1.54 | 80 | 15 |
| 3-Oxo-3-ureidopropanoate | 147.0 | 137.0 | 1.55 | 80 | 30 |
| L-Ethionine | 164.1 | 162.1 | 1.55 | 80 | 30 |
| N-Carbobenzoxyglycylproline | 307.1 | 291.0 | 1.55 | 80 | 15 |
| Asp-phe | 281.1 | 154.1 | 1.57 | 80 | 45 |
| Dehydroepiandrosterone | 271.2 | 253.1 | 1.57 | 80 | 30 |
| 1α,5α-Epidithio-17a-oxa-D-homoandrostan-3,17-dione | 367.1 | 351.1 | 1.58 | 80 | 15 |
| Benzaldehyde | 107.0 | 105.0 | 1.58 | 80 | 30 |
| (4R,6aR,9S,9aR,9bR)-9-Methyl-3,6-bis(methylene)-2,8-dioxododecahydroazuleno[4,5-b]furan-4-yl (2E)-4-hydroxy-2-({[(2E)-4-hydroxy-2-methyl-2-butenoyl]oxy}methyl)-2-butenoate | 475.2 | 269.1 | 1.59 | 80 | 30 |
| N2,N2-Dimethylguanosine | 312.1 | 181.1 | 1.60 | 80 | 45 |
| Tricine | 180.1 | 176.1 | 1.60 | 80 | 45 |
| Tsukushinamine A | 245.2 | 243.1 | 1.60 | 80 | 15 |
| Glu Val | 247.1 | 230.1 | 1.61 | 80 | 45 |
| Norisodomesticine | 312.1 | 181.1 | 1.61 | 80 | 45 |
| Propionyl-L-carnitine | 218.1 | 183.1 | 1.61 | 80 | 30 |
| Tyramine | 138.1 | 136.1 | 1.62 | 80 | 30 |
| β-D-Glucosamine | 180.1 | 164.1 | 1.62 | 80 | 15 |
| γ-Glutamylvaline | 247.1 | 231.1 | 1.62 | 80 | 30 |
| γ-L-Glutamyl-L-valine | 247.1 | 231.1 | 1.63 | 80 | 15 |
| 3-Dehydrocarnitine | 160.1 | 142.1 | 1.67 | 80 | 45 |
| Ubiquinone-2 | 319.2 | 295.9 | 1.67 | 80 | 15 |
| D-Alanyl-D-alanine | 161.1 | 142.1 | 1.68 | 80 | 30 |
| (2R)-2-Hydroxy-2-methylbutanenitrile | 100.1 | 82.1 | 1.69 | 80 | 15 |
| N-Isobutyrylglycine | 146.1 | 144.1 | 1.69 | 80 | 15 |
| Pyridoxamine | 169.1 | 167.1 | 1.70 | 80 | 30 |
| Zilpaterol | 262.2 | 260.1 | 1.70 | 80 | 15 |
| 1H-Indole-4-acetic acid, 2,3-dihydro-2-oxo- | 192.1 | 175.1 | 1.72 | 80 | 45 |
| 2-Aminoacetophenone | 136.1 | 134.1 | 1.72 | 80 | 45 |
| Kynurenine | 209.1 | 195.1 | 1.72 | 80 | 45 |
| 3-Hydroxyhippuric acid | 196.1 | 179.0 | 1.73 | 80 | 15 |
| 4-Hydroxyphenylacetylglutamic acid | 282.1 | 266.1 | 1.74 | 80 | 45 |
| 5-Hydroxy-3-indoleacetic acid | 192.1 | 175.1 | 1.74 | 80 | 30 |
| Kinurenine | 209.1 | 193.1 | 1.76 | 80 | 15 |
| N-(2-Furoyl)glycine | 170.0 | 154.1 | 1.76 | 80 | 30 |
| Levamisole | 205.1 | 188.1 | 1.77 | 80 | 30 |
| L-Phenylalanine-d5 | 171.1 | 169.1 | 1.81 | 80 | 30 |
| 5'-Hydroxysulfapyridine glucuronide | 442.1 | 268.1 | 1.82 | 80 | 30 |
| D-Pantothenic acid | 220.1 | 202.1 | 1.82 | 80 | 45 |
| D-Phenylalanine | 166.1 | 131.0 | 1.83 | 80 | 45 |
| Indoline | 120.1 | 111.0 | 1.83 | 80 | 45 |
| D-Glucosamine-6-phosphate | 260.1 | 238.0 | 1.84 | 80 | 15 |
| (E)-3-(4-hydroxy-3-methoxyphenyl)prop-2-enamide | 216.1 | 190.0 | 1.84 | 80 | 45 |
| N-Hydroxy-L-tryptophan | 221.1 | 205.1 | 1.84 | 80 | 30 |
| 3-(4-Hydroxyphenyl)-propionic acid | 149.1 | 147.0 | 1.85 | 80 | 30 |
| Jasminine | 221.1 | 219.1 | 1.85 | 80 | 15 |
| 4-Nitroanisole | 154.0 | 138.1 | 1.91 | 80 | 45 |
| 4-Acetamidophenol | 152.1 | 138.1 | 1.92 | 80 | 30 |
| Tryptophyl-Asparagine | 319.1 | 296.0 | 1.92 | 80 | 15 |
| 3-Hydroxyestra-1,3,5(10),6-tetraen-17-one | 269.1 | 188.9 | 1.93 | 80 | 45 |
| Nicotinamide | 123.1 | 121.1 | 1.95 | 80 | 45 |
| Methionyl-Hydroxyproline | 263.1 | 247.2 | 1.98 | 80 | 30 |
| Indoxyl glucuronide | 310.1 | 292.2 | 2.02 | 80 | 30 |
| Xanthurenic acid | 206.0 | 182.0 | 2.03 | 80 | 15 |
| 1,3-Cyclobutanedicarboxylic acid, 2,4-bis(4-hydroxyphenyl)-, monomethyl ester | 343.1 | 327.2 | 2.12 | 80 | 15 |
| 3'-Aenylic acid | 348.1 | 249.2 | 2.12 | 80 | 15 |
| 5,6-Dihydroxyindole | 150.1 | 148.0 | 2.13 | 80 | 30 |
| 3-Hydroxyisovalerylcarnitine | 262.2 | 260.1 | 2.14 | 80 | 30 |
| Isoquinoline, 6,7-dimethoxy-1-methyl-1,2,3,4-tetrahydro, hydrochloride2984-97-0 | 208.1 | 206.0 | 2.14 | 80 | 45 |
| Mecambrine | 296.1 | 151.1 | 2.14 | 80 | 15 |
| Pyridoxine | 170.1 | 154.1 | 2.22 | 80 | 15 |
| STICTIC ACID | 387.1 | 370.1 | 2.23 | 80 | 30 |
| Dihyroxy-1H-indole glucuronide I | 326.1 | 224.9 | 2.24 | 80 | 45 |
| Echothiophate | 256.1 | 239.1 | 2.25 | 80 | 15 |
| Adenosine 5'-monophosphate | 348.1 | 249.2 | 2.26 | 80 | 15 |
| Pyridylacetylglycine | 195.1 | 178.0 | 2.27 | 80 | 45 |
| p-Aminohippuric acid | 195.1 | 178.0 | 2.29 | 80 | 30 |
| S-Acetylthioethanolamine | 120.0 | 111.0 | 2.29 | 80 | 30 |
| N-(3-acetamidopropyl)pyrrolidin-2-one | 185.1 | 167.1 | 2.30 | 80 | 30 |
| Pheneturide | 207.1 | 191.1 | 2.30 | 80 | 30 |
| Cyclohexylamine | 100.1 | 96.0 | 2.32 | 80 | 45 |
| S-(Indolylmethylthiohydroximoyl)-L-cysteine | 294.1 | 277.1 | 2.37 | 80 | 15 |
| N1-Methyl-2-pyridone-5-carboxamide | 153.1 | 151.1 | 2.40 | 80 | 30 |
| Biopterin | 238.1 | 212.0 | 2.42 | 80 | 45 |
| 4-Aminosalicylate | 154.0 | 137.0 | 2.44 | 80 | 45 |
| 2-Carboxybenzaldehyde | 151.0 | 149.0 | 2.45 | 80 | 30 |
| Prenyl-L-cysteine | 190.1 | 169.0 | 2.47 | 80 | 45 |
| L-Noradrenaline | 170.1 | 154.0 | 2.53 | 80 | 30 |
| 11-Methyl-2,7-bis(methylene)-6,12-dioxo-5,14-dioxatricyclo[9.2.1.0~4,8~]tetradec-1(13)-en-9-yl methacrylate | 362.2 | 344.1 | 2.56 | 80 | 15 |
| (αS,βS)-α-Ethyl-α-(4-methoxyphenyl)-β-phenyl-2-pyridineethanol | 334.2 | 332.2 | 2.56 | 80 | 15 |
| 3-Methylglutarylcarnitine | 290.2 | 250.2 | 2.56 | 80 | 30 |
| Triethylamine | 102.1 | 74.1 | 2.64 | 80 | 45 |
| Asn Ile | 246.1 | 224.9 | 2.65 | 80 | 45 |
| 17-phenyl trinor PGF2αamide | 370.2 | 355.2 | 2.66 | 80 | 15 |
| 2-Phenylethyl beta-D-glucopyranoside | 302.2 | 280.9 | 2.66 | 80 | 30 |
| 3-Indolebutyric acid | 204.1 | 187.1 | 2.68 | 80 | 45 |
| Isoleucyl-Aspartate | 247.1 | 224.9 | 2.75 | 80 | 45 |
| 5-Phosphooxy-L-lysine | 243.1 | 227.0 | 2.78 | 80 | 15 |
| Kynurenic acid | 190.0 | 173.1 | 2.78 | 80 | 30 |
| Salidroside | 323.1 | 307.1 | 2.78 | 80 | 30 |
| 6-α-Methylprednisolone | 375.2 | 357.2 | 2.79 | 80 | 15 |
| N-((R)-Pantothenoyl)-L-cysteine | 323.1 | 306.1 | 2.79 | 80 | 15 |
| 4-Nitroimidazole | 114.0 | 96.0 | 2.80 | 80 | 45 |
| Phloroglucinol | 127.0 | 117.0 | 2.80 | 80 | 30 |
| Picolinic acid | 124.0 | 107.1 | 2.80 | 80 | 45 |
| 4-o-Galloylbergenin | 481.1 | 322.1 | 2.82 | 80 | 30 |
| N-Acetyl-β-D-glucosamine | 222.1 | 206.2 | 2.82 | 80 | 15 |
| Nicotinuric acid | 181.1 | 163.0 | 2.83 | 80 | 15 |
| L-β-aspartyl-L-leucine | 247.1 | 242.9 | 2.83 | 80 | 15 |
| Nitrobenzene | 124.0 | 107.1 | 2.83 | 80 | 15 |
| Hexylamine | 102.1 | 74.1 | 2.85 | 80 | 45 |
| Methyl N-(a-methylbutyryl)glycine | 174.1 | 172.1 | 2.85 | 80 | 30 |
| Serylisoleucine | 219.1 | 217.1 | 2.87 | 80 | 45 |
| 6-Hydroxy-5-methoxyindole glucuronide | 340.1 | 323.1 | 2.88 | 80 | 15 |
| Prolyl-Tryptophan | 302.2 | 286.1 | 2.93 | 80 | 15 |
| (S)-ATPA | 229.1 | 212.1 | 2.99 | 80 | 15 |
| Propylthiouracil | 171.1 | 154.1 | 2.99 | 80 | 45 |
| 1-phenylisoquinoline | 206.1 | 189.1 | 3.01 | 80 | 30 |
| 2, 4-Diacetylphloroglucinol | 211.1 | 209.0 | 3.02 | 80 | 15 |
| Histidinyl-γ-glutamate | 284.1 | 268.1 | 3.02 | 80 | 15 |
| N-Acetyl-D-fucosamine | 206.1 | 191.1 | 3.02 | 80 | 15 |
| 4-Quinolinecarboxylic acid | 174.1 | 158.1 | 3.03 | 80 | 45 |
| Quinoline-4-carboxylic acid | 174.1 | 168.0 | 3.08 | 80 | 15 |
| Dihydroxyacidissiminol | 428.2 | 406.9 | 3.09 | 80 | 15 |
| (-)-Jasmonic acid | 211.1 | 194.1 | 3.09 | 80 | 15 |
| 4-(Methylnitrosamino)-1-(3-pyridyl)-1-butanol | 210.1 | 194.1 | 3.09 | 80 | 15 |
| Methyl quinoxaline-2-carboxylate | 189.1 | 171.1 | 3.09 | 80 | 30 |
| 3-Hydroxystachydrine | 160.1 | 118.1 | 3.10 | 80 | 45 |
| 5-Deoxy-5'-methylthioadenosine | 298.1 | 283.2 | 3.10 | 80 | 15 |
| Dl-Indole-3-lactic acid | 188.1 | 171.1 | 3.10 | 80 | 30 |
| L-Tryptophan | 205.1 | 189.1 | 3.10 | 80 | 30 |
| Trp Ile | 318.2 | 295.9 | 3.11 | 80 | 15 |
| 5,7-Dihydroxy-4'-methoxy-8-C-prenyl-3'-(3-hydroxy-3-methylbutyl)flavanone | 441.2 | 396.0 | 3.16 | 80 | 15 |
| 7,8-Dihydro-7-hydroxy-8-S-glutathionyl-benzo[a]pyrene | 576.2 | 206.1 | 3.17 | 80 | 15 |
| Asparaginyl-Aspartic acid | 248.1 | 233.1 | 3.21 | 80 | 15 |
| Asp Leu | 247.1 | 224.9 | 3.21 | 80 | 45 |
| α-Zearalanol | 287.2 | 271.1 | 3.21 | 80 | 15 |
| N-Acetyl-D-quinovosamine | 206.1 | 191.1 | 3.22 | 80 | 15 |
| 5,6,7-trimethoxychromen-2-one | 237.1 | 197.1 | 3.23 | 80 | 45 |
| Dopaxanthin | 391.1 | 197.1 | 3.24 | 80 | 30 |
| Luteone 7-glucoside | 517.2 | 315.1 | 3.24 | 80 | 15 |
| 3-Pyridinaldehyde | 108.0 | 96.0 | 3.25 | 80 | 15 |
| N-Acetyl-5'-hydroxysulfapyridine glucuronide | 484.1 | 463.0 | 3.25 | 80 | 15 |
| 6-Hydroxy-3-succinoylpyridine | 196.1 | 179.1 | 3.25 | 80 | 15 |
| Diethylpropion(metabolite V-glucuronide) | 398.2 | 224.2 | 3.25 | 80 | 30 |
| Esculetin | 196.1 | 180.1 | 3.25 | 80 | 30 |
| N-Isopropylammelide | 171.1 | 154.1 | 3.26 | 80 | 45 |
| 2,4,5-trimethoxybenzoic acid | 213.1 | 197.1 | 3.27 | 80 | 15 |
| β-Nicotinamide mononucleotide | 335.1 | 314.0 | 3.27 | 80 | 15 |
| Creatinine | 114.1 | 112.1 | 3.28 | 80 | 15 |
| N6-Carbamoyl-L-threonyladenosine | 413.1 | 282.1 | 3.28 | 80 | 15 |
| 17-Methylandrosta-2,4-dieno[2,3-d]isoxazol-17β-ol | 328.2 | 286.0 | 3.29 | 80 | 30 |
| N-Acetyl-L-aaline | 160.1 | 118.1 | 3.29 | 80 | 45 |
| γ-Glu-Leu | 261.1 | 198.1 | 3.30 | 80 | 45 |
| Pro Leu | 229.2 | 184.1 | 3.31 | 80 | 45 |
| Aucubin | 347.1 | 330.2 | 3.34 | 80 | 15 |
| Asp Phe | 281.1 | 279.1 | 3.39 | 80 | 15 |
| Trp Leu | 318.2 | 279.0 | 3.39 | 80 | 15 |
| 5-Hydroxyindole | 134.1 | 132.0 | 3.43 | 80 | 30 |
| Scopoletin | 193.0 | 179.0 | 3.43 | 80 | 45 |
| L-N2-(2-Carboxyethyl)arginine | 247.1 | 224.9 | 3.44 | 80 | 45 |
| Phenethylamine | 122.1 | 120.1 | 3.44 | 80 | 45 |
| Pro Trp | 302.2 | 283.2 | 3.45 | 80 | 15 |
| D-(+)-Octopine | 247.1 | 224.9 | 3.47 | 80 | 30 |
| N-Pyruvoyl-5-methoxy-3-hydroxyanthranilate | 254.1 | 238.1 | 3.50 | 80 | 30 |
| PC(O-5:0/0:0)[R] | 328.2 | 254.9 | 3.50 | 80 | 45 |
| Phe Asp | 281.1 | 219.1 | 3.51 | 80 | 45 |
| 1-Phenyl-5-heptene-1,3-diyne | 167.1 | 151.1 | 3.51 | 80 | 15 |
| (2S)-2-[[2-(6-oxobenzo[c]chromen-3-yl)oxyacetyl]amino]-3-phenylpropanoic acid | 418.1 | 399.1 | 3.52 | 80 | 15 |
| L-β-aspartyl-L-phenylalanine | 281.1 | 279.1 | 3.54 | 80 | 30 |
| γ-Glutamylisoleucine | 261.1 | 245.1 | 3.56 | 80 | 30 |
| 2-O-Feruloylhydroxycitric acid | 385.1 | 211.0 | 3.57 | 80 | 30 |
| Leu Pro | 229.2 | 142.1 | 3.57 | 80 | 45 |
| 5-Hydroxy-N-formylkynurenine | 253.1 | 195.1 | 3.58 | 80 | 30 |
| 3-(4-methoxy-phenyl)-isochromen-1-one | 253.1 | 236.1 | 3.59 | 80 | 15 |
| 3-Hydroxybenzyl alcohol | 107.0 | 95.0 | 3.60 | 80 | 15 |
| Lipoyllysine | 335.2 | 313.0 | 3.62 | 80 | 15 |
| 3-Hydroxymethyltriazolopthalazinone glucuronide | 376.1 | 217.1 | 3.63 | 80 | 30 |
| Pantetheine 4'-phosphate | 359.1 | 184.1 | 3.63 | 80 | 30 |
| Nε,Nε,Nε-trimethyllysine | 189.2 | 173.1 | 3.63 | 80 | 30 |
| trans-P-Coumaric acid | 147.0 | 130.1 | 3.63 | 80 | 30 |
| 3,5-Dinitro-L-tyrosine | 272.0 | 256.1 | 3.63 | 80 | 15 |
| (E)-3-[2-[(2S,3R,4S,5S,6R)-3,4,5-trihydroxy-6-(hydroxymethyl) oxan-2-yl]oxyphenyl]prop-2-enoic acid | 344.1 | 325.1 | 3.64 | 80 | 45 |
| (R)C(R)S-S-Propylcysteine sulfoxide | 180.1 | 106.0 | 3.67 | 80 | 45 |
| Cysteinyldopa | 317.1 | 300.1 | 3.67 | 80 | 15 |
| 5-Hydroxysulfapyridine | 266.1 | 249.1 | 3.69 | 80 | 15 |
| 8-C-Methyl-6-hydroxykaempferol 3,6,7-trimethyl ether | 359.1 | 238.1 | 3.69 | 80 | 30 |
| Guanidineacetic acid | 118.1 | 114.0 | 3.69 | 80 | 30 |
| Indole-3-carboxylate | 162.1 | 134.1 | 3.70 | 80 | 15 |
| N-Acetyl-5-hydroxytryptamine | 219.1 | 178.2 | 3.71 | 80 | 45 |
| Quercetin 3-O-glucuronide | 479.1 | 304.1 | 3.74 | 80 | 45 |
| Acetylcysteine | 164.0 | 147.1 | 3.75 | 80 | 30 |
| Asn Trp | 319.1 | 317.1 | 3.75 | 80 | 15 |
| 4,5-Dimethylthiazole | 114.0 | 97.1 | 3.76 | 80 | 45 |
| N-γ-L-Glutamyl-L-phenylalanine | 295.1 | 279.1 | 3.77 | 80 | 15 |
| 1,2-Dihydro-5-hydroxy-2-(1-hydroxy-1-methylethyl)-4-(isobutyryl)-6-phenylfurano[2,3-h][1] benzopyran-8-one | 409.2 | 393.2 | 3.79 | 80 | 15 |
| 4-Prenylresveratrol | 297.1 | 295.1 | 3.79 | 80 | 15 |
| L-Serine-phosphoethanolamine | 229.1 | 212.1 | 3.81 | 80 | 15 |
| 5,6-Dihydrothymine | 129.1 | 114.0 | 3.84 | 80 | 45 |
| phenylacetylglutamine | 265.1 | 175.1 | 3.84 | 80 | 45 |
| 2-Phenylacetamide | 136.1 | 134.1 | 3.86 | 80 | 30 |
| Dl-Glutamic acid | 130.0 | 114.0 | 3.86 | 80 | 30 |
| Phenylacetyl-L-glutamine | 265.1 | 249.1 | 3.86 | 80 | 15 |
| Tyr Lys | 310.2 | 293.1 | 3.86 | 80 | 30 |
| Sakuranetin | 287.1 | 285.1 | 3.87 | 80 | 15 |
| 2-Carboxy-2,3-dihydro-5,6-dihydroxyindole | 196.1 | 179.1 | 3.90 | 80 | 45 |
| Leucylproline | 229.2 | 165.1 | 3.90 | 80 | 30 |
| 2 -(Butylamido)-4-hydroxybutanoic acid | 190.1 | 162.1 | 3.91 | 80 | 45 |
| 4-Amino-6,7-dimethoxy-2-(1-piperazinyl) quinazoline | 290.2 | 288.1 | 3.92 | 80 | 15 |
| 8-Methoxykynurenate | 220.1 | 202.1 | 3.92 | 80 | 15 |
| 3-Hydroxy-N6, N6, N6-trimethyl-L-lysine | 205.2 | 168.1 | 3.94 | 80 | 45 |
| Leu-Pro | 229.2 | 211.1 | 3.94 | 80 | 15 |
| 4-Phenylbutazone | 309.2 | 307.1 | 3.97 | 80 | 15 |
| (±)-Abscisic acid | 229.1 | 212.0 | 3.98 | 80 | 30 |
| Isovalerylalanine | 174.1 | 157.1 | 3.99 | 80 | 45 |
| Riboflavin B2 | 377.1 | 360.1 | 4.01 | 80 | 30 |
| (E)-2-Butenyl-4-methyl-threonine | 188.1 | 149.0 | 4.02 | 80 | 30 |
| Hydroxytyrosol 1-O-glucoside | 317.1 | 300.1 | 4.02 | 80 | 30 |
| N-Feruloylglycine | 252.1 | 178.1 | 4.02 | 80 | 30 |
| Methyl benzoate | 137.1 | 132.0 | 4.05 | 80 | 30 |
| 1-Pyrroline-4-hydroxy-2-carboxylate | 130.0 | 115.0 | 4.06 | 80 | 45 |
| N6-(2-Phenylethyl) adenosine | 372.2 | 147.1 | 4.07 | 80 | 45 |
| Progesterone | 337.2 | 319.2 | 4.07 | 80 | 15 |
| VitB12 | 243.1 | 226.1 | 4.07 | 80 | 30 |
| 2-(2-Aminoethyl) indole | 161.1 | 145.1 | 4.11 | 80 | 45 |
| 3-Methylisoquinoline | 144.1 | 122.0 | 4.11 | 80 | 45 |
| Butyl picolinate | 381.2 | 379.1 | 4.14 | 80 | 15 |
| 2-(2'-Methylthio) ethylmalic acid | 209.0 | 195.1 | 4.16 | 80 | 45 |
| 5-L-Glutamyl-taurine | 255.1 | 239.1 | 4.16 | 80 | 30 |
| Tryptamine | 161.1 | 145.1 | 4.16 | 80 | 30 |
| Isodomedin | 393.2 | 378.1 | 4.17 | 80 | 15 |
| 3-(2'-Methylthio) ethylmalic acid | 209.0 | 192.1 | 4.17 | 80 | 15 |
| 7,4'-Dihydroxyflavone | 255.1 | 239.1 | 4.17 | 80 | 15 |
| p-Anisidine | 124.1 | 122.1 | 4.19 | 80 | 45 |
| 4-Methyumbelliferone | 177.1 | 164.0 | 4.20 | 80 | 30 |
| Dopamine glucuronide | 330.1 | 282.9 | 4.20 | 80 | 30 |
| Valyl-Leucine | 231.2 | 185.2 | 4.20 | 80 | 30 |
| 4-Methoxy-Benzoic acid | 135.0 | 132.0 | 4.21 | 80 | 45 |
| 4,8-Dihydroxyquinoline | 162.1 | 145.1 | 4.22 | 80 | 15 |
| p-Hydroxyphenylacetylglycine | 210.1 | 195.1 | 4.22 | 80 | 45 |
| dCMP | 308.1 | 289.1 | 4.23 | 80 | 15 |
| Evocarpine | 340.3 | 323.3 | 4.23 | 80 | 15 |
| Indole-2-carboxylic acid | 162.1 | 145.1 | 4.23 | 80 | 30 |
| N-Nitroso-N-ethylurea | 118.1 | 114.0 | 4.23 | 80 | 30 |
| 2-Ethylacrylylcarnitine | 244.2 | 229.1 | 4.24 | 80 | 15 |
| 3-Deoxyestradiol | 257.2 | 240.1 | 4.27 | 80 | 45 |
| Methylmalonylcarnitine | 262.1 | 245.1 | 4.27 | 80 | 15 |
| 4-Coumaroylshikimate | 321.1 | 305.2 | 4.28 | 80 | 15 |
| 5-Hydroxyindole-3-acetic acid | 192.1 | 178.0 | 4.28 | 80 | 45 |
| Asp Asn | 248.1 | 232.1 | 4.28 | 80 | 30 |
| 3-Methyleneoxindole | 146.1 | 130.1 | 4.29 | 80 | 15 |
| Indole-3-acetamide | 175.1 | 158.1 | 4.30 | 80 | 45 |
| N-Methylhistamine | 126.1 | 124.0 | 4.32 | 80 | 45 |
| 2,3-Diphenyl-3-(2-pyridinyl) acrylonitrile | 283.1 | 281.1 | 4.33 | 80 | 15 |
| Phenolic steroid | 257.2 | 239.1 | 4.36 | 80 | 15 |
| Dihydrodaidzein 7-O-glucuronide | 433.1 | 371.1 | 4.38 | 80 | 30 |
| Luteolin 3'-methyl ether 7-xyloside | 433.1 | 258.1 | 4.38 | 80 | 45 |
| Norwogonin 7-glucuronide | 447.1 | 273.1 | 4.38 | 80 | 45 |
| Genistein 4'-O-glucuronide | 447.1 | 313.1 | 4.39 | 80 | 30 |
| 8-Acetamido-2-methyl-7-oxononanoic acid | 244.2 | 229.1 | 4.41 | 80 | 15 |
| Dopaquinone | 196.1 | 179.1 | 4.41 | 80 | 15 |
| N-Phenylacetylglutamic acid | 266.1 | 248.1 | 4.43 | 80 | 15 |
| 1H-Indole-3-Acetic Acid | 176.1 | 132.1 | 4.44 | 80 | 45 |
| L-1-Pyrroline-3-hydroxy-5-carboxylate | 130.0 | 116.0 | 4.46 | 80 | 15 |
| 2-Aminoadenosine | 283.1 | 281.1 | 4.48 | 80 | 15 |
| Lys Asn | 261.2 | 259.1 | 4.48 | 80 | 15 |
| 2-(2-Hydroxy-phenyl)-3H-quinazolin-4-one | 239.1 | 223.1 | 4.49 | 80 | 15 |
| 2-Methylbutyroylcarnitine | 246.2 | 228.2 | 4.51 | 80 | 30 |
| Ethopabate | 238.1 | 220.1 | 4.51 | 80 | 45 |
| N-Formimino-L-glutamate | 175.1 | 146.1 | 4.51 | 80 | 45 |
| beta-D-Glucopyranoside, 2-methoxy-4-[(1S,3aS,4R,6aR)-tetrahydro-3a-hydroxy-4-(4-hydroxy-3-methoxyphenyl)-1H,3H-furo[3,4-c] furan-1-yl] phenyl | 554.2 | 396.1 | 4.52 | 80 | 15 |
| Isovalerylglutamic acid | 232.1 | 230.1 | 4.52 | 80 | 15 |
| 3,4-Methylenesebacic acid | 227.1 | 191.1 | 4.53 | 80 | 45 |
| Leu Trp | 318.2 | 277.2 | 4.53 | 80 | 15 |
| Taurocholic acid sodium salt hydrate | 516.3 | 179.1 | 4.54 | 80 | 45 |
| Cuminaldehyd | 149.1 | 132.1 | 4.55 | 80 | 45 |
| o-Xylene | 107.1 | 105.1 | 4.55 | 80 | 45 |
| Sebacic acid | 203.1 | 188.1 | 4.56 | 80 | 30 |
| 4,4-Bis(4-hydroxyphenyl) heptane | 285.2 | 269.1 | 4.59 | 80 | 15 |
| Acetylleucine (INN) | 174.1 | 158.0 | 4.59 | 80 | 15 |
| N-palmitoyl taurine | 364.2 | 362.2 | 4.61 | 80 | 30 |
| Glycitein 4'-O-glucuronide | 461.1 | 287.1 | 4.63 | 80 | 15 |
| 8-Methylthiooctyl glucosinolate | 478.1 | 301.1 | 4.64 | 80 | 45 |
| Baicalein 6-methyl ether 7-glucuronide | 461.1 | 287.1 | 4.64 | 80 | 30 |
| 2,6R-Dimethyl-2E-octen-1,8-dioic acid | 201.1 | 184.1 | 4.65 | 80 | 15 |
| N-Octanoyl-L-homoserine lactone | 228.2 | 226.2 | 4.66 | 80 | 15 |
| apigenin-7-O-glucuronide | 447.1 | 273.2 | 4.67 | 80 | 30 |
| Genistein 5-O-glucuronide | 447.1 | 431.1 | 4.67 | 80 | 15 |
| Methyl [(1S,3S,7R,8R,9R,12S,13S)-13-(3-furyl)-6,6,8,12-tetramethyl-17-methylene-5,15-dioxo-2,14-dioxatetracyclo[7.7.1.0~1,12~.0~3,8~]heptadec-7-yl](hydroxy)acetate | 504.3 | 489.2 | 4.67 | 80 | 15 |
| 12-hydroxyjasmonic acid | 227.1 | 191.1 | 4.68 | 80 | 45 |
| 3''-Hydroxy-geranylhydroquinone | 263.2 | 245.2 | 4.68 | 80 | 45 |
| 7-(2-aminophenyl) heptanoic acid | 263.2 | 147.1 | 4.68 | 80 | 15 |
| (5-L-Glutamyl)-L-glutamine | 276.1 | 261.1 | 4.72 | 80 | 15 |
| 5-Hepteneoylglycine | 186.1 | 168.9 | 4.72 | 80 | 45 |
| Guanadrel | 214.2 | 199.1 | 4.72 | 80 | 15 |
| Tyr Thr | 283.1 | 192.1 | 4.72 | 80 | 45 |
| 3-(3-Hydroxyphenyl) propanoic acid | 167.1 | 150.1 | 4.74 | 80 | 45 |
| Leucyl-Methionine | 263.1 | 147.1 | 4.74 | 80 | 45 |
| Dihydrojasmonic acid | 235.1 | 220.1 | 4.75 | 80 | 15 |
| N-lactoyl-Leucine | 204.1 | 188.1 | 4.75 | 80 | 30 |
| tetranor-PGAM | 311.1 | 309.1 | 4.75 | 80 | 15 |
| Glutathione amide | 307.1 | 91.1 | 4.76 | 80 | 15 |
| Prolylphenylalanine | 263.1 | 164.0 | 4.77 | 80 | 30 |
| Phe-Pro | 263.1 | 248.1 | 4.81 | 80 | 15 |
| trans-2,3,4-Trimethoxycinnamic Acid | 239.1 | 222.1 | 4.81 | 80 | 15 |
| Luteolin 7-O-glucuronide | 463.1 | 384.1 | 4.82 | 80 | 30 |
| N-Acetyl-L-phenylalanine | 208.1 | 195.1 | 4.83 | 80 | 45 |
| Hexanoyl glycine | 174.1 | 158.1 | 4.84 | 80 | 30 |
| (2S)-2-Isopropyl-3-oxosuccinate | 175.1 | 158.1 | 4.86 | 80 | 15 |
| 3,4-Dihydroxymandelate | 185.0 | 168.9 | 4.86 | 80 | 45 |
| 3-O-Methylgallate | 185.0 | 169.0 | 4.86 | 80 | 15 |
| 2-Ethylthiazole | 114.0 | 96.0 | 4.89 | 80 | 45 |
| N(6)-[(Indol-3-yl)acetyl]-L-lysine | 304.2 | 302.2 | 4.91 | 80 | 15 |
| Apigenin 7,4'-diglucuronide | 623.1 | 449.1 | 4.92 | 80 | 15 |
| 1-(4-Methoxyphenyl)-1-penten-3-one | 191.1 | 175.1 | 4.94 | 80 | 15 |
| 3,4,5-Trimethoxycinnamic acid | 239.1 | 222.1 | 4.94 | 80 | 15 |
| 8-hydroxy-6-methoxy-7-[(2S,3R,4S,5S,6R)-3,4,5-trihydroxy-6-(hydroxymethyl)oxan-2-yl]oxychromen-2-one | 388.1 | 241.2 | 4.96 | 80 | 45 |
| Kaempferol 3-rhamnoside 7-galacturonide | 609.1 | 273.1 | 4.97 | 80 | 30 |
| Quercetin 3-[6''-(3-hydroxy-3-methylglutaryl)galactoside] | 609.1 | 449.1 | 4.97 | 80 | 15 |
| Apigenin 7-glucuronide | 447.1 | 430.3 | 4.98 | 80 | 30 |
| Shikimate | 175.1 | 158.1 | 4.99 | 80 | 15 |
| (R)-Indenestrol A | 267.1 | 265.1 | 5.00 | 80 | 15 |
| Indolylacryloylglycine | 245.1 | 171.1 | 5.00 | 80 | 45 |
| Phosphodimethylethanolamine | 170.1 | 143.1 | 5.00 | 80 | 45 |
| Tribenzylamine n-oxide | 304.2 | 302.2 | 5.01 | 80 | 15 |
| Geranic acid | 169.1 | 151.1 | 5.03 | 80 | 15 |
| 4-formyl Indole | 146.1 | 130.0 | 5.04 | 80 | 45 |
| (S)-cis-N-Methylstylopine | 338.1 | 321.2 | 5.05 | 80 | 15 |
| 4-Hydroxyquinoline | 146.1 | 130.1 | 5.05 | 80 | 30 |
| Naringenin 7-O-glucuronide | 449.1 | 447.1 | 5.05 | 80 | 15 |
| Quercitrin | 449.1 | 275.1 | 5.05 | 80 | 30 |
| Kaempferol7-O-β-D-glucopyranoside | 449.1 | 274.1 | 5.06 | 80 | 45 |
| (2S,3S)-3,5,7-trihydroxy-6-methyl-2-(3,4,5-trihydroxyphenyl)-2,3-dihydrochromen-4-one | 335.1 | 319.2 | 5.06 | 80 | 15 |
| 3,4-Methylene suberic acid | 199.1 | 197.1 | 5.07 | 80 | 30 |
| N-(3-Indolylacetyl)-L-alanine | 247.1 | 185.0 | 5.07 | 80 | 15 |
| Zearalenone | 319.2 | 211.1 | 5.08 | 80 | 45 |
| Estrone glucuronide | 447.2 | 430.2 | 5.10 | 80 | 15 |
| 4-oxododecanedioic acid | 245.1 | 243.1 | 5.11 | 80 | 15 |
| 5-Methoxyindoleacetate | 206.1 | 132.1 | 5.11 | 80 | 45 |
| Cinnamic acid | 131.0 | 114.0 | 5.11 | 80 | 45 |
| (6E)-8-Hydroxygeraniol | 171.1 | 153.1 | 5.20 | 80 | 15 |
| (±)-Jasmonic acid | 211.1 | 209.0 | 5.29 | 80 | 30 |
| Hesperetin | 303.1 | 243.1 | 5.32 | 80 | 15 |
| Hesperetin 7-O-glucuronide | 479.1 | 305.1 | 5.32 | 80 | 30 |
| (±)α-CMBHC | 321.2 | 231.1 | 5.35 | 80 | 45 |
| 1,4-Dihydroxynaphthalene | 161.1 | 144.1 | 5.35 | 80 | 45 |
| 2-Phenylbutyric acid | 165.1 | 149.1 | 5.35 | 80 | 30 |
| Isoeugenol | 165.1 | 147.1 | 5.35 | 80 | 45 |
| Nicotine | 163.1 | 147.1 | 5.37 | 80 | 15 |
| Baicalin | 447.1 | 273.1 | 5.39 | 80 | 30 |
| 2Z-Dodecenedioic acid | 229.1 | 213.1 | 5.39 | 80 | 15 |
| Estrone | 271.2 | 255.1 | 5.41 | 80 | 15 |
| L-Glutamine | 147.1 | 131.1 | 5.41 | 80 | 45 |
| 17α-Hydroxyprogesterone | 331.2 | 329.2 | 5.43 | 80 | 30 |
| L-Hexanoylcarnitine | 260.2 | 204.9 | 5.46 | 80 | 45 |
| Diosmetin 7-O-β-D-glucuronopyranoside | 477.1 | 311.1 | 5.47 | 80 | 30 |
| Glutamine | 147.1 | 131.1 | 5.47 | 80 | 45 |
| (-)-12-hydroxy-9,10-dihydrojasmonic acid | 229.1 | 213.1 | 5.52 | 80 | 15 |
| Chrysin | 255.1 | 199.1 | 5.53 | 80 | 30 |
| Daidzein | 255.1 | 237.1 | 5.54 | 80 | 45 |
| di-Hydroxymelatonin | 265.1 | 157.0 | 5.54 | 80 | 45 |
| 1,2,4-Tris(methylene)cyclohexane | 121.1 | 107.1 | 5.55 | 80 | 15 |
| 3-Dimethylallyl-4-hydroxybenzaldehyde | 191.1 | 175.1 | 5.55 | 80 | 30 |
| 8-hydroxy-3-methoxycarbonyl-2-methylidenenonanoic acid | 262.2 | 227.1 | 5.55 | 80 | 45 |
| Phosphorylcholine | 170.1 | 149.0 | 5.55 | 80 | 15 |
| 17-α-Hydroxyprogesterone | 331.2 | 329.2 | 5.56 | 80 | 30 |
| 11β,21-Dihydroxy-5β-pregnane-3,20-dione | 349.2 | 331.2 | 5.57 | 80 | 45 |
| Glucosyl (2E,6E,10x)-10,11-dihydroxy-2,6-farnesadienoate | 433.2 | 372.2 | 5.60 | 80 | 30 |
| 11β,17α,21-Trihydroxypregnenolone | 365.2 | 329.2 | 5.64 | 80 | 15 |
| (E)-7-(4-methoxy-5-methyl-6-oxopyran-2-yl)-5-methyloct-6-enoic acid | 295.2 | 256.3 | 5.65 | 80 | 45 |
| 5,2'-Dihydroxy-3,6,7,8,4',5'-hexamethoxyflavone | 435.1 | 260.1 | 5.65 | 80 | 30 |
| Davidigenin | 259.1 | 242.1 | 5.65 | 80 | 15 |
| Glycitein | 285.1 | 271.1 | 5.67 | 80 | 45 |
| 3-hydroxy-C10-homoserine lactone | 272.2 | 214.1 | 5.68 | 80 | 15 |
| Norwogonin 8-glucuronide | 447.1 | 428.2 | 5.68 | 80 | 15 |
| Hydrocortisone | 363.2 | 285.2 | 5.68 | 80 | 30 |
| Apigenin 7-galacturonide | 447.1 | 273.1 | 5.69 | 80 | 45 |
| (±)-Hexanoylcarnitine | 260.2 | 144.1 | 5.74 | 80 | 45 |
| 3α,11β,21-Trihydroxy-20-oxo-5β-pregnan-18-al | 365.2 | 329.2 | 5.77 | 80 | 15 |
| 2-trans,6-trans-Farnesal | 221.2 | 219.1 | 5.77 | 80 | 15 |
| Isorhamnetin 7-α-D-Glucosamine | 478.1 | 303.2 | 5.77 | 80 | 30 |
| Acacetin 7-glucuronide | 461.1 | 287.1 | 5.78 | 80 | 15 |
| Daidzein 7-O-glucuronide | 431.1 | 307.1 | 5.78 | 80 | 30 |
| Glycitein 7-O-glucuronide | 461.1 | 287.1 | 5.78 | 80 | 30 |
| 15-methyl-15S-PGD2 | 349.2 | 301.2 | 5.79 | 80 | 45 |
| 8-(3,4-Dimethoxy-phenyl)-2,3,10,11-tetramethoxy-5,6,13a-tridehydro-berbinium | 488.2 | 471.3 | 5.80 | 80 | 15 |
| Genkwanin | 285.1 | 271.1 | 5.80 | 80 | 30 |
| N6-(L-1,3-Dicarboxypropyl)-L-lysine | 277.1 | 261.2 | 5.80 | 80 | 15 |
| 6-octenoylglycine | 200.1 | 182.1 | 5.82 | 80 | 15 |
| [5-(2-Dimethylamino-acetyl)-10,11-dihydro-5H-dibenzo[b,f]azepin-3-yl]-carbamic acid ethyl ester | 368.2 | 347.1 | 5.86 | 80 | 15 |
| Se-Adenosylselenomethionine | 447.1 | 273.1 | 5.86 | 80 | 15 |
| Genistein 7-O-glucuronide | 447.1 | 286.2 | 5.86 | 80 | 30 |
| 5β-Scymnol sulfate | 549.3 | 464.2 | 5.87 | 80 | 15 |
| Androsta-5,16-dieno[17,16-b]quinolin-3β-ol | 374.3 | 372.2 | 5.87 | 80 | 15 |
| 8-hydroxymianserin glucuronide | 457.2 | 282.2 | 5.92 | 80 | 30 |
| γ-Glutamyl-β-(isoxazolin-5-on-2-yl)alanine | 302.1 | 286.1 | 5.95 | 80 | 30 |
| Tetrahydroaldosterone-3-glucuronide | 541.3 | 367.2 | 5.95 | 80 | 15 |
| Tetrahydrocortisone | 365.2 | 348.2 | 5.96 | 80 | 30 |
| 3,5-DIPRENYL-4-HYDROXYACETOPHENONE | 273.2 | 271.2 | 5.96 | 80 | 15 |
| 17α-Hydroxypregnenolone | 333.2 | 331.2 | 5.98 | 80 | 15 |
| 15-OxoETE | 341.2 | 339.2 | 5.99 | 80 | 15 |
| 4-Androstene-3,17-Dione | 287.2 | 285.1 | 5.99 | 80 | 30 |
| Ubiquinone-1 | 251.1 | 235.1 | 6.01 | 80 | 15 |
| 6-Methoxyluteolin 7-rhamnoside | 463.1 | 341.0 | 6.04 | 80 | 15 |
| malvidin-3-O-arabinoside | 463.1 | 289.2 | 6.05 | 80 | 30 |
| (+)-Epicatechin | 291.1 | 273.2 | 6.06 | 80 | 15 |
| 5,2'-Dihydroxy-7,8,6'-trimethoxyflavone 2'-glucuronide | 521.1 | 440.2 | 6.13 | 80 | 30 |
| 2-dec-9-enyl-3-hydroxypentanedioic acid | 304.2 | 302.1 | 6.14 | 80 | 30 |
| Hydrocortisone succinate | 463.2 | 288.2 | 6.19 | 80 | 15 |
| 6-Dehydrotestosterone glucuronide | 463.2 | 289.1 | 6.20 | 80 | 30 |
| N-Docosahexaenoyl GABA | 414.3 | 412.2 | 6.22 | 80 | 15 |
| 3α-Hydroxyoreadone | 253.1 | 251.1 | 6.24 | 80 | 15 |
| N-Acetylaminooctanoic acid | 202.1 | 179.0 | 6.24 | 80 | 30 |
| Geranylhydroquinone | 247.2 | 231.1 | 6.26 | 80 | 15 |
| Propanoic acid, 3-hydroxy-2-[(4aR,8aS)-octahydro-4a-methyl-8-methylene-2(1H)-naphthalenylidene]-, methyl ester, (2E)- | 247.2 | 224.9 | 6.26 | 80 | 45 |
| 2,3-Dinor-8-iso prostaglandin F2α | 309.2 | 307.2 | 6.26 | 80 | 15 |
| Limocitrin 7-(6''-acetylglucoside) | 551.1 | 454.3 | 6.28 | 80 | 15 |
| 2-Oxo-10-methylthiodecanoic acid | 233.1 | 231.1 | 6.30 | 80 | 30 |
| Glycocholic acid | 466.3 | 449.3 | 6.30 | 80 | 15 |
| 8-hydroxy-3-(4-hydroxypentyl)-3,4-dihydroisochromen-1-one | 251.1 | 233.1 | 6.32 | 80 | 45 |
| Ethynyl estradiol | 297.2 | 281.1 | 6.33 | 80 | 45 |
| α-Eucaine | 334.2 | 332.1 | 6.33 | 80 | 15 |
| Testosterone glucuronide | 465.2 | 290.2 | 6.37 | 80 | 45 |
| trans-Zeatin riboside | 352.2 | 289.1 | 6.39 | 80 | 30 |
| Prostaglandin E2 p-benzamidophenyl ester | 530.3 | 321.3 | 6.39 | 80 | 45 |
| p-Methoxycinnamaldehyde | 163.1 | 139.0 | 6.42 | 80 | 45 |
| (2E,6E,12E)-19-(2-amino-2-oxoethyl)-9,11-dihydroxy-8-methoxy-10,12,14-trimethyl-15-oxohenicosa-2,6,12-trienedioic acid | 526.3 | 297.2 | 6.43 | 80 | 45 |
| Genistein | 271.1 | 257.1 | 6.45 | 80 | 45 |
| Geranylgeranyl diphosphate | 451.2 | 221.2 | 6.47 | 80 | 45 |
| Canrenoic acid potassium salt2181-04-6 | 358.2 | 325.2 | 6.49 | 80 | 15 |
| QH(2) | 253.1 | 237.1 | 6.49 | 80 | 15 |
| delta2-THA | 355.3 | 337.3 | 6.57 | 80 | 30 |
| (S)-Zearalanone | 321.2 | 304.2 | 6.58 | 80 | 30 |
| N-(1-Deoxy-1-fructosyl)proline | 278.1 | 234.1 | 6.58 | 80 | 30 |
| (±)14(15)-EET-SI | 398.2 | 388.3 | 6.59 | 80 | 15 |
| 17(S)-HpDoHE | 343.2 | 330.0 | 6.59 | 80 | 45 |
| β-Zearalenol | 321.2 | 213.1 | 6.59 | 80 | 45 |
| N-Undecanoylglycine | 244.2 | 228.2 | 6.65 | 80 | 15 |
| Tuberonic acid | 227.1 | 186.0 | 6.66 | 80 | 45 |
| Zearalanol | 323.2 | 321.2 | 6.66 | 80 | 15 |
| Leukotriene D4 methyl ester | 511.3 | 299.2 | 6.67 | 80 | 45 |
| 16,16-dimethyl-6-keto Prostaglandin E1 | 379.2 | 197.1 | 6.76 | 80 | 45 |
| Deoxycholic acid | 357.3 | 355.3 | 6.78 | 80 | 15 |
| Docosahexaenoyl Glycine | 386.3 | 369.3 | 6.80 | 80 | 45 |
| tetranor-12(S)-HETE | 249.2 | 224.9 | 6.82 | 80 | 45 |
| 1α,25-dihydroxy-21-norcholecalciferol | 403.3 | 387.3 | 6.83 | 80 | 15 |
| 26:6(8Z,11Z,14Z,17Z,20Z,23Z) | 385.3 | 368.3 | 6.83 | 80 | 15 |
| Androsterone Enanthate | 403.3 | 386.3 | 6.84 | 80 | 30 |
| 3-Hydroxy-19-norpregna-1,3,5(10)-trien-20-one | 299.2 | 283.2 | 6.84 | 80 | 15 |
| epi-4'-hydroxyjasmonic acid | 227.1 | 186.0 | 6.84 | 80 | 45 |
| 16-phenoxy tetranor PGF2α; cyclopropyl methyl amide | 444.3 | 351.2 | 6.85 | 80 | 30 |
| Ovatine | 386.3 | 369.3 | 6.85 | 80 | 30 |
| Tretinoin | 299.2 | 171.2 | 6.85 | 80 | 30 |
| α-Zearalenol | 321.2 | 306.2 | 6.85 | 80 | 45 |
| (17Z)-3,11-Dioxopregna-4,17(20)-dien-21-oic acid methyl ester | 357.2 | 355.1 | 6.85 | 80 | 15 |
| 5α-Pregn-2-en-20-one | 301.3 | 283.2 | 6.90 | 80 | 45 |
| Stercobilin | 595.3 | 472.3 | 6.90 | 80 | 30 |
| PS(18:2(9Z,12Z)/0:0) | 522.3 | 483.1 | 6.91 | 80 | 15 |
| Androstenol | 257.2 | 215.2 | 6.91 | 80 | 45 |
| Cholylglycine | 466.3 | 451.2 | 6.92 | 80 | 15 |
| MGDG 21:4 | 591.3 | 468.2 | 6.93 | 80 | 30 |
| 5,6-Epoxy-18R-HEPE | 333.2 | 309.6 | 6.98 | 80 | 30 |
| D-Urobilinogen | 591.3 | 589.3 | 7.00 | 80 | 15 |
| Androsterone | 273.2 | 255.2 | 7.03 | 80 | 45 |
| Dihydrotestosterone | 273.2 | 256.2 | 7.03 | 80 | 30 |
| (±)-Octanoylcarnitine | 288.2 | 144.1 | 7.04 | 80 | 45 |
| 11β-Hydroxyprogesterone | 330.2 | 254.9 | 7.04 | 80 | 45 |
| 9-cis-Retinoic acid | 301.2 | 299.2 | 7.04 | 80 | 15 |
| 5α,17α-Pregn-2-en-20-yn-17-ol acetate | 341.2 | 324.2 | 7.06 | 80 | 30 |
| PG(22:4(7Z,10Z,13Z,16Z)/0:0) | 561.3 | 521.2 | 7.06 | 80 | 15 |
| Guggulsterone | 313.2 | 311.2 | 7.09 | 80 | 15 |
| 7-Ketodeoxycholic acid | 407.3 | 372.3 | 7.14 | 80 | 30 |
| 12-epi Leukotriene B4-d4 | 323.2 | 307.2 | 7.15 | 80 | 15 |
| Zeatin riboside,trans-isomer | 352.2 | 335.2 | 7.16 | 80 | 15 |
| Cycloprodigiosin | 322.2 | 306.2 | 7.16 | 80 | 15 |
| 5α-Pregnane-3,20-dione | 317.2 | 300.2 | 7.17 | 80 | 30 |
| Biochanin A | 285.1 | 271.1 | 7.17 | 80 | 45 |
| Glycinoeclepin B | 559.3 | 385.3 | 7.17 | 80 | 15 |
| 3-Methyl-19-nor-17α-pregna-1,3,5(10)-trien-17-ol | 299.2 | 282.2 | 7.18 | 80 | 45 |
| 3-hydroxyundecanoyl carnitine | 346.3 | 327.2 | 7.19 | 80 | 15 |
| 4-Nitroestra-1,3,5(10)-triene-3,17β-diol | 318.2 | 287.6 | 7.19 | 80 | 45 |
| 12-Tridecynoic acid | 211.2 | 195.1 | 7.20 | 80 | 45 |
| 3E,5E-tridecadienoic acid | 211.2 | 209.0 | 7.20 | 80 | 15 |
| α-Curcumene | 203.2 | 186.1 | 7.24 | 80 | 45 |
| LAUROYLCARNITINE | 344.3 | 342.2 | 7.32 | 80 | 30 |
| Dodecanoylcarnitine | 344.3 | 342.2 | 7.42 | 80 | 30 |
| Ethisterone | 313.2 | 311.2 | 7.43 | 80 | 15 |
| 17β-Hydroxy-4-oxa-5α-estr-1-en-3-one acetate | 319.2 | 302.2 | 7.44 | 80 | 30 |
| MGDG 14:0 | 501.3 | 327.3 | 7.52 | 80 | 15 |
| 3-hydroxytridecanoyl carnitine | 374.3 | 97.1 | 7.53 | 80 | 45 |
| 2-Octenoylcarnitine | 286.2 | 268.2 | 7.57 | 80 | 15 |
| Medroxyprogesterone acetate | 387.3 | 371.2 | 7.59 | 80 | 15 |
| L-Octanoylcarnitine | 288.2 | 144.1 | 7.59 | 80 | 45 |
| N-benzyl-7H-purin-6-amine1214-39-7 | 226.1 | 181.1 | 7.60 | 80 | 45 |
| 3-Ethoxyandrosta-3,5-dien-17β-ol propanoate | 373.3 | 371.3 | 7.61 | 80 | 15 |
| Chol-11-Enic Acid | 373.3 | 357.3 | 7.61 | 80 | 30 |
| CHOLIC ACID | 426.3 | 374.3 | 7.61 | 80 | 30 |
| Isolithocholate | 377.3 | 360.3 | 7.61 | 80 | 30 |
| (3α,5β,7α)-23-Carboxy-7-hydroxy-24-norcholan-3-yl-β-D-Glucopyranosiduronic acid | 569.3 | 376.3 | 7.62 | 80 | 15 |
| Glycodeoxycholic acid | 450.3 | 432.3 | 7.67 | 80 | 15 |
| cannitine C8:0-d3 | 291.2 | 230.1 | 7.69 | 80 | 30 |
| Nα-Benzoyl-DL-arginine β-naphthylamide hydrochloride913-04-2 | 404.2 | 388.2 | 7.70 | 80 | 15 |
| 17-phenyl trinor Prostaglandin F2α; cyclopropyl amide | 428.3 | 353.2 | 7.77 | 80 | 15 |
| Mycalamide A | 504.3 | 329.2 | 7.77 | 80 | 15 |
| C17-Sphinganine | 288.3 | 270.3 | 7.80 | 80 | 30 |
| 5a-Androstan-17β-ol-3-one | 291.2 | 230.1 | 7.81 | 80 | 15 |
| 3-hydroxy-C12 homoserine lactone | 300.2 | 283.2 | 7.87 | 80 | 15 |
| 3-(1,2,3,4-Tetrahydro-6-hydroxy-2-naphthyl)cyclopentanone | 231.1 | 191.1 | 7.93 | 80 | 30 |
| DAG 16:1 | 360.3 | 283.2 | 8.03 | 80 | 15 |
| 2-trans,4-cis-Decadienoylcarnitine | 312.2 | 310.2 | 8.06 | 80 | 15 |
| 24-Nor-5β-cholane-3α,7α,22,23-tetrol | 381.3 | 364.3 | 8.08 | 80 | 15 |
| Taurolithocholate | 484.3 | 437.2 | 8.17 | 80 | 15 |
| 3β-Hydroxypregn-5-en-20-one sulfate | 397.2 | 387.8 | 8.29 | 80 | 15 |
| Tetrahydrogeranylgeranyl diphosphate | 455.2 | 453.2 | 8.31 | 80 | 15 |
| MEGA | 326.2 | 284.3 | 8.33 | 80 | 15 |
| 2,6 Dimethylheptanoyl carnitine | 302.2 | 244.2 | 8.35 | 80 | 30 |
| 2-Hydroxylauroylcarnitine | 360.3 | 358.2 | 8.36 | 80 | 15 |
| PC(0:0/5:0) | 342.2 | 258.1 | 8.41 | 80 | 45 |
| Lauroyl-EA | 244.2 | 227.2 | 8.42 | 80 | 30 |
| 5β-Cholanic acid | 361.3 | 276.2 | 8.47 | 80 | 30 |
| Erucic acid | 361.3 | 359.3 | 8.47 | 80 | 15 |
| N-Glycosyl-L-asparagine | 295.1 | 256.3 | 8.52 | 80 | 30 |
| 28-Homobrassinolide | 495.4 | 70.0 | 8.62 | 80 | 15 |
| DG 24:1 | 472.4 | 391.3 | 8.62 | 80 | 15 |
| N-dodecanoyl-L-Homoserine lactone | 284.2 | 224.2 | 8.63 | 80 | 15 |
| [6]-Gingerdiol 3,5-diacetate | 398.3 | 383.3 | 8.65 | 80 | 15 |
| DAG 18:2 | 386.3 | 384.3 | 8.87 | 80 | 15 |
| 2-amino-tetradecanoic acid | 244.2 | 226.2 | 8.88 | 80 | 30 |
| Prostaglandin E2 serinol amide | 426.3 | 315.2 | 8.94 | 80 | 15 |
| N-palmitoyl glutamic acid | 386.3 | 384.3 | 9.00 | 80 | 15 |
| N-palmitoyl serine | 344.3 | 175.1 | 9.01 | 80 | 45 |
| 1-Arachidonoylglycerol | 379.3 | 86.1 | 9.09 | 80 | 45 |
| LysoPE(18:1(9Z)/0:0) | 480.3 | 340.3 | 9.23 | 80 | 30 |
| O-Decanoyl-L-carnitine | 316.2 | 144.1 | 9.24 | 80 | 45 |
| N-oleoyl glutamic acid | 412.3 | 85.0 | 9.28 | 80 | 30 |
| Mesterolone | 305.2 | 243.2 | 9.29 | 80 | 30 |
| Pregnenolone | 317.3 | 315.2 | 9.35 | 80 | 15 |
| DAG 18:4 | 382.3 | 271.2 | 9.50 | 80 | 15 |
| Arachidonic acid | 305.2 | 244.2 | 9.52 | 80 | 15 |
| Dl-Dihydrosphingosine | 302.3 | 284.3 | 9.53 | 80 | 45 |
| PGF2α-dihydroxypropanylamine | 428.3 | 316.2 | 9.54 | 80 | 15 |
| cis-5-Tetradecenoylcarnitine | 370.3 | 311.2 | 9.55 | 80 | 30 |
| 7-Ethoxy-4-methyl-2h-chromen-2-one | 205.1 | 188.1 | 9.57 | 80 | 15 |
| 7,10,13,16-Docosatetraenoylethanolamine | 375.3 | 233.2 | 9.61 | 80 | 45 |
| (9E)-10-nitrooctadecenoic Acid | 328.2 | 282.9 | 9.62 | 80 | 15 |
| PE 21:2 | 534.3 | 89.1 | 9.74 | 80 | 45 |
| PE(12:0/16:1(9Z)) | 634.4 | 617.4 | 9.79 | 80 | 15 |
| 2-O-(β-D-galactopyranosyl-(1->6)-β-D-galactopyranosyl) 2S-hydroxytridecanoic acid | 555.3 | 540.4 | 10.01 | 80 | 15 |
| 9-Nitrooleate | 328.2 | 283.3 | 10.01 | 80 | 15 |
| L-Hexanoylcarnitine n-butyl ester | 316.2 | 314.2 | 10.05 | 80 | 15 |
| MG 16:4 | 340.2 | 322.2 | 10.11 | 80 | 15 |
| SM(d18:1/16:0) | 703.6 | 185.1 | 10.18 | 80 | 30 |
| PC(O-18:0/1:0) | 538.4 | 520.4 | 10.21 | 80 | 15 |
| PC(19:0/0:0) | 538.4 | 282.3 | 10.21 | 80 | 30 |
| Arachidonoyl Ethanolamide-d4 | 352.3 | 336.3 | 10.23 | 80 | 15 |
| 3-hydroxy-C14 homoserine lactone | 328.2 | 282.9 | 10.24 | 80 | 15 |
| Hexadecanamide | 256.3 | 227.1 | 10.29 | 80 | 45 |
| PG(18:3(6Z,9Z,12Z)/0:0) | 507.3 | 134.1 | 10.38 | 80 | 15 |
| Eicosanoyl-EA | 356.4 | 121.1 | 10.40 | 80 | 45 |
| Oleamide | 282.3 | 265.3 | 10.51 | 80 | 30 |
| 10-Nitrooleate | 328.2 | 282.9 | 10.52 | 80 | 15 |
| α-Spinasterol | 395.4 | 393.3 | 10.60 | 80 | 15 |
| Stigmasterol | 395.4 | 377.3 | 10.60 | 80 | 30 |
| Anandamide (20:l, n-9) | 354.3 | 352.3 | 10.61 | 80 | 15 |
| PGF2α; dimethyl amine | 368.3 | 228.2 | 10.81 | 80 | 15 |
| dehydro-β-Ionone | 191.1 | 175.1 | 10.82 | 80 | 30 |
| 2-(Octylthio)ethanol | 191.1 | 175.1 | 10.91 | 80 | 15 |
| N-octadecanoyl-L-Homoserine lactone | 368.3 | 228.2 | 10.91 | 80 | 15 |
| 4,8 Dimethylnonanoyl carnitine | 330.3 | 272.2 | 10.92 | 80 | 30 |
| Stearamide | 284.3 | 282.3 | 10.95 | 80 | 15 |
| Glucosylisomaltol | 289.1 | 127.0 | 10.97 | 80 | 45 |
| 13E-Docosenamide | 338.3 | 322.2 | 11.00 | 80 | 30 |
| 24,25-Dihydrolanosterol | 429.4 | 344.3 | 11.00 | 80 | 45 |
| Palmitoylethanolamide | 300.3 | 95.1 | 11.01 | 80 | 45 |
| Leucyl-leucyl-norleucine | 358.3 | 313.3 | 11.02 | 80 | 45 |
| Oleoyl Ethyl Amide | 310.3 | 294.3 | 11.08 | 80 | 15 |
| 14-Methylhexadecanoic Acid | 271.3 | 241.2 | 11.10 | 80 | 45 |
| Sphingosine | 300.3 | 95.1 | 11.10 | 80 | 45 |
| SM d18:1/12:0 | 647.5 | 185.1 | 11.39 | 80 | 15 |
| 3-Ethylpyridine | 108.1 | 93.1 | 11.41 | 80 | 45 |
| Tricosanoylglycine | 412.4 | 109.1 | 11.47 | 80 | 30 |
| Hexadecanoic acid | 274.3 | 257.3 | 11.57 | 80 | 45 |
| Palmitoleamide | 254.2 | 133.1 | 11.59 | 80 | 30 |
| 3-Dehydrosphinganine | 300.3 | 283.3 | 11.63 | 80 | 30 |
| N-Hexadecanoylpyrrolidine | 310.3 | 293.3 | 11.72 | 80 | 15 |
| Heptadecanoic acid | 271.3 | 254.2 | 11.80 | 80 | 30 |
| PC 34:2 | 758.6 | 185.1 | 11.82 | 80 | 30 |
| PE 35:2 | 752.5 | 736.5 | 11.96 | 80 | 15 |
| PG 40:4 | 844.6 | 520.5 | 12.08 | 80 | 15 |
| Lactosylceramide(18:1/16:0) | 862.6 | 283.3 | 12.08 | 80 | 45 |
| PC(22:2(13Z,16Z)/12:0) | 758.6 | 185.1 | 12.08 | 80 | 45 |
| HexCer-BS d34:1 | 716.6 | 282.3 | 12.16 | 80 | 45 |
| 2-[3,4-bis[[(2S,3R,4S,5S,6R)-3,4,5-trihydroxy-6-(hydroxymethyl)oxan-2-yl]oxy]phenyl]-5,7-dihydroxychromen-4-one | 628.2 | 359.0 | 12.22 | 80 | 45 |
| 5β-Cholestan-3-one | 369.3 | 367.3 | 12.27 | 80 | 15 |
| HexCer-NS d34:1 | 700.6 | 282.3 | 12.30 | 80 | 45 |
| 4-Hydroxy-16,18-tritriacontanedione | 509.5 | 491.5 | 12.58 | 80 | 15 |
| 1,2-Dierucoyl-SN-Glycero-3-Phosphoethanolamine | 732.6 | 299.3 | 12.60 | 80 | 45 |
| Cer-BS d34:1 | 554.5 | 282.3 | 12.60 | 80 | 45 |
| MGDG 31:1 | 732.6 | 536.5 | 12.60 | 80 | 30 |
| Hexadecasphinganine | 274.3 | 256.3 | 12.60 | 80 | 30 |
| PA(22:0/12:0) | 677.5 | 89.1 | 12.61 | 80 | 30 |
| 13'-Carboxy-γ-tocopherol | 447.3 | 307.2 | 12.61 | 80 | 15 |
| Cer 33:1 | 524.5 | 507.5 | 12.66 | 80 | 15 |
| GlcADG 32:1 | 760.6 | 562.5 | 12.66 | 80 | 15 |
| PC(14:0/20:2(11Z,14Z)) | 758.6 | 325.3 | 12.67 | 80 | 30 |
| HexCer-AP t38:0 | 790.6 | 629.6 | 12.67 | 80 | 15 |
| GlcADG 34:2; GlcADG 16:1-18:1 | 786.6 | 593.5 | 12.70 | 80 | 15 |
| MGDG 32:1 | 746.6 | 550.5 | 12.76 | 80 | 15 |
| Tetrahydro-6-(2-hydroxy-16,19-dimethylhexacosyl)-4-methyl-2H-pyran-2-one | 523.5 | 299.3 | 12.76 | 80 | 15 |
| DG(17:2(9Z,12Z)/18:1(9Z)/0:0)[iso2] | 605.5 | 537.5 | 12.77 | 80 | 15 |
| Ceramide (18:1/16:0) | 538.5 | 522.5 | 12.83 | 80 | 15 |
| MGDG 34:2 | 772.6 | 593.5 | 12.83 | 80 | 15 |
| N-Palmitoylsphingosine | 538.5 | 284.3 | 12.83 | 80 | 45 |
| GlcADG 33:1 | 774.6 | 576.5 | 12.86 | 80 | 15 |
| Cer-AP t34:2 | 568.5 | 345.3 | 12.87 | 80 | 15 |
| GlcCer(d16:1(4E)/22:0(2OH)) | 772.6 | 755.6 | 12.89 | 80 | 15 |
| GlcADG 35:2; GlcADG 17:1-18:1 | 800.6 | 590.5 | 12.90 | 80 | 15 |
| Pantothenol | 206.1 | 166.1 | 12.93 | 80 | 15 |
| Cer-NDS d37:0 | 582.6 | 543.5 | 12.95 | 80 | 30 |
| GlcADG 34:1 | 788.6 | 708.5 | 12.96 | 80 | 15 |
| MGDG 33:1 | 760.6 | 564.5 | 12.98 | 80 | 30 |
| 1-Hexadecanoyl-2-(9Z-octadecenoyl)-sn-glycero-3-phosphocholine | 760.6 | 564.5 | 12.98 | 80 | 15 |
| GlcADG 36:2 | 814.6 | 621.5 | 12.99 | 80 | 15 |
| MGDG 35:2 | 786.6 | 607.5 | 13.04 | 80 | 15 |
| MGDG 34:1 | 774.6 | 578.5 | 13.09 | 80 | 15 |
| HexCer-AP t41:0 | 832.7 | 653.6 | 13.22 | 80 | 30 |
| HexCer-BDS d40:1 | 800.7 | 783.6 | 13.25 | 80 | 15 |
| HexCer-BS d40:1 | 800.7 | 353.3 | 13.25 | 80 | 30 |
| DG(20:0/18:3(6Z,9Z,12Z)/0:0) | 647.6 | 391.3 | 13.34 | 80 | 15 |
| Glucosylceramide (d18:1/22:0) | 784.7 | 767.7 | 13.37 | 80 | 15 |
| Glucosylceramide (d18:1/24:1(15Z)) | 810.7 | 794.7 | 13.40 | 80 | 15 |
| DG(22:1(13Z)/18:3(6Z,9Z,12Z)/0:0) | 673.6 | 537.5 | 13.41 | 80 | 15 |
| HexCer-AS d41:1 | 814.7 | 282.3 | 13.44 | 80 | 30 |
| HexCer-BDS d42:0 | 830.7 | 670.7 | 13.51 | 80 | 15 |
| HexCer-NP t42:0 | 830.7 | 651.6 | 13.51 | 80 | 30 |
| Nervonyl carnitine | 102.1 | 74.1 | 13.53 | 80 | 15 |
| HexCer-BS d42:1 | 828.7 | 811.7 | 13.63 | 80 | 15 |
| Cer-AP t42:0 | 684.6 | 667.6 | 13.82 | 80 | 15 |
| Erucamide | 338.3 | 322.3 | 13.86 | 80 | 15 |
| PG(12:0/20:1(11Z)) | 721.5 | 664.5 | 13.96 | 80 | 15 |
| Cer(d18:1/24:1(15Z)) | 648.6 | 265.3 | 14.00 | 80 | 45 |
| Ceramide (d18:1/26:1(17Z)) | 676.7 | 298.3 | 14.07 | 80 | 30 |
| Cer(t18:0/24:0) | 668.7 | 651.6 | 14.08 | 80 | 15 |
| (4'R,6R,6'S,7S,8R)-6'-(2-hydroxypentan-2-yl)-4',8-dimethyldecahydro-5H-spiro[indolizine-6,2'-pyran]-7,8-diol | 342.3 | 327.2 | 14.19 | 80 | 15 |
| 15-hydroxy stearic acid | 301.3 | 284.2 | 14.20 | 80 | 15 |
| Cer(t18:0/26:0(2-OH)) | 712.7 | 310.3 | 14.24 | 80 | 30 |
| MAG 16:3 | 342.3 | 327.2 | 14.30 | 80 | 15 |
| 14-hydroxy stearic acid | 301.3 | 284.2 | 14.32 | 80 | 15 |
| MG 16:3 | 342.3 | 327.2 | 14.42 | 80 | 15 |
| Ceramide (d18:1/24:0) | 650.6 | 633.6 | 14.44 | 80 | 15 |
| DG(22:4(7Z,10Z,13Z,16Z)/20:1(11Z)/0:0) | 699.6 | 105.0 | 14.48 | 80 | 45 |
| (R)-10-Hydroxystearate | 301.3 | 284.2 | 14.48 | 80 | 15 |
| DG(20:1(11Z)/22:4(7Z,10Z,13Z,16Z)/0:0)[iso2] | 699.6 | 105.0 | 14.49 | 80 | 30 |
| (R)-2-Hydroxystearate | 301.3 | 284.2 | 14.49 | 80 | 15 |
| 5E,7E-Dodecadienyl acetate | 225.2 | 186.0 | 14.50 | 80 | 45 |
| PA(20:1(11Z)/12:0) | 647.5 | 351.3 | 14.54 | 80 | 15 |
| PA(13:0/19:1(9Z)) | 647.5 | 591.4 | 14.54 | 80 | 30 |
| cannitine C8:0-d3 | 291.2 | 230.1 | 7.69 | 80 | 30 |
| L-Phenylalanine-d5 | 171.1 | 169.1 | 1.81 | 80 | 30 |

DP: declustering potential; CE:collision energy.

2 Urine negative ions

| Name | Q1(Da) | Q3(Da) | Expected RT (min) | DP (eV) | CE(eV) |
| --- | --- | --- | --- | --- | --- |
| Cis-Aconitic acid | 173.0 | 130.0 | 1.05 | -80 | -30 |
| Citric acid | 191.0 | 173.0 | 1.05 | -80 | -30 |
| D-2-Aminoadipic acid | 160.1 | 142.0 | 1.05 | -80 | -30 |
| Pyroglutamic acid | 128.0 | 101.0 | 1.05 | -80 | -30 |
| Uric acid | 167.0 | 142.0 | 1.05 | -80 | -30 |
| 3-Furoic acid | 111.0 | 82.0 | 1.06 | -80 | -45 |
| hydroxybutyrylglycine | 160.1 | 116.0 | 1.06 | -80 | -45 |
| Vanilpyruvic acid | 209.1 | 174.1 | 1.06 | -80 | -15 |
| 2',3'-Cyclic AMP | 328.0 | 167.0 | 1.07 | -80 | -45 |
| Itaconic acid | 129.0 | 126.9 | 1.07 | -80 | -30 |
| N-Acetyl-Dl-glutamic acid | 188.1 | 175.0 | 1.07 | -80 | -30 |
| 2,3-Dihydroxyisovaleric Acid | 133.0 | 131.0 | 1.08 | -80 | -30 |
| L-Gulono-1,4-lactone | 178.0 | 161.9 | 1.08 | -80 | -45 |
| N-Acetylaspartylglutamate | 303.1 | 285.1 | 1.08 | -80 | -30 |
| O-Phospho-L-Tyrosine | 260.0 | 243.0 | 1.08 | -80 | -15 |
| Ascorbic acid-2-sulfate | 255.0 | 219.8 | 1.09 | -80 | -45 |
| Flavanone | 223.0 | 203.9 | 1.09 | -80 | -30 |
| 3,4-Dihydroxyphenylglycol O-sulfate | 249.0 | 191.0 | 1.11 | -80 | -30 |
| Purine | 119.0 | 117.0 | 1.11 | -80 | -30 |
| D-Citramalic acid | 147.0 | 145.0 | 1.15 | -80 | -30 |
| 5-Hydroxy-thiabendazole | 216.0 | 200.1 | 1.16 | -80 | -15 |
| N-Acetylgalactosamine 4-sulfate | 300.0 | 240.1 | 1.19 | -80 | -15 |
| Citraconic acid | 129.0 | 126.9 | 1.21 | -80 | -30 |
| 3-(4-Hydroxyphenyl)pyruvate | 180.1 | 163.0 | 1.23 | -80 | -15 |
| D-Glucono-1,5-lactone | 178.0 | 161.9 | 1.23 | -80 | -45 |
| Guanosine | 283.1 | 248.8 | 1.24 | -80 | -30 |
| Nicotinurate | 180.1 | 178.0 | 1.24 | -80 | -30 |
| 3-Hydroxy-3-Methylpentanedioic acid | 161.0 | 143.0 | 1.25 | -80 | -30 |
| Adenosine5'-diphosphoglucose | 588.1 | 319.1 | 1.25 | -80 | -30 |
| N2-Succinyl-L-ornithine | 231.1 | 214.1 | 1.25 | -80 | -15 |
| Guanosine diphosphate fucose | 588.1 | 260.0 | 1.26 | -80 | -45 |
| 1-Deoxy-D-xylulose | 133.0 | 131.0 | 1.28 | -80 | -30 |
| 4-Pyridoxic acid | 182.0 | 163.0 | 1.28 | -80 | -30 |
| Isocitric acid | 191.0 | 189.0 | 1.28 | -80 | -30 |
| N-Methyl-L-glutamic acid | 142.0 | 102.1 | 1.29 | -80 | -30 |
| 4-Hydroxy-2-oxobutanoic acid | 117.0 | 99.9 | 1.31 | -80 | -30 |
| Xanthosine | 283.1 | 248.8 | 1.32 | -80 | -30 |
| 4-Acetamidobutanoate | 144.1 | 142.0 | 1.33 | -80 | -45 |
| 2,4-Dinitrotoluene | 181.0 | 166.0 | 1.34 | -80 | -15 |
| 7-Methyluric acid | 181.0 | 166.0 | 1.35 | -80 | -15 |
| 1-Methyluric Acid | 181.0 | 163.0 | 1.36 | -80 | -30 |
| Homogentisic acid | 167.0 | 151.0 | 1.37 | -80 | -30 |
| 1-Methylguanosine | 296.1 | 280.1 | 1.38 | -80 | -15 |
| Methazolamide | 235.0 | 220.0 | 1.38 | -80 | -15 |
| 3'-O-Methylguanosine | 296.1 | 208.1 | 1.41 | -80 | -30 |
| 6-O-p-Coumaroyl-1,2-digalloylglucose | 629.1 | 219.0 | 1.41 | -80 | -45 |
| β-Hydroxyisobutyrate | 103.0 | 85.0 | 1.43 | -80 | -30 |
| Isoniazid α-ketoglutaric acid | 264.1 | 245.1 | 1.46 | -80 | -15 |
| Glycitein | 284.1 | 247.8 | 1.49 | -80 | -45 |
| D-2-Hydroxypentanedioic acid | 129.0 | 126.9 | 1.50 | -80 | -15 |
| Acetaminophen glucuronide | 653.2 | 327.1 | 1.51 | -80 | -30 |
| IDP | 427.0 | 152.0 | 1.51 | -80 | -45 |
| 1-Methylxanthine | 165.0 | 162.8 | 1.52 | -80 | -30 |
| 7-Methylxanthine | 165.0 | 162.8 | 1.53 | -80 | -45 |
| 2-Methylguanosine | 296.1 | 280.1 | 1.56 | -80 | -15 |
| (S)-2-Acetolactate | 131.0 | 113.0 | 1.59 | -80 | -15 |
| Urate-3-ribonucleoside | 299.1 | 219.0 | 1.60 | -80 | -45 |
| 7-Methylguanosine | 296.1 | 233.1 | 1.61 | -80 | -30 |
| Citicoline | 509.1 | 219.0 | 1.61 | -80 | -30 |
| Tetrahydropentoxyline | 365.1 | 347.1 | 1.62 | -80 | -15 |
| 5-hydroxyhexanoylglycine | 188.1 | 174.0 | 1.63 | -80 | -15 |
| Glutamylvaline | 245.1 | 228.1 | 1.64 | -80 | -15 |
| 1-(5-Phosphoribosyl)imidazole-4-acetate | 337.0 | 219.0 | 1.65 | -80 | -30 |
| (-)-3-(3,4-Dihydroxyphenyl)-2-methylalanine | 210.1 | 191.0 | 1.66 | -80 | -15 |
| Dl-4-Hydroxy-3-methoxymandelie acid | 197.0 | 162.8 | 1.66 | -80 | -15 |
| Nicotinamide-N-oxide | 137.0 | 110.9 | 1.66 | -80 | -15 |
| 2-Oxo-3-hydroxy-4-phosphobutanoate | 213.0 | 170.0 | 1.67 | -80 | -15 |
| N4-Acetylcytidine | 284.1 | 248.8 | 1.67 | -80 | -15 |
| 7,8-dihydroxy-2H-chromen-2-one | 177.0 | 159.9 | 1.68 | -80 | -15 |
| 2-Hydroxy-6-methylpyridin-3-carboxylic acid | 152.0 | 109.0 | 1.70 | -80 | -15 |
| Acesulfame | 162.0 | 147.0 | 1.74 | -80 | -30 |
| 4-Hydroxy-3-methoxymandelic acid | 197.0 | 163.8 | 1.75 | -80 | -30 |
| 3-(Cystein-S-yl)acetaminophen | 269.1 | 221.8 | 1.77 | -80 | -30 |
| Aspartyl-Tyrosine | 295.1 | 277.1 | 1.83 | -80 | -15 |
| 3-(3,4-Dihydroxyphenyl)-2-methyl-L-alanine | 210.1 | 167.1 | 1.84 | -80 | -30 |
| 5-Hydroxy-7-methoxy-6-methylflavone | 281.1 | 263.1 | 1.84 | -80 | -15 |
| Acesulfame-K | 162.0 | 147.0 | 1.84 | -80 | -15 |
| Esculetin | 177.0 | 159.9 | 1.86 | -80 | -15 |
| 5-ethyl-5-methyl-2,4-oxazolidinedione | 142.0 | 126.1 | 1.90 | -80 | -30 |
| 2-Hydroxyacetaminophen sulfate | 246.0 | 185.9 | 1.92 | -80 | -45 |
| 5-Hydroxypentanoate | 117.1 | 99.9 | 1.95 | -80 | -30 |
| 2'-Deoxyinosine | 251.1 | 223.1 | 2.00 | -80 | -30 |
| N-Isobutyrylglycine | 144.1 | 142.0 | 2.02 | -80 | -30 |
| 3-Methoxy-4-hydroxyphenylethyleneglycol sulfate | 263.0 | 223.8 | 2.03 | -80 | -45 |
| 4-Oxopentanoate | 115.0 | 113.0 | 2.11 | -80 | -30 |
| p-Acetamidophenyl-β-D-glucuronide | 326.1 | 230.0 | 2.11 | -80 | -45 |
| 2'-Hydroxyacetanilide | 150.1 | 123.0 | 2.12 | -80 | -30 |
| 2,5-Dimethyl-3-(methyldithio)furan | 173.0 | 147.0 | 2.20 | -80 | -15 |
| Glutaric acid | 131.0 | 113.0 | 2.20 | -80 | -30 |
| N-Isovaleroylglycine | 158.1 | 140.1 | 2.20 | -80 | -30 |
| Cysteinyl-γ-glutamate | 248.1 | 204.1 | 2.24 | -80 | -15 |
| Porphobilinogen | 225.1 | 188.9 | 2.28 | -80 | -15 |
| L-2,3-Dihydrodipicolinate | 168.0 | 132.9 | 2.30 | -80 | -15 |
| Formononetin | 268.1 | 250.1 | 2.34 | -80 | -15 |
| (2-Methoxyethoxy)propanoic acid | 147.1 | 145.0 | 2.35 | -80 | -15 |
| (+)-Norushinsunine N-oxide | 310.1 | 308.1 | 2.38 | -80 | -15 |
| 3-hydroxy-3-(3-hydroxyphenyl)propanoic acid-O-sulphate | 261.0 | 223.8 | 2.39 | -80 | -15 |
| Nicotinamide | 121.0 | 62.0 | 2.40 | -80 | -30 |
| N-(2-Furoyl)glycine | 168.0 | 132.9 | 2.42 | -80 | -15 |
| Cysteinyl-Glutamine | 248.1 | 204.1 | 2.44 | -80 | -15 |
| 2-METHOXYRESORCINOL | 139.0 | 137.0 | 2.46 | -80 | -30 |
| 2-Aminoethylphosphonic acid | 124.0 | 95.0 | 2.47 | -80 | -45 |
| 6-(Methylsulfonyl)hexyl glucosinolate | 480.1 | 219.0 | 2.48 | -80 | -45 |
| 2-Pyridinemethanethiol | 124.0 | 95.0 | 2.50 | -80 | -15 |
| 8-Oxoguanine | 167.0 | 132.9 | 2.50 | -80 | -45 |
| Quercetin 3-xylosyl-(1->6)-glucoside | 595.1 | 219.0 | 2.50 | -80 | -45 |
| Vanillic acid | 167.0 | 124.1 | 2.50 | -80 | -30 |
| 2-Methylsuccinic acid | 131.0 | 97.0 | 2.51 | -80 | -15 |
| Dl-Cystine | 261.0 | 223.8 | 2.54 | -80 | -15 |
| 1,7-Dimethylguanosine | 310.1 | 179.1 | 2.62 | -80 | -45 |
| Methylgallic acid-O-sulphate | 263.0 | 223.8 | 2.67 | -80 | -30 |
| N-[4'-hydroxy-(E)-cinnamoyl]-L-aspartic acid | 278.1 | 250.9 | 2.74 | -80 | -30 |
| Nicotinamide riboside | 253.1 | 235.1 | 2.76 | -80 | -15 |
| Methyl 2-methyllactate | 117.1 | 100.9 | 2.77 | -80 | -30 |
| D-Arabinose | 150.1 | 123.0 | 2.79 | -80 | -15 |
| 3,4-Dihydroxybenzaldehyde | 137.0 | 108.9 | 2.83 | -80 | -15 |
| 6-(2-Hydroxyethoxy)-6-oxohexanoic acid | 189.1 | 171.1 | 2.85 | -80 | -30 |
| Indoxyl glucuronide | 308.1 | 178.1 | 2.87 | -80 | -45 |
| METHYL (-)-SHIKIMATE; Methyl 3,4,5-trihydroxy-1-cyclohexene-1-carboxylate | 187.1 | 159.9 | 2.93 | -80 | -45 |
| Pyrraline | 253.1 | 223.1 | 2.94 | -80 | -30 |
| 3-oxo-C4-homoserine lactone | 184.1 | 140.9 | 2.95 | -80 | -15 |
| Peonidin 3-rhamnoside | 446.1 | 428.1 | 2.95 | -80 | -15 |
| 4-Hydroxybenzyl isothiocyanate 4''-acetylrhamnoside | 352.1 | 334.1 | 3.07 | -80 | -15 |
| Biochanin A-β-D-glucoside | 446.1 | 428.1 | 3.10 | -80 | -15 |
| Thiamin monophosphate | 343.1 | 247.0 | 3.12 | -80 | -30 |
| 4-Hydroxybenzoic acid | 137.0 | 108.9 | 3.13 | -80 | -15 |
| 2-Methylglutaric acid | 145.0 | 127.0 | 3.17 | -80 | -45 |
| Dl-Glyceraldehyde3-phosphate | 169.0 | 126.9 | 3.17 | -80 | -45 |
| 2,3-Dimethylsuccinic Acid | 145.0 | 127.0 | 3.18 | -80 | -15 |
| 6-Methoxy-pyridine-3-carboxylic acid | 152.0 | 109.0 | 3.20 | -80 | -15 |
| Vanillic acid 4-sulfate | 247.0 | 203.0 | 3.20 | -80 | -30 |
| N6-Succinyl adenosine | 382.1 | 301.1 | 3.21 | -80 | -30 |
| Digalacturonate | 370.1 | 235.1 | 3.23 | -80 | -45 |
| p-Aminohippuric acid | 193.1 | 191.0 | 3.23 | -80 | -15 |
| 6-Benzyladenine | 224.1 | 180.1 | 3.34 | -80 | -15 |
| Thymidine 5-monophosphate | 321.1 | 179.0 | 3.34 | -80 | -45 |
| 4-methoxy-3-(sulfooxy)benzoic acid | 247.0 | 228.1 | 3.36 | -80 | -15 |
| (S)-(-)-Acenocoumarol | 352.1 | 334.1 | 3.37 | -80 | -15 |
| 3-Methylcatechol | 123.0 | 121.0 | 3.37 | -80 | -45 |
| D-Xylulose | 150.1 | 136.0 | 3.37 | -80 | -15 |
| Hydroquinone | 109.0 | 66.0 | 3.37 | -80 | -15 |
| Pantoyllactone glucoside | 291.1 | 253.1 | 3.37 | -80 | -15 |
| 3-Hydroxybenzyl alcohol | 123.0 | 121.0 | 3.38 | -80 | -15 |
| n-Propyl gallate | 211.1 | 169.0 | 3.38 | -80 | -15 |
| Vanillin | 151.0 | 149.0 | 3.38 | -80 | -15 |
| Gentisate aldehyde | 137.0 | 108.0 | 3.39 | -80 | -15 |
| 3-Phenylpropyl glucosinolate | 436.1 | 375.1 | 3.42 | -80 | -30 |
| 2'-Deoxyguanosine 5'-Monophosphate | 346.1 | 344.0 | 3.49 | -80 | -15 |
| 2,3-Dimethylmaleate | 143.0 | 127.0 | 3.51 | -80 | -15 |
| Pantetheine 4'-phosphate | 357.1 | 182.0 | 3.51 | -80 | -45 |
| γ-Glu-Leu | 259.1 | 221.8 | 3.51 | -80 | -45 |
| Riboflavin B2 | 375.1 | 329.1 | 3.52 | -80 | -15 |
| 2-Isopropylmalic acid | 157.0 | 139.0 | 3.53 | -80 | -15 |
| Pyrogallol-2-O-sulphate | 205.0 | 126.0 | 3.53 | -80 | -30 |
| (-)-Gallocatechin | 305.1 | 226.1 | 3.54 | -80 | -45 |
| Glycylprolylhydroxyproline | 284.1 | 183.9 | 3.55 | -80 | -45 |
| D-Glucosamine-6-phosphate | 258.0 | 241.1 | 3.56 | -80 | -15 |
| Epigallocatechin | 305.1 | 269.9 | 3.56 | -80 | -15 |
| N-γ-Glutamyl-S-propylcysteine | 291.1 | 273.1 | 3.57 | -80 | -30 |
| Rhamnazin 3-sophoroside | 653.2 | 355.1 | 3.59 | -80 | -15 |
| 5,7-Dihydroxychromone | 177.0 | 147.0 | 3.60 | -80 | -15 |
| Benzoic acid | 121.0 | 95.0 | 3.60 | -80 | -15 |
| Dl-P-Hydroxyphenyl lactic acid | 363.1 | 301.1 | 3.60 | -80 | -15 |
| Homovanillic acid sulfate | 261.0 | 242.1 | 3.60 | -80 | -15 |
| Phaseolic acid | 295.0 | 173.0 | 3.60 | -80 | -15 |
| Tetrahydro-2,5-furan-diacetic acid | 187.1 | 144.1 | 3.65 | -80 | -15 |
| Vanilloylglycine | 224.1 | 181.1 | 3.65 | -80 | -15 |
| Tetrahydrofurfuryl acetate | 143.1 | 127.0 | 3.67 | -80 | -15 |
| L-β-aspartyl-L-phenylalanine | 279.1 | 263.1 | 3.70 | -80 | -15 |
| Flavone | 222.1 | 220.0 | 3.71 | -80 | -15 |
| 5-Acetamidopentanoate | 158.1 | 156.1 | 3.72 | -80 | -30 |
| Cyclohexylsulfamate | 178.1 | 136.0 | 3.73 | -80 | -15 |
| Valerylglycine | 158.1 | 156.1 | 3.73 | -80 | -15 |
| 16-Ketoestradiol | 285.1 | 267.1 | 3.74 | -80 | -15 |
| 2-(4-Hydroxyphenl)-Ethonol | 137.1 | 95.0 | 3.75 | -80 | -15 |
| 2-Methyl-3-hydroxy-5-formylpyridine-4-carboxylate | 180.0 | 137.1 | 3.79 | -80 | -15 |
| Salicylamide (JAN/USP/INN) | 136.0 | 134.0 | 3.79 | -80 | -45 |
| 3-(4-Hydroxyphenyl)-propionic acid | 165.1 | 136.9 | 3.81 | -80 | -15 |
| 4-Hydroxyquinoline | 144.0 | 127.0 | 3.82 | -80 | -15 |
| Asp-phe | 279.1 | 261.1 | 3.84 | -80 | -30 |
| 2-Protocatechoylphloroglucinolcarboxylate | 305.0 | 225.1 | 3.85 | -80 | -15 |
| 1-Salicylate glucuronide | 313.1 | 295.0 | 3.86 | -80 | -30 |
| 5-Hydroxymethyluridine | 274.1 | 231.1 | 3.86 | -80 | -30 |
| Dihyroxy-1H-indole glucuronide I | 324.1 | 236.0 | 3.87 | -80 | -45 |
| 4-Acetamidophenol | 150.1 | 136.0 | 3.89 | -80 | -15 |
| 3-Dehydroshikimic acid | 153.0 | 137.0 | 3.90 | -80 | -15 |
| 3-Hydroxybenzaldehyde | 121.0 | 95.0 | 3.93 | -80 | -30 |
| Homovanillic acid | 181.0 | 168.0 | 3.93 | -80 | -45 |
| 3-Amino-3-(4-hydroxyphenyl)-propionic acid | 361.1 | 359.1 | 3.94 | -80 | -15 |
| Vanillin 4-sulfate | 231.0 | 153.1 | 3.94 | -80 | -45 |
| Phenyl glucuronide | 269.1 | 173.0 | 3.98 | -80 | -45 |
| γ-Glutamylphenylalanine | 293.1 | 223.1 | 3.98 | -80 | -45 |
| (±)-3-Methyl-2-oxovaleric acid | 129.1 | 126.9 | 3.99 | -80 | -45 |
| N-lactoyl-Tyrosine | 252.1 | 223.1 | 4.00 | -80 | -30 |
| 3-Indoxylsulfate potassium salt | 212.0 | 169.0 | 4.09 | -80 | -45 |
| 4,7-Dihydroxy-2H-1-benzopyran-2-one | 177.0 | 175.0 | 4.09 | -80 | -15 |
| Dihydrocaffeic acid 3-sulfate | 261.0 | 217.0 | 4.09 | -80 | -30 |
| 3-Hydroxyhippuric acid | 194.0 | 151.1 | 4.11 | -80 | -15 |
| Dopaxanthin | 389.1 | 195.0 | 4.11 | -80 | -45 |
| Glycylglycine | 132.0 | 118.0 | 4.11 | -80 | -15 |
| Pyridoxine | 150.1 | 136.0 | 4.12 | -80 | -45 |
| Guanosine 5'-diphosphate | 442.0 | 398.0 | 4.14 | -80 | -15 |
| 6-Phosphogluconic acid | 275.0 | 196.1 | 4.15 | -80 | -15 |
| (S)-(+)-Mandelic acid | 151.0 | 149.0 | 4.18 | -80 | -45 |
| 5-Hydroxy-N-formylkynurenine | 251.1 | 233.1 | 4.22 | -80 | -15 |
| 4-O-α-L-Rhamnopyranosylglucosinalbin | 570.1 | 213.0 | 4.23 | -80 | -45 |
| Dihydrocaffeic acid 3-O-glucuronide | 357.1 | 320.9 | 4.23 | -80 | -30 |
| Isoferulic acid 3-sulfate | 273.0 | 229.0 | 4.23 | -80 | -15 |
| 2-Ethylglutaric acid | 159.1 | 141.1 | 4.24 | -80 | -15 |
| Albendazole-2-aminosulfone | 238.1 | 195.1 | 4.27 | -80 | -15 |
| Ethylmalonic acid | 132.0 | 118.0 | 4.27 | -80 | -45 |
| 3-Succinoylpyridine | 178.0 | 148.0 | 4.28 | -80 | -30 |
| Hippuric acid | 178.0 | 160.0 | 4.28 | -80 | -15 |
| 2-Acetyl-4-methylpyridine | 134.1 | 132.0 | 4.29 | -80 | -45 |
| 2-Hydroxy-4-methoxyacetophenone 5-sulfate | 261.0 | 259.0 | 4.30 | -80 | -15 |
| 4-Phospho-L-aspartate | 212.0 | 178.0 | 4.30 | -80 | -30 |
| L-Glutamine | 145.1 | 139.4 | 4.32 | -80 | -15 |
| Xanthosine 5'-phosphate | 363.0 | 285.1 | 4.33 | -80 | -30 |
| 5-Hydroxy-6-methoxycoumarin 7-glucoside | 369.1 | 194.0 | 4.34 | -80 | -15 |
| 2,3-Dihydroxybenzoic Acid | 153.0 | 135.0 | 4.37 | -80 | -15 |
| 3-(Benzoyloxy)-2-hydroxypropyl ?-D-glucopyranosiduronic acid | 371.1 | 369.1 | 4.40 | -80 | -15 |
| Portulacaxanthin II | 373.1 | 195.0 | 4.41 | -80 | -45 |
| 8-Oxo-dGDP | 442.0 | 264.1 | 4.46 | -80 | -15 |
| Dopaquinone | 194.0 | 160.8 | 4.46 | -80 | -30 |
| LPA 16:4 | 401.2 | 399.2 | 4.46 | -80 | -15 |
| 2-Isopropylmaleate | 157.0 | 141.1 | 4.49 | -80 | -15 |
| Indoxyl sulfate | 212.0 | 178.0 | 4.50 | -80 | -30 |
| 3,4-Dihydroxybenzoic acid | 153.0 | 138.0 | 4.53 | -80 | -45 |
| N-Feruloylglycine | 250.1 | 232.1 | 4.54 | -80 | -15 |
| Riboflavin-5-phosphate | 455.1 | 411.1 | 4.55 | -80 | -15 |
| Glutamine | 302.1 | 284.1 | 4.57 | -80 | -15 |
| Methyl 7-epi-12-hydroxyjasmonate glucoside | 401.2 | 399.2 | 4.60 | -80 | -15 |
| Daidzein | 253.0 | 223.1 | 4.65 | -80 | -30 |
| Phenylacetyl-L-glutamine | 263.1 | 245.1 | 4.66 | -80 | -15 |
| Tyrosyl-Tyrosine | 343.1 | 113.0 | 4.66 | -80 | -15 |
| Acetanilide | 134.1 | 75.0 | 4.67 | -80 | -15 |
| Guaiacol | 123.0 | 121.0 | 4.67 | -80 | -45 |
| p-Acetaminobenzoic acid | 178.0 | 136.1 | 4.67 | -80 | -15 |
| D-Glucose 6-phosphate | 259.0 | 257.0 | 4.68 | -80 | -15 |
| N-acetyl-2-carboxy Benzenesulfonamide | 242.0 | 225.1 | 4.69 | -80 | -15 |
| N-Acetyl-L-leucine | 172.1 | 131.1 | 4.71 | -80 | -15 |
| p-Hydroxyphenylacetylglycine | 208.1 | 206.0 | 4.73 | -80 | -15 |
| (-)-Epigallocatechin 3-(3-methyl-gallate) | 471.1 | 427.0 | 4.74 | -80 | -15 |
| 1,4-Cyclohexanedicarboxylic acid | 171.1 | 169.0 | 4.74 | -80 | -15 |
| Isoleucyl-Hydroxyproline | 243.1 | 226.1 | 4.76 | -80 | -30 |
| 2,2-Dimethylglutaric acid | 159.1 | 141.1 | 4.81 | -80 | -15 |
| 2-Methyladipic acid | 159.1 | 116.9 | 4.81 | -80 | -45 |
| 3,4-Methylenesebacic acid | 225.1 | 178.8 | 4.81 | -80 | -45 |
| N-lactoyl-Isoeucine | 201.1 | 184.1 | 4.82 | -80 | -15 |
| N-Phenylacetylaspartic acid | 250.1 | 207.1 | 4.83 | -80 | -15 |
| N-Phenylacetylglutamic acid | 264.1 | 184.0 | 4.83 | -80 | -45 |
| 3-Carbamoyl-2-phenylpropionic acid | 208.1 | 206.0 | 4.85 | -80 | -15 |
| p-Cresol glucuronide | 283.1 | 237.9 | 4.85 | -80 | -45 |
| 4-Cresol | 107.0 | 92.0 | 4.88 | -80 | -45 |
| p-Tolyl Sulfate | 187.0 | 109.0 | 4.88 | -80 | -45 |
| 3-Oxovalproic acid | 157.1 | 116.1 | 4.89 | -80 | -45 |
| 4-(2-Amino-1-Hydroxyethyl)-2-Methoxyphenol | 182.1 | 168.0 | 4.93 | -80 | -15 |
| Indoleacetyl glutamine | 302.1 | 223.1 | 4.94 | -80 | -45 |
| Dopamine quinone | 150.1 | 138.0 | 4.98 | -80 | -15 |
| 4-Formyl-3-(formylmethyl)-4-hexenoic acid | 183.1 | 168.0 | 4.99 | -80 | -45 |
| Adenine | 134.1 | 109.9 | 5.01 | -80 | -45 |
| Hydroxy-lacosamide | 281.1 | 265.1 | 5.03 | -80 | -15 |
| Myricitrin | 463.1 | 429.1 | 5.04 | -80 | -15 |
| Vitexin | 431.1 | 429.1 | 5.04 | -80 | -15 |
| Pseudoecgonine | 184.1 | 182.1 | 5.08 | -80 | -15 |
| L-(-)-3-Phenyllactic acid | 165.1 | 149.0 | 5.09 | -80 | -45 |
| 2,4-Dihydroxypteridine | 164.0 | 150.0 | 5.16 | -80 | -45 |
| Hexanoyl glycine | 172.1 | 130.1 | 5.19 | -80 | -30 |
| 5-Methyl-dl-tryptophan | 217.1 | 215.1 | 5.20 | -80 | -15 |
| 2-Oxohexanoic acid | 129.1 | 126.9 | 5.22 | -80 | -45 |
| 2-Descarboxy-cyclo-dopa | 150.1 | 136.0 | 5.23 | -80 | -30 |
| 4-Hydroxy-5-(dihydroxyphenyl)-valeric acid-O-sulphate | 305.0 | 285.1 | 5.26 | -80 | -15 |
| Indolelactate | 204.1 | 187.1 | 5.26 | -80 | -15 |
| Kaempferol 3-glucuronide | 461.1 | 286.0 | 5.28 | -80 | -45 |
| Kaempferol 3-rhamnoside 7-galacturonide | 607.1 | 445.1 | 5.29 | -80 | -15 |
| N-Gluconyl ethanolamine phosphate | 318.1 | 274.1 | 5.31 | -80 | -15 |
| Cytidine | 485.2 | 310.1 | 5.35 | -80 | -15 |
| Thiamylal | 253.1 | 233.1 | 5.35 | -80 | -15 |
| 4-(β-Acetylaminoethyl)imidazole | 153.1 | 109.0 | 5.36 | -80 | -15 |
| 2-n-Propyl-4-pentenoic acid | 141.1 | 113.9 | 5.42 | -80 | -15 |
| Rutin | 609.1 | 272.1 | 5.45 | -80 | -45 |
| Naringenin 4'-O-glucuronide | 447.1 | 273.2 | 5.47 | -80 | -30 |
| Naringenin-7-O-β-D-Glucuronide | 447.1 | 445.1 | 5.47 | -80 | -15 |
| Quercetin 3-β-D-glucoside | 463.1 | 461.1 | 5.48 | -80 | -15 |
| Dibutyl malate | 245.1 | 243.1 | 5.49 | -80 | -15 |
| 3-oxo-C10-homoserine lactone | 268.2 | 225.1 | 5.53 | -80 | -15 |
| 5-Methoxyindole-3-acetic acid | 204.1 | 161.8 | 5.55 | -80 | -15 |
| 2-Phenylethanol glucuronide | 297.1 | 219.0 | 5.56 | -80 | -45 |
| 2-Hydroxyquinoline | 144.0 | 116.9 | 5.57 | -80 | -15 |
| N-Acetyl-L-tyrosine | 222.1 | 179.1 | 5.60 | -80 | -45 |
| Hexestrol | 269.1 | 252.1 | 5.65 | -80 | -15 |
| Isoferulic acid 3-O-glucuronide | 369.1 | 352.1 | 5.65 | -80 | -15 |
| Sakuranetin | 285.1 | 257.9 | 5.67 | -80 | -15 |
| 5-Hydroxytryptophan | 439.2 | 437.1 | 5.71 | -80 | -15 |
| N-HISTIDYL-2-AMINONAPHTHALENE (βNA) | 279.1 | 238.1 | 5.75 | -80 | -45 |
| Cortolone-3-glucuronide | 541.3 | 539.2 | 5.76 | -80 | -15 |
| D-Galactopyranosyl-(1->3)-D-galactopyranosyl-(1->3)-L-arabinose | 473.1 | 455.1 | 5.78 | -80 | -15 |
| Salicylic acid | 137.0 | 111.0 | 5.83 | -80 | -45 |
| Tricin 7-glucuronoside | 505.1 | 314.0 | 5.85 | -80 | -45 |
| Estrone glucuronide | 445.2 | 443.2 | 5.87 | -80 | -45 |
| N-(3-Indolylacetyl)-L-isoleucine | 287.1 | 285.1 | 5.92 | -80 | -15 |
| Asparaginyl-Arginine | 287.1 | 285.1 | 5.93 | -80 | -30 |
| ESTRA-4,9-DIENE-3,17-DIONE | 269.1 | 253.1 | 5.97 | -80 | -15 |
| Naringenin 5-O-glucuronide | 447.1 | 272.1 | 5.99 | -80 | -45 |
| Dipropyl hexanedioate | 229.1 | 185.1 | 6.07 | -80 | -30 |
| D-Ribose5-phophate | 229.0 | 211.1 | 6.07 | -80 | -15 |
| LPA 22:5 | 483.3 | 481.2 | 6.11 | -80 | -15 |
| α-Zearalanol | 357.2 | 323.0 | 6.11 | -80 | -15 |
| 16α-hydroxydehydroepiandrosterone 3-sulfate | 383.2 | 301.1 | 6.12 | -80 | -45 |
| Sebacate | 201.1 | 199.1 | 6.15 | -80 | -30 |
| Arginyl-Asparagine | 287.1 | 285.1 | 6.18 | -80 | -15 |
| Glycitein 7-O-glucuronide | 459.1 | 425.2 | 6.19 | -80 | -15 |
| Tetrahydroaldosterone-3-glucuronide | 539.2 | 521.2 | 6.22 | -80 | -30 |
| D-Xylulose 5-phosphate | 229.0 | 187.1 | 6.26 | -80 | -15 |
| 3,3',5-Trihydroxy-4'-methoxy-6,7-methylenedioxyflavone 3-glucuronide | 519.1 | 481.2 | 6.28 | -80 | -15 |
| Lactobionic acid | 357.1 | 320.9 | 6.28 | -80 | -15 |
| Neuraminic acid | 266.1 | 221.8 | 6.32 | -80 | -45 |
| 7-Methylguanosine 5'-phosphate | 377.1 | 343.1 | 6.33 | -80 | -15 |
| Phe-Phe | 311.1 | 273.2 | 6.35 | -80 | -15 |
| Valproic acid glucuronide | 319.1 | 305.1 | 6.35 | -80 | -45 |
| Glycocholic acid | 464.3 | 462.3 | 6.37 | -80 | -15 |
| Sodium glycocholate | 464.3 | 420.3 | 6.37 | -80 | -45 |
| Cortisol 21-sulfate | 441.2 | 439.2 | 6.38 | -80 | -15 |
| Hydrocortisone 17-valerate | 481.2 | 479.2 | 6.38 | -80 | -15 |
| Dihydronaringenin-O-sulphate | 353.0 | 318.9 | 6.42 | -80 | -15 |
| (2S,3S,4S,5R,6S)-3,4,5-trihydroxy-6-[5-hydroxy-6-methoxy-2-(4-methoxyphenyl)-4-oxochromen-7-yl]oxyoxane-2-carboxylic acid | 489.1 | 314.1 | 6.43 | -80 | -30 |
| Phenethyl rutinoside | 429.2 | 383.1 | 6.52 | -80 | -15 |
| 11-Oxo-androsterone glucuronide | 479.2 | 175.0 | 6.53 | -80 | -15 |
| Ursocholate | 407.3 | 363.3 | 6.53 | -80 | -45 |
| Picrocrocin | 329.2 | 175.0 | 6.54 | -80 | -15 |
| PA 24:6 | 523.3 | 486.1 | 6.55 | -80 | -15 |
| Geranyl monophosphate | 467.2 | 465.2 | 6.58 | -80 | -15 |
| 17α-Ethinylestradiol | 295.2 | 146.1 | 6.59 | -80 | -45 |
| 11-β-Hydroxyandrosterone-3-glucuronide | 481.2 | 479.2 | 6.62 | -80 | -15 |
| 2,3-Butanediol apiosylglucoside | 383.2 | 381.1 | 6.65 | -80 | -15 |
| 4-hydroxy ketorolac | 270.1 | 253.1 | 6.66 | -80 | -30 |
| ACETAMINOSALOL | 270.1 | 228.1 | 6.66 | -80 | -45 |
| Octanoylglucuronide | 319.1 | 239.2 | 6.66 | -80 | -45 |
| Testosterone glucuronide | 463.2 | 445.2 | 6.67 | -80 | -30 |
| Tetranor-PGFM | 329.2 | 175.0 | 6.69 | -80 | -15 |
| 6-Thioinosine-5'-monophosphate | 363.0 | 329.2 | 6.70 | -80 | -15 |
| Feruloylagmatine | 305.2 | 113.0 | 6.70 | -80 | -45 |
| Methyl 3-(2,3-dihydroxy-3-methylbutyl)-4-hydroxybenzoate | 253.1 | 236.1 | 6.70 | -80 | -30 |
| 5-Formiminotetrahydrofolate | 471.2 | 423.2 | 6.73 | -80 | -15 |
| 7-Epi-12-hydroxyjasmonic acid glucoside | 387.2 | 299.0 | 6.77 | -80 | -45 |
| α-Terpinyl anthranilate | 272.2 | 227.2 | 6.78 | -80 | -30 |
| 1-(4Z,7Z,10Z,13Z,16Z,19Z-docosahexaenoyl)-glycero-3-phosphate | 481.2 | 479.2 | 6.79 | -80 | -15 |
| 5-Heptyltetrahydro-2-oxo-3-furancarboxylic acid | 227.1 | 209.1 | 6.80 | -80 | -45 |
| Cholic acid glucuronide | 583.3 | 227.1 | 6.81 | -80 | -15 |
| 3'-Hydroxy-T2 Toxin | 481.2 | 397.1 | 6.83 | -80 | -15 |
| Dehydroisoandrosterone 3-glucuronide | 463.2 | 397.1 | 6.83 | -80 | -15 |
| 20-Hydroxyecdysone | 515.3 | 513.3 | 6.84 | -80 | -15 |
| Diferuloylputrescine | 439.2 | 395.2 | 6.91 | -80 | -15 |
| N-Undecanoylglycine | 242.2 | 226.2 | 6.97 | -80 | -30 |
| Genistein | 269.0 | 241.0 | 6.98 | -80 | -45 |
| Hyocholate | 407.3 | 333.2 | 6.98 | -80 | -30 |
| Allocholic acid | 407.3 | 389.3 | 6.99 | -80 | -45 |
| Pantethine | 553.2 | 378.2 | 7.00 | -80 | -15 |
| Glaucarubin | 495.2 | 493.1 | 7.01 | -80 | -15 |
| 3α-Hydroxyoreadone | 251.1 | 235.1 | 7.02 | -80 | -30 |
| 3,17-Androstanediol glucuronide | 467.3 | 465.2 | 7.06 | -80 | -15 |
| Cortisone acetate | 402.2 | 239.2 | 7.06 | -80 | -45 |
| Apigenin | 269.0 | 241.0 | 7.12 | -80 | -45 |
| Glycochenodeoxycholic acid 3-glucuronide | 624.3 | 588.1 | 7.12 | -80 | -15 |
| Dodecanedioic aicd | 229.1 | 211.1 | 7.22 | -80 | -45 |
| Glycocholic acid hydrate475-31-0 | 464.3 | 462.2 | 7.26 | -80 | -15 |
| Glycochenodeoxycholic acid | 448.3 | 446.3 | 7.30 | -80 | -30 |
| Androsterone glucuronide | 465.2 | 447.2 | 7.34 | -80 | -30 |
| 17-phenyl trinor PGF2αisopropyl ester | 465.2 | 463.2 | 7.35 | -80 | -15 |
| Guggulsterone | 311.2 | 267.2 | 7.38 | -80 | -15 |
| α-Ionol O-[arabinosyl-(1->6)-glucoside] | 487.3 | 485.2 | 7.40 | -80 | -15 |
| (2S)-OMPT | 465.2 | 448.2 | 7.44 | -80 | -30 |
| Glycochenodeoxycholate-3-sulfate | 528.3 | 467.3 | 7.44 | -80 | -30 |
| (R)-Butaprost (free acid) | 393.3 | 349.3 | 7.46 | -80 | -45 |
| Arginyl-γ-glutamate | 301.2 | 257.2 | 7.47 | -80 | -15 |
| LPG 22:4 | 559.3 | 213.0 | 7.48 | -80 | -15 |
| 7-Ketodeoxycholic acid | 405.3 | 113.0 | 7.52 | -80 | -30 |
| DL-Glyceraldehyde 2-Phosphate | 169.0 | 134.9 | 7.54 | -80 | -30 |
| Leukotriene D4 methyl ester | 509.3 | 175.0 | 7.56 | -80 | -15 |
| LPA 17:2 | 419.2 | 417.2 | 7.57 | -80 | -15 |
| 9-Hydroxy-10-O-D-glucuronoside-12Z-octadecenoate | 489.3 | 471.3 | 7.62 | -80 | -30 |
| PA 21:2 | 489.3 | 487.3 | 7.62 | -80 | -15 |
| Hydrocortisone caproate | 183.1 | 167.1 | 7.63 | -80 | -15 |
| PS 16:0 | 510.3 | 448.3 | 7.63 | -80 | -15 |
| LPI 16:0 | 571.3 | 509.3 | 7.72 | -80 | -15 |
| 3-(4-Deoxy-β-D-gluc-4-enuronosyl)-N-acetyl-D-glucosamine | 378.1 | 299.1 | 7.76 | -80 | -15 |
| all-trans-Retinoyl-β-glucuronide | 475.2 | 299.2 | 7.76 | -80 | -45 |
| Taurocholic acid | 514.3 | 446.3 | 7.77 | -80 | -15 |
| 10-Hydroxy-octadec-12Z-enoate-9-β-D-glucuronide | 489.3 | 471.3 | 7.80 | -80 | -30 |
| 12-Hydroxy-13-O-D-glucuronoside-octadec-9Z-enoate | 489.3 | 175.0 | 7.80 | -80 | -15 |
| 6-Methylaminopurine arabinoside | 561.2 | 495.3 | 7.80 | -80 | -15 |
| Chenodeoxycholic acid 3-sulfate | 471.2 | 469.2 | 7.94 | -80 | -15 |
| 15(R)-17-phenyl trinor PGF2αisopropyl ester | 465.2 | 369.2 | 8.00 | -80 | -15 |
| Cholic acid | 407.3 | 389.3 | 8.08 | -80 | -45 |
| PA 27:5 | 567.3 | 565.3 | 8.08 | -80 | -15 |
| Glycoursodeoxycholic acid | 448.3 | 446.3 | 8.14 | -80 | -15 |
| Glycodeoxycholic acid | 448.3 | 446.3 | 8.15 | -80 | -30 |
| D-Urobilinogen | 589.3 | 255.1 | 8.22 | -80 | -45 |
| Urobilin | 589.3 | 466.2 | 8.22 | -80 | -15 |
| 12α-Hydroxy-3-oxochola-4,6-dienoate | 385.2 | 383.2 | 8.25 | -80 | -30 |
| 3-α,20-α-dihydroxy-5-β-pregnane 3-glucuronide | 495.3 | 493.3 | 8.25 | -80 | -30 |
| D-Norvaline | 293.2 | 237.1 | 8.31 | -80 | -45 |
| 3-carboxy-4-methyl-5-pentyl-2-furanpropanoic acid | 267.1 | 225.1 | 8.32 | -80 | -30 |
| 15-keto Latanoprost | 465.2 | 283.3 | 8.36 | -80 | -30 |
| Sulfoglycolithocholate | 512.3 | 510.2 | 8.41 | -80 | -15 |
| Chenodeoxycholic acid sulfate | 471.2 | 297.2 | 8.42 | -80 | -30 |
| PA 30:8 | 603.3 | 427.3 | 8.44 | -80 | -15 |
| Deoxycholic acid 3-glucuronide | 567.3 | 85.0 | 8.50 | -80 | -45 |
| 16,16-dimethyl-6-keto Prostaglandin E1 | 395.2 | 277.2 | 8.54 | -80 | -45 |
| Epiandrosterone sulfate | 369.2 | 367.2 | 8.61 | -80 | -30 |
| L-Norvaline | 293.2 | 236.1 | 8.67 | -80 | -45 |
| Benzene-1,2,4-triol | 125.0 | 97.0 | 8.82 | -80 | -45 |
| N-Feruloylaspartic acid | 308.1 | 194.1 | 8.83 | -80 | -45 |
| 6-Gingerol | 293.2 | 253.1 | 8.86 | -80 | -15 |
| 1,2,3-Trihydroxybenzene | 125.0 | 82.9 | 9.02 | -80 | -15 |
| Metyrosine | 194.1 | 160.8 | 9.19 | -80 | -45 |
| 5β-Androstan-3α-ol-17-one sulfate | 369.2 | 367.2 | 9.26 | -80 | -15 |
| all-trans-Pentaprenyl diphosphate | 517.2 | 450.3 | 9.42 | -80 | -15 |
| Dehydroisoandrosterone sulfate | 367.2 | 97.0 | 9.45 | -80 | -30 |
| Dehydroepiandrosterone sulfate | 367.2 | 97.0 | 9.60 | -80 | -45 |
| MGDG 18:0 | 579.3 | 391.2 | 9.68 | -80 | -15 |
| Curcumenone | 233.2 | 199.9 | 10.07 | -80 | -30 |
| LPA 18:0 | 437.3 | 435.2 | 10.20 | -80 | -15 |
| PG 22:1 | 579.3 | 409.2 | 10.20 | -80 | -15 |
| PC 36:6 | 822.5 | 764.5 | 10.29 | -80 | -15 |
| PC 33:3 | 800.5 | 480.3 | 10.46 | -80 | -30 |
| 3-β-hydroxyandrost-5-en-17-one sulfate | 367.2 | 99.0 | 10.55 | -80 | -45 |
| SM d30:1 | 691.5 | 627.5 | 11.06 | -80 | -45 |
| LPG 21:2 | 549.3 | 409.2 | 11.11 | -80 | -15 |
| N-Desmethyleletriptan | 367.2 | 97.0 | 11.36 | -80 | -30 |
| LPC 19:0 | 582.4 | 580.3 | 11.45 | -80 | -15 |
| 2-Ethylthiazole | 112.0 | 82.0 | 11.79 | -80 | -30 |
| α-Hydroxyhippuric acid | 194.0 | 176.9 | 11.80 | -80 | -15 |
| PE 36:5 | 736.5 | 734.4 | 11.82 | -80 | -15 |
| PC(15:0/18:2(9Z,12Z)) | 742.5 | 284.3 | 11.86 | -80 | -30 |
| PC(15:0/18:3(6Z,9Z,12Z)) | 740.5 | 284.3 | 11.93 | -80 | -45 |
| PC 36:5 | 824.5 | 766.5 | 12.00 | -80 | -15 |
| PC 34:3 | 800.5 | 480.3 | 12.58 | -80 | -30 |
| 2-Carboxy-2,3-dihydro-5,6-dihydroxyindole | 194.0 | 160.8 | 12.95 | -80 | -30 |
| 5a-Dihydrotestosterone sulfate | 369.2 | 367.2 | 12.99 | -80 | -15 |
| PC 34:2 | 802.6 | 281.2 | 13.17 | -80 | -45 |
| 16-phenoxy tetranor Prostaglandin A2 | 369.2 | 367.2 | 13.57 | -80 | -15 |
| Methyl (9Z)-10'-oxo-6,10'-diapo-6-carotenoate | 311.2 | 222.8 | 13.61 | -80 | -30 |
| DGDG 38:2 | 1017.7 | 972.7 | 13.63 | -80 | -15 |
| Acitretin | 325.2 | 183.0 | 14.03 | -80 | -30 |
| Cer-AP t40:1 | 652.6 | 355.3 | 14.03 | -80 | -30 |
| HexCer-AP t43:1 | 902.7 | 858.7 | 14.06 | -80 | -15 |
| Cer-AP t42:1 | 726.6 | 337.3 | 14.10 | -80 | -45 |
| HexCer-NP t42:2 | 870.7 | 826.7 | 14.24 | -80 | -15 |
| HexCer-AS d42:2 | 870.7 | 826.7 | 14.64 | -80 | -15 |
| 7-Formyldehydrothalicsimidine | 410.2 | 326.2 | 14.66 | -80 | -15 |
| 13-Cis-Acitretin | 325.2 | 183.0 | 14.68 | -80 | -30 |
| Cholesterol sulfate | 465.3 | 116.9 | 14.68 | -80 | -45 |
| Norethindrone acetate | 339.2 | 183.0 | 14.69 | -80 | -45 |
| PA(18:0/18:2(9Z,12Z)) | 699.5 | 697.5 | 14.69 | -80 | -15 |
| 1,2-Di-(9Z,12Z-octadecadienoyl)-sn-glycero-3-phosphate | 695.5 | 647.5 | 14.71 | -80 | -30 |
| PA 34:2 | 671.5 | 409.2 | 14.71 | -80 | -30 |
| PA 34:3 | 669.4 | 415.2 | 14.71 | -80 | -15 |
| PA 36:5 | 693.4 | 433.2 | 14.71 | -80 | -15 |
| PC 36:4 | 826.6 | 768.5 | 14.71 | -80 | -15 |
| PG 34:3 | 743.5 | 279.2 | 14.71 | -80 | -15 |
| LPG 22:2 | 563.3 | 409.2 | 14.72 | -80 | -15 |
| L-Urobilin | 593.3 | 437.3 | 14.72 | -80 | -15 |
| PE 34:2 | 714.5 | 452.3 | 14.72 | -80 | -30 |
| PE 36:4 | 738.5 | 736.5 | 14.72 | -80 | -15 |
| PG 24:1 | 607.4 | 605.3 | 14.72 | -80 | -15 |
| PG 31:3 | 701.4 | 190.9 | 14.72 | -80 | -15 |
| PG(16:0/18:2(9Z,12Z)) | 745.5 | 283.3 | 14.72 | -80 | -45 |
| PG(16:0/18:3(9Z,12Z,15Z)) | 743.5 | 283.3 | 14.72 | -80 | -45 |
| SQDG 30:2 | 761.5 | 759.4 | 14.72 | -80 | -15 |
| 1-hexadecanoyl-2-(9Z,12Z-octadecadienoyl)-sn-glycero-3-phospho-D-myo-inositol | 833.5 | 283.3 | 14.73 | -80 | -45 |
| GlcADG 31:4 | 721.5 | 719.4 | 14.73 | -80 | -15 |
| GlcADG 31:5 | 719.4 | 717.4 | 14.73 | -80 | -15 |
| LPA(0:0/18:0) | 437.3 | 153.0 | 14.73 | -80 | -15 |
| PI 34:3 | 831.5 | 279.2 | 14.73 | -80 | -45 |
| LPS 20:0 | 552.3 | 466.3 | 14.74 | -80 | -15 |
| 1-Palmitoylglycerol 3-phosphate | 409.2 | 153.0 | 14.75 | -80 | -30 |
| SM d30:1 | 691.5 | 627.5 | 11.06 | -80 | -45 |
| LPC 19:0 | 582.4 | 580.3 | 11.45 | -80 | -15 |

DP: declustering potential; CE: collision energy.
