# Supplementary material for: Biomarkers of Metabolomics in Inflammatory Bowel Disease and Damp-Heat Syndrome: A Preliminary Study
Source: Evid Based Complement Alternat Med. 2022 Jul 1;2022:3319646. doi: 10.1155/2022/3319646 (PMC9270137; doi:10.1155/2022/3319646)
Supplement: Supplementary Materials — Supplementary Material 1: damp-heat syndrome and spleen deficiency syndrome diagnosis standards and untargeted metabolomics based UHPLC-HRMS and multilevel metabolite characterization system. Supplementary Material 2: plasma ion-pairs information in positive mode and negative mode. Supplementary Material 3: pseudotargeted metabolomics method of urine samples. Supplementary Material 4: urine ion-pairs information in positive mode and negative mode. Supplementary Material 5: plasma and urine differential metabolites in IBD versus Con; plasma differential metabolites in IBD-DH versus IBD-SD. [file 3319646.f1.zip › 3319646.f1/Supplementary material 5 (1).doc]

Table S1 Plasma differential metabolites in IBD vs Con

| Compounds | Class | FC | log2(FC) | p | FDR | VIP |
| --- | --- | --- | --- | --- | --- | --- |
| Arginine | Carboxylic acids and derivatives | 0.411 | -1.283 | <0.001 | ＜0.001 | 3.288 |
| 3-Indoleacetic acid | Indoles and derivatives | 56.995 | 5.833 | <0.001 | 0.001 | 1.898 |
| Acar 9:1 | Fatty Acyls | 0.578 | -0.792 | 0.002 | 0.036 | 1.829 |
| ACar 20:4 | Fatty Acyls | 2.620 | 1.390 | <0.001 | ＜0.001 | 2.244 |
| LPE 22:4-2 | Glycerophospholipids | 1.631 | 0.706 | 0.002 | 0.029 | 1.741 |
| LPC P-18:0 | Glycerophospholipids | 1.595 | 0.673 | 0.001 | 0.022 | 1.836 |
| 5-Acetoxypalisadin-B | Carboxylic acids and derivatives | 3.370 | 1.753 | <0.001 | 0.007 | 2.252 |
| Stigmastentriol | Steroids and steroid derivatives | 3.255 | 1.703 | <0.001 | 0.006 | 2.281 |
| ACar 16:0 | Fatty Acyls | 1.692 | 0.759 | 0.001 | 0.022 | 1.647 |
| ACar 18:2 | Fatty Acyls | 1.866 | 0.900 | <0.001 | 0.004 | 2.027 |
| Palmitoleic acid | Fatty Acyls | 1.912 | 0.935 | 0.002 | 0.030 | 1.496 |
| ACar 18:1 | Fatty Acyls | 2.375 | 1.248 | <0.001 | ＜0.001 | 2.340 |
| Monocrotaline | Pyrrolizines | 2.334 | 1.223 | <0.001 | 0.001 | 2.056 |
| ACar 20:3 | Fatty Acyls | 2.410 | 1.269 | <0.001 | ＜0.001 | 2.360 |
| PE 36:5 | Glycerophospholipids | 2.162 | 1.112 | <0.001 | 0.007 | 1.856 |
| PC 33:3 | Glycerophospholipids | 0.629 | -0.670 | 0.002 | 0.029 | 2.207 |
| PE 34:3 | Glycerophospholipids | 2.647 | 1.405 | <0.001 | 0.001 | 2.053 |
| DAG 36:3 | Glycerolipids | 1.640 | 0.714 | 0.001 | 0.018 | 1.796 |
| L-Glutamic acid | Carboxylic acids and derivatives | 1.827 | 0.870 | <0.001 | ＜0.001 | 2.364 |
| 2-Oxoglutarate | Keto acids and derivatives | 1.562 | 0.643 | 0.001 | 0.008 | 1.263 |
| 3-Oxopentanedioic Acid | Keto acids and derivatives | 1.541 | 0.624 | 0.001 | 0.011 | 1.253 |
| Glyceraldehyde-3-phosphate | Organooxygen compounds | 1.998 | 0.998 | 0.001 | 0.011 | 1.445 |
| malate | Hydroxy acids and derivatives | 1.781 | 0.833 | <0.001 | ＜0.001 | 1.939 |
| Pyruvate | Keto acids and derivatives | 1.820 | 0.864 | <0.001 | 0.001 | 2.172 |
| Lactic acid | Hydroxy acids and derivatives | 1.984 | 0.988 | <0.001 | ＜0.001 | 2.662 |
| D-Pantothenic acid hemicalcium salt | Carboxylic acids and derivatives | 2.312 | 1.209 | <0.001 | 0.001 | 1.653 |
| 2-Methyladipic acid | Fatty Acyls | 2.650 | 1.406 | 0.003 | 0.019 | 1.158 |
| 4-Pyridoxic acid | Pyridines and derivatives | 2.166 | 1.115 | <0.001 | ＜0.001 | 1.839 |
| 3-Hydroxymugineic acid | Carboxylic acids and derivatives | 405.340 | 8.663 | <0.001 | <0.001 | 1.943 |
| 4-Acetamidophenol | Phenols | 7.733 | 2.951 | <0.001 | 0.001 | 1.481 |
| beta-Hydroxyhippuric acid | Benzene and substituted derivatives | 277.500 | 8.116 | <0.001 | <0.001 | 2.007 |
| Hippuric acid | Benzene and substituted derivatives | 0.281 | -1.830 | <0.001 | 0.002 | 2.166 |
| 5-O-Caffeoylquinic acid methyl ester | Organooxygen compounds | 0.391 | -1.355 | 0.004 | 0.027 | 1.492 |
| 3-Hydroxyphenylacetate | Phenols | 0.348 | -1.521 | 0.009 | 0.050 | 1.587 |
| 7z,10z,13z-Hexadecatrienoic Acid | Fatty Acyls | 0.552 | -0.858 | 0.009 | 0.050 | 1.425 |
| FFA 24:4 | Fatty Acyls | 0.321 | -1.638 | <0.001 | 0.002 | 2.128 |
| Hexadecanoyl-hydroxy-sn-glycerophosphocholine(PUT) |  | 2.815 | 1.493 | <0.001 | 0.003 | 1.664 |
| Taurodeoxycholic acid | Steroids and steroid derivatives | 0.474 | -1.077 | 0.001 | 0.009 | 1.974 |
| ANDS/DHTS | Steroids and steroid derivatives | 0.639 | -0.645 | 0.007 | 0.043 | 1.221 |
| 15,16-DiHODE | Fatty Acyls | 0.480 | -1.058 | 0.006 | 0.038 | 1.484 |
| Biliverdin | Tetrapyrroles and derivatives | 0.303 | -1.721 | 0.002 | 0.015 | 2.002 |
| N-palmitoyl-phosphoethanolamine | Organic phosphonic acids and derivatives | 1.569 | 0.650 | <0.001 | 0.002 | 1.507 |
| FA 20:2 | Fatty Acyls | 0.381 | -1.393 | 0.009 | 0.050 | 1.642 |
| FA 22:3 | Fatty Acyls | 0.616 | -0.700 | 0.006 | 0.037 | 1.437 |
| 7beta,12alpha-Dihydroxy-5alpha-cholan-24-oic acid | Steroids and steroid derivatives | 0.348 | -1.524 | 0.003 | 0.019 | 1.624 |
| 8(R)-Hydroxy-(5Z,9E,11Z,14Z)-eicosatetraenoic acid | Fatty Acyls | 2.741 | 1.455 | <0.001 | <0.001 | 1.796 |
| LPG 9:0 | Glycerophospholipids | 0.469 | -1.093 | <0.001 | 0.001 | 2.009 |
| Glycodeoxycholic acid | Steroids and steroid derivatives | 0.523 | -0.936 | 0.001 | 0.011 | 1.625 |
| Persenone A | Fatty Acyls | 0.359 | -1.480 | <0.001 | 0.001 | 2.200 |
| Dehydroepiandrosterone sulfate | Steroids and steroid derivatives | 0.288 | -1.796 | <0.001 | 0.002 | 2.089 |
| FAHFA 28:0 | Fatty Acyls | 0.364 | -1.457 | 0.003 | 0.023 | 1.494 |
| 9'-Carboxy-alpha-chromanol | Benzopyrans | 0.283 | -1.823 | <0.001 | <0.001 | 2.448 |
| Ganoderiol C | Prenol lipids | 0.299 | -1.740 | <0.001 | <0.001 | 2.539 |
| 5b-Cholestane-3a,7a,12a,24,25-pentol | Steroids and steroid derivatives | 0.248 | -2.014 | <0.001 | 0.006 | 2.116 |
| 12,13-DHOME | Fatty Acyls | 5.613 | 2.489 | 0.002 | 0.019 | 1.087 |
| (R)-2-hydroxystearic acid | Fatty Acyls | 2.014 | 1.010 | 0.004 | 0.028 | 1.072 |

Table S2 Urine differential metabolites in IBD vs Con

| Compounds | Class | FC | log2(FC) | p | FDR | VIP |
| --- | --- | --- | --- | --- | --- | --- |
| (-)-3-(3,4-Dihydroxyphenyl)-2-methylalanine | Phenylpropanoic acids | 133.580 | 7.062 | <0.001 | 0.001 | 1.740 |
| 3-(3,4-Dihydroxyphenyl)-2-methyl-L-alanine | Phenylpropanoic acids | 7.634 | 2.932 | <0.001 | 0.004 | 1.518 |
| 5-Formiminotetrahydrofolate | Pteridines and derivatives | 23.059 | 4.527 | 0.002 | 0.039 | 1.493 |
| 5-Hydroxy-7-methoxy-6-methylflavone | Flavonoids | 54.298 | 5.763 | <0.001 | 0.008 | 1.369 |
| Citicoline | Pyrimidine nucleotides | 19.673 | 4.298 | 0.003 | 0.049 | 1.300 |
| Cytidine | Pyrimidine nucleosides | 11.961 | 3.580 | <0.001 | 0.008 | 1.399 |
| Dihydrocaffeic acid 3-O-glucuronide | Organooxygen compounds | 44.467 | 5.475 | 0.001 | 0.030 | 1.359 |
| Formononetin | Isoflavonoids | 5.343 | 2.418 | <0.001 | 0.014 | 1.332 |
| Glycochenodeoxycholate-3-sulfate | Steroids and steroid derivatives | 14.204 | 3.828 | 0.001 | 0.026 | 1.248 |
| Glycylglycine | Carboxylic acids and derivatives | 30.423 | 4.927 | <0.001 | 0.001 | 1.785 |
| Guanosine 5'-diphosphate | Purine nucleotides | 46.046 | 5.525 | <0.001 | <0.001 | 1.910 |
| Lactobionic acid | Fatty Acyls | 24.950 | 4.641 | <0.001 | 0.012 | 1.465 |
| LPG 22:2 | Glycerophospholipids | 3.023 | 1.596 | 0.001 | 0.016 | 1.313 |
| N4-Acetylcytidine | Pyrimidine nucleosides | 7.812 | 2.966 | <0.001 | 0.004 | 1.529 |
| N-Gluconyl ethanolamine phosphate | Organic phosphoric acids and derivatives | 42.352 | 5.404 | <0.001 | 0.001 | 1.655 |
| N-Phenylacetylaspartic acid | Carboxylic acids and derivatives | 5.226 | 2.386 | 0.001 | 0.026 | 1.399 |
| n-Propyl gallate | Benzene and substituted derivatives | 10.799 | 3.433 | <0.001 | 0.001 | 1.653 |
| Octanoylglucuronide | Organooxygen compounds | 9.544 | 3.255 | <0.001 | 0.008 | 1.509 |
| Pantetheine 4'-phosphate | Organic phosphoric acids and derivatives | 14.137 | 3.821 | 0.003 | 0.049 | 1.144 |
| α-Terpinyl anthranilate | Prenol lipids | 24.579 | 4.619 | <0.001 | 0.001 | 1.707 |

Table S3 Plasma and urine differential metabolites (AUC≥0.8 ) in IBD vs Con

| NO | Compounds | AUC | Sensitivity (%) | Specificity (%) | Trend |
| --- | --- | --- | --- | --- | --- |
| P1 | 3-Indoleacetic acid | 0.86 | 86.67 | 80.00 | ↑ |
| P2 | ACar 20:4 | 0.88 | 86.67 | 83.33 | ↑ |
| P3 | ACar 18:2 | 0.82 | 73.33 | 83.33 | ↑ |
| P4 | ACar 18:1 | 0.87 | 80.00 | 80.00 | ↑ |
| P5 | ACar 20:3 | 0.88 | 86.67 | 76.60 | ↑ |
| P6 | PE 36:5 | 0.81 | 80.00 | 80.00 | ↑ |
| P7 | PE 34:3 | 0.84 | 73.33 | 86.67 | ↑ |
| P8 | L-Glutamic acid | 0.95 | 93.33 | 86.67 | ↑ |
| P9 | 2-Oxoglutarate | 0.80 | 86.67 | 80.00 | ↑ |
| P10 | Glyceraldehyde-3-phosphate | 0.92 | 93.33 | 93.33 | ↑ |
| P11 | malate | 0.88 | 80.00 | 83.33 | ↑ |
| P12 | Pyruvate | 0.87 | 80.00 | 93.33 | ↑ |
| P13 | Lactic acid | 0.99 | 100.00 | 93.30 | ↑ |
| P14 | D-Pantothenic acid hemicalcium salt | 0.83 | 73.33 | 80.00 | ↑ |
| P15 | 3-Hydroxymugineic acid | 0.89 | 93.30 | 83.33 | ↑ |
| P16 | beta-Hydroxyhippuric acid | 0.81 | 93.30 | 76.60 | ↑ |
| P17 | Hippuric acid | 0.88 | 80.00 | 93.30 | ↓ |
| P18 | FFA 24:4 | 0.91 | 80.00 | 86.60 | ↓ |
| P19 | Hexadecanoyl-hydroxy-sn-glycerophosphocholine (PUT) | 0.83 | 86.67 | 73.30 | ↑ |
| P20 | Taurodeoxycholic acid | 0.86 | 73.30 | 86.60 | ↓ |
| P21 | 15,16-DiHODE | 0.82 | 86.67 | 70.00 | ↓ |
| P22 | N-palmitoyl-phosphoethanolamine | 0.80 | 80.00 | 80.00 | ↑ |
| P23 | 7beta,12alpha-Dihydroxy-5alpha-cholan-24-oic acid | 0.84 | 73.33 | 86.67 | ↓ |
| P24 | 8(R)-Hydroxy-(5Z,9E,11Z,14Z)-eicosatetraenoic acid | 0.89 | 93.33 | 86.67 | ↑ |
| P25 | LPG 9:0 | 0.88 | 93.33 | 73.30 | ↓ |
| P26 | Glycodeoxycholic acid | 0.83 | 93.33 | 66.70 | ↓ |
| P27 | Dehydroepiandrosterone sulfate | 0.9 | 93.33 | 83.33 | ↓ |
| P28 | 9'-Carboxy-alpha-chromanol | 0.95 | 86.67 | 90.00 | ↓ |
| P29 | 5b-Cholestane-3a,7a,12a,24,25-pentol | 0.93 | 80.00 | 93.33 | ↓ |
| U1 | (-)-3-(3,4-Dihydroxyphenyl)-2-methylalanine | 0.91 | 86.67 | 80 | ↑ |
| U2 | 3-(3,4-Dihydroxyphenyl)-2-methyl-L-alanine | 0.85 | 80 | 76.67 | ↑ |
| U3 | Dihydrocaffeic acid 3-O-glucuronide | 0.84 | 100 | 73.3 | ↑ |
| U4 | Glycylglycine | 0.86 | 86.67 | 80 | ↑ |
| U5 | Guanosine 5'-diphosphate | 0.88 | 80 | 90 | ↑ |
| U6 | Lactobionic acid | 0.83 | 100 | 73.3 | ↑ |
| U7 | N-Gluconyl ethanolamine phosphate | 0.84 | 93.3 | 83.3 | ↑ |
| U8 | N-Phenylacetylaspartic acid | 0.82 | 86.67 | 66.67 | ↑ |
| U9 | n-Propyl gallate | 0.84 | 100 | 73.3 | ↑ |
| U10 | Octanoylglucuronide | 0.82 | 86.67 | 76.67 | ↑ |
| U11 | α-Terpinyl anthranilate | 0.9 | 93.3 | 80 | ↑ |

NO: number; P: plasma; U: urine

Table S4 Plasma differential metabolites in IBD-DH vs IBD-SD

| Compounds | Class | FC | log2(FC) | VIP | P | FDR |
| --- | --- | --- | --- | --- | --- | --- |
| L-Tryptophan | Indoles and derivatives | 1.952 | 0.965 | 1.741 | 0.015 | 0.171 |
| cyclo-(Val-Leu) | Carboxylic acids and derivatives | 1.541 | 0.623 | 2.075 | 0.002 | 0.104 |
| ACar 8:1 | Fatty Acyls | 1.612 | 0.689 | 2.052 | 0.002 | 0.104 |
| LPC 20:1 | Glycerophospholipids | 1.674 | 0.744 | 1.841 | 0.008 | 0.148 |
| ACar 20:4 | Fatty Acyls | 1.607 | 0.684 | 1.788 | 0.010 | 0.158 |
| LPC 17:2 | Glycerophospholipids | 1.580 | 0.660 | 1.996 | 0.003 | 0.104 |
| LPC 18:3-sn1 | Glycerophospholipids | 1.789 | 0.839 | 2.124 | 0.002 | 0.104 |
| LPC 16:0-2 | Glycerophospholipids | 1.613 | 0.690 | 1.700 | 0.014 | 0.182 |
| LPC 18:1 | Glycerophospholipids | 1.516 | 0.600 | 1.942 | 0.004 | 0.128 |
| Corticosterone | Steroids and steroid derivatives | 1.543 | 0.625 | 1.850 | 0.007 | 0.148 |
| LPE 19:2 | Glycerophospholipids | 1.634 | 0.708 | 2.067 | 0.002 | 0.104 |
| PE 34:3e | Glycerophospholipids | 1.686 | 0.754 | 1.667 | 0.018 | 0.182 |
| ACar18:3 | Fatty Acyls | 1.572 | 0.653 | 1.790 | 0.010 | 0.158 |
| Retinol | Prenol lipids | 1.572 | 0.652 | 1.651 | 0.018 | 0.182 |
| Docosahexaenoic acid | Fatty Acyls | 2.356 | 1.237 | 1.760 | 0.012 | 0.166 |
| PC 38:7-sn1 | Glycerophospholipids | 1.633 | 0.707 | 1.660 | 0.018 | 0.182 |
